# Use of AI in Identification of Sexually Transmitted Infections and Anogenital Dermatoses: A Systematic Review and Meta-Analysis

**DOI:** 10.1001/jamanetworkopen.2025.33512

**Published:** 2025-10-03

**Authors:** Nyi Nyi Soe, Ingsun Isika Kusnandar, Phyu Mon Latt, Christopher K. Fairley, Eric P.F. Chow, Ismael Maatouk, Cheryl C. Johnson, Purvi Shah, Remco P.H. Peters, Lorenzo Subissi, Lei Zhang, Jason J. Ong

**Affiliations:** 1 Melbourne Sexual Health Centre, Alfred Health, Melbourne, Victoria, Australia; 2 School of Translational Medicine, Faculty of Medicine, Nursing and Health Sciences, Monash University, Melbourne, Victoria, Australia; 3 National School of Medicine, Doctor of Medicine, University of Notre Dame, Sydney, New South Wales, Australia; 4 Centre for Epidemiology and Biostatistics, Melbourne School of Population and Global Health, The University of Melbourne, Melbourne, Victoria, Australia; 5 Global HIV, Hepatitis and STIs Programmes, World Health Organization, Geneva, Switzerland; 6 UNAIDS Regional Support Team Asia Pacific, Bangkok, Thailand; 7 Health Emergency Preparedness and Response Programme, World Health Organization, Geneva, Switzerland; 8 China-Australia Joint Research Center for Infectious Diseases, School of Public Health, Xi’an Jiaotong University Health Science Center, Xi’an, China; 9 Phase I Clinical Trial Research Ward, The Second Affiliated Hospital of Xi’an Jiaotong University, Xi'an, China; 10 Department of Clinical Research, London School of Hygiene and Tropical Medicine, London, United Kingdom

## Abstract

**Importance:**

Artificial intelligence (AI) excels in dermatology. However, its applications to sexually transmitted infections (STIs) remain unclear.

**Objective:**

To assess the performance of AI algorithms and their applications in detecting STIs and anogenital dermatoses from clinical images in sexual health.

**Data Sources:**

Six databases (IEEE Xplore, Embase, Scopus, Medline, Web of Science, and CINAHL) were searched for studies published from January 1, 2010, to April 12, 2024, using 3 main concepts: *artificial intelligence*, *diagnosis*, and *sexually transmitted infections*.

**Study Selection:**

Studies that used AI to identify anogenital skin conditions from clinical images were included. Studies that used non-AI approaches or nonanogenital conditions, as well as reviews and studies lacking performance metrics, were excluded.

**Data Extraction and Synthesis:**

Following the Preferred Reporting Items for Systematic Reviews and Meta-Analyses (PRISMA) guidelines, 2 reviewers independently assessed full-text articles and extracted data using a standardized spreadsheet. Another 2 reviewers resolved any disagreements. A modified Quality Assessment of Diagnostic Accuracy Studies (QUADAS-2) critical appraisal tool and the Checklist for Evaluation of Image-Based AI Reports in Dermatology (CLEAR Derm) were used for quality assessment.

**Main Outcomes and Measures:**

Pooled sensitivity and specificity of AI applications for detecting anogenital skin conditions. A bivariate random-effects meta-analysis was conducted for conditions with more than 3 studies.

**Results:**

Of 5381 studies screened and 258 full texts selected, 140 met the inclusion criteria. Most studies reported on mpox (110 [78.6%]), while other anogenital conditions, including genital herpes (7 [5.0%]), genital warts (8 [5.7%]), scabies (8 [5.7%]), and molluscum contagiosum (6 [4.3%]), received less attention. Meta-analyses showed high performance of AI for identification of mpox (pooled sensitivity: 0.96 [95% CI, 0.93-0.97]; pooled specificity: 0.98 [95% CI, 0.97-0.99]), herpes simplex (sensitivity: 0.91 [95% CI, 0.71-0.98]; specificity: 0.97 [95% CI, 0.94-0.98]), genital warts (sensitivity: 0.87 [95% CI, 0.67-0.96]; specificity: 0.98 [95% CI, 0.95-0.99]), psoriasis (sensitivity: 0.90 [95% CI, 0.78-0.95]; specificity: 0.98 [95% CI, 0.96-0.99]), and scabies (sensitivity: 0.89 [95% CI, 0.84-0.93]; specificity: 0.98 [95% CI, 0.95-0.99]). Study quality was variable, and the assessment identified high risk of bias across the population selection (76.1%), reference standards (76.1%), and index tests (20.0%). Most studies relied on open-source datasets (121 [86.4%]); only 17 (12.1%) used external validation. All but 1 study (0.7%) remained at the proof-of-concept stage, and models were not publicly available for external evaluation.

**Conclusions and Relevance:**

The findings suggest that AI shows promise in identifying STIs and anogenital dermatoses but that significant research gaps exist. Future work should prioritize understudied STIs and differential conditions while improving data quality, conducting external validation, and validating findings in clinical settings.

## Key Points

**Question:**

What is the diagnostic performance of artificial intelligence (AI) in identifying sexually transmitted infections (STIs) and anogenital dermatoses from clinical images?

**Findings:**

In this systematic review and meta-analysis of 140 studies, research was dominated by mpox, while other STIs such as syphilis were understudied. AI showed high performance for identifying mpox, herpes simplex, genital warts, psoriasis, and scabies, but these findings were limited by high risk of bias and a lack of clinical validation.

**Meaning:**

The findings suggest that AI shows promise for identifying STIs and anogenital conditions, but research gaps in data quality, validation, and focus on clinically relevant STIs must be addressed to translate these tools into practice.

## Teaser

This systematic review and meta-analysis investigates the performance of artificial intelligence (AI) algorithms in detecting sexually transmitted infections and anogenital dermatoses from clinical images.

## Introduction

Sexually transmitted infections (STIs) profoundly affect sexual and reproductive health worldwide.^1^ The World Health Organization (WHO) estimated 374 million new cases of STIs in 2020: chlamydia (129 million), gonorrhea (82 million), syphilis (8 million), and trichomoniasis (156 million).^2^ These estimates do not include mpox, for which evidence has recently emerged showing the important role of sexual transmission in mpox outbreaks.^3^ Delayed diagnoses and untreated infections can result in serious complications, including a higher risk of HIV acquisition, infertility, and adverse pregnancy outcomes.^4^ The recent increase in syphilis cases across populations and resurgence of congenital syphilis highlight the urgent need for improved early identification and control measures for STIs.^5^

STIs can be asymptomatic or present with diverse clinical manifestations.^6^ Recent data showed that nearly half of sexual health clinic attendees were seen for anogenital conditions such as lumps, spots, rashes, sores, or itch.^7^ This complexity in clinical presentation poses a significant barrier to the timely diagnosis and management of STIs. Digital health interventions, particularly artificial intelligence (AI), have emerged as promising tools in sexual health. Machine learning approaches have been used to estimate HIV and other STI risks based on sexual behaviors.^8,9,10^ Machine learning and bayesian-based symptom checkers have been developed to assess the different presentations of STIs.^7,11^ The distinctive patterns of anogenital lesions suggest potential value in deep learning–based image classification for STI diagnosis.^12^ While AI has shown success in general dermatology, its application to STI-related dermatoses remains nascent, and its role in clinical implementation is unclear.^13^ This systematic review and meta-analysis aimed to evaluate the diagnostic performance of AI algorithms in classifying STIs and other anogenital dermatoses and provide evidence-based recommendations for advancing these technologies from research into clinical practice.

## Methods

### Study Design and Protocol

We conducted this systematic review and meta-analysis following the Preferred Reporting Items for Systematic Reviews and Meta-Analyses (PRISMA) reporting guideline.^14^ We preregistered our protocol in PROSPERO.^15^ The study team included sexual health specialists (C.K.F., I.M., C.C.J., P.S., R.P., L.S., J.J.O.), AI experts (N.N.S., P.M.L., L.Z.), and a biostatistician (E.P.F.C.).

### Search Strategy and Selection Criteria

We searched 6 databases (IEEE Xplore, Embase, Scopus, Medline, Web of Science, and CINAHL) for studies published from January 1, 2010, to April 12, 2024, using 3 main concepts: *artificial intelligence*, *diagnosis*, and *sexually transmitted infections* (eTable 1 in Supplement 1). We included studies ranging from proof-of-concept to randomized clinical trials without language restrictions. We included studies that used AI to identify or classify anogenital skin conditions using clinical images, with or without accompanying metadata. We excluded studies that did not involve AI-based approaches or anogenital conditions. We also omitted review articles and studies that did not report model performance metrics or that focused on dermatoscopic images and nonskin manifestations, such as cervical changes. Two reviewers (N.N.S., I.I.K.) independently screened titles and abstracts against our eligibility criteria after removing duplicates using Covidence software (Veritas Health Innovation).^16^ We conducted pilot testing before formal screening and data extraction to ensure consistency. After screening studies with 97% agreement, 2 additional reviewers (P.M.L., J.J.O.) resolved any remaining conflicts through consensus.

### Data Extraction

Two reviewers (N.N.S., I.I.K.) independently assessed full-text articles and extracted data using a standardized Microsoft Excel, version 16.100, spreadsheet (Microsoft Corporation). P.M.L. and J.J.O. resolved any disagreements in data extraction through discussion. We collected data on the following aspects: (1) study characteristics, including authors, publication year, and study type; (2) data characteristics, such as the image database, types of data input and predictions, reference conditions, and sample size; (3) technical information, including model development, evaluation methods, subgroup analyses, model interpretability, and the best-performing algorithms; and (4) performance metrics, such as area under the receiver operating characteristic curve (AUROC), accuracy, sensitivity, specificity, precision, F1 scores, and values from confusion matrices. We selected performance metrics from the best-performing model for studies reporting multiple models. We extracted true positives, false positives, true negatives, and false negatives directly from 2 × 2 tables for binary outcome studies. We recalculated these values from the tables, focusing on our target disease conditions in the studies with multiclass classifications.

### Quality Assessment

We used a modified Quality Assessment of Diagnostic Accuracy Studies (QUADAS-2) critical appraisal tool^17^ to independently assess the risk of bias and the applicability of included studies across the population, index test, and reference standards. We rated each domain as low, high, or uncertain risk based on the information provided in the studies. We also evaluated model fairness, reliability, and safety using the Checklist for Evaluation of Image-Based AI Reports in Dermatology (CLEAR Derm),^18^ scoring 25 items across data, technique, technical assessment, and application domains as “present,” “partially present,” “absent,” or “not applicable.” Disagreements were resolved through discussion among the review team (N.N.S., I.I.K., P.M.L., J.J.O.).

### Statistical Analysis

We conducted a descriptive analysis of study characteristics and model performance. One study^19^ was excluded from the analysis due to retraction,^20^ and we reconducted the analysis after removing data from this study. We used dupeGuru, version 4.0.3 (Hardcoded Software), to identify duplicate images across public datasets. We conducted a bivariate random-effects meta-analysis for conditions with more than 3 studies. We generated forest plots and summary receiver operating characteristic (ROC) graphs. We used funnel plots of diagnostic odds ratios and the Deeks regression test to assess publication bias. We conducted a meta-regression analysis on mpox studies using the Higgins *I*^2^ statistic to assess heterogeneity based on sample size, number of reference skin conditions, type of model classification, and AI algorithm category. We measured the *F* statistics, and 2-sided *P* < .05 was used to indicate the significance of the regression models. We analyzed all data using Stata, version 17 (StataCorp LLC).^21^

## Results

From 6035 initial studies, we screened 5381 titles and abstracts and selected 258 full texts. After removing irrelevant studies, duplicates, and review articles, 140 studies met the inclusion criteria (Figure).^22,23,24,25,26,27,28,29,30,31,32,33,34,35,36,37,38,39,40,41,42,43,44,45,46,47,48,49,50,51,52,53,54,55,56,57,58,59,60,61,62,63,64,65,66,67,68,69,70,71,72,73,74,75,76,77,78,79,80,81,82,83,84,85,86,87,88,89,90,91,92,93,94,95,96,97,98,99,100,101,102,103,104,105,106,107,108,109,110,111,112,113,114,115,116,117,118,119,120,121,122,123,124,125,126,127,128,129,130,131,132,133,134,135,136,137,138,139,140,141,142,143,144,145,146,147,148,149,150,151,152,153,154,155,156,157,158,159,160^

**Figure.**
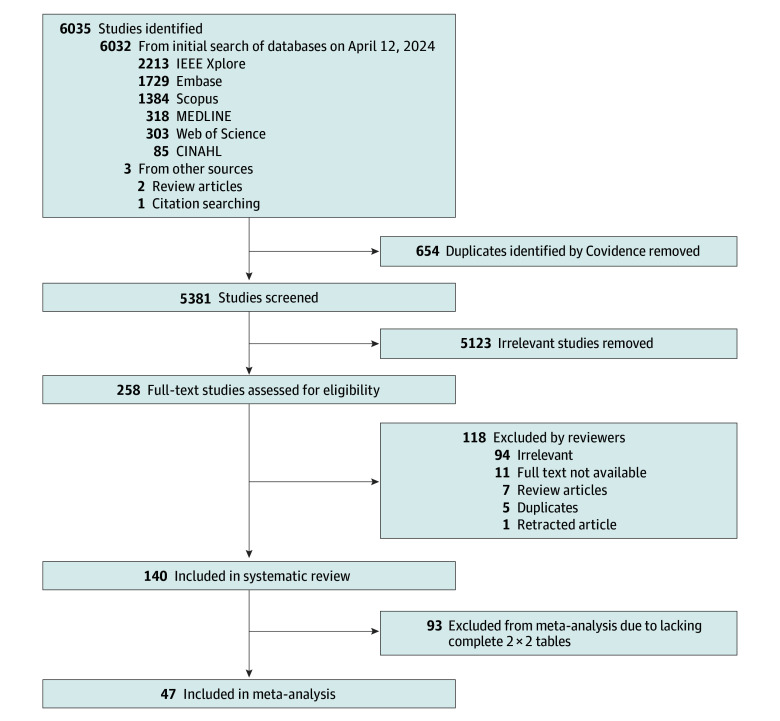
Preferred Reporting Items for Systematic Reviews and Meta-Analyses (PRISMA) Flow Diagram of the Study Selection Process

### Quality Assessment

Using the modified QUADAS-2 tool, we identified a high level of bias in most studies regarding 3 domains: populations (76.1%), reference standard tests (76.1%), and index tests (20.0%). We also identified a high concern for applicability: populations (82.2%), index tests (80.7%), and reference standard tests (76.1%). The CLEAR Derm checklist showed inadequate reporting across all domains (eFigure 1 and eTable 3 in Supplement 1).

### Disease Conditions

Most studies (110 [78.6%]) focused on mpox following the 2022 outbreak.^19,25,29,30,33,35,44,45,46,47,48,49,50,51,52,53,54,55,56,57,58,59,60,61,62,63,64,65,66,67,68,69,70,71,72,73,74,75,76,77,78,79,80,81,82,83,84,85,86,87,88,89,90,91,92,93,94,95,96,97,98,99,100,101,102,103,104,105,106,107,108,109,110,111,112,113,114,115,116,117,118,119,120,121,122,123,124,125,126,127,128,129,130,131,132,133,134,135,136,137,138,139,140,141,142,143,144,145,146,147,148^ Other conditions (tinea cruris [8 studies (5.7%)^22,23,24,28,37,149,150,151^], genital warts [8 (5.7%)^22,23,26,32,34,37,42,43^], scabies [8 (5.7%)^31,37,40,125,150,154,155,156^], herpes zoster [8 (5.7%)^23,24,36,149,157,158,159,160^], psoriasis [7 (5.0%)^22,23,26,34,150,152,156^], herpes simplex [7 (5.0%)^22,32,37,38,39,40,41^], lichenoid changes [6 (4.3%)^23,24,26,37,152,153^], and molluscum contagiosum [6 (4.3%)^22,23,26,37,125,149^]) received limited attention. The types of target and reference conditions included in the studies ranged from 2^44,46,47,48^ to 44.^26^ Most mpox studies (95 [87.9%]) focused on a limited number of differential diagnoses, primarily comparing mpox with conditions that are unrelated to common anogenital dermatoses typically seen in sexual health clinics (eTable 2 in Supplement 1).^25,29,33,35,44,45,46,47,48,49,50,51,52,53,55,56,57,58,59,60,62,63,64,65,66,67,68,69,70,71,72,73,74,75,76,77,78,79,80,81,82,83,84,85,86,87,88,89,90,91,92,93,94,95,96,97,98,99,100,101,102,103,104,105,106,107,108,109,110,111,112,113,115,116,117,118,119,120,121,122,123,125,126,127,128,129,130,131,132,133,134,135,136,137,138,139,140,141,142,143,144,145,146,147,148^ Only 18 studies (12.9%) incorporated clinically relevant differential conditions,^22,23,24,26,27,28,32,37,40,65,72,124,125,149,150,155,156,158^ and only 6 (4.3%) provided a broader comparison with multiple anogenital dermatoses.^50,51,52,53,54,55^

### Data

Most studies used open-source data (121 [86.4%]^22,24,29,35,39,42,43,44,45,46,48,49,50,51,52,53,54,55,56,57,58,59,60,61,62,63,64,65,66,68,69,70,71,72,73,74,75,76,77,79,80,81,82,83,84,85,88,89,91,92,93,94,95,96,97,99,100,101,102,103,104,105,106,108,109,110,111,112,113,115,116,117,118,120,121,123,124,125,126,127,128,129,130,131,134,135,136,137,138,139,140,141,142,143,144,145,146,147,148,149,151,153,154,156,157,159,160^), while a few used private databases (12 [8.6%]^23,26,27,28,31,32,34,37,38,150,155,158^) or a mixed dataset (5 [3.5%]^25,30,33,36,90^) (eTable 4 in Supplement 1). Nearly three-quarters of studies (103 [73.6%]) properly referenced the image databases.^18,23,24,25,29,30,33,35,36,39,41,42,43,44,45,46,47,48,49,50,51,52,53,54,56,57,58,59,60,61,62,64,66,67,68,69,70,71,72,73,74,75,76,77,79,80,81,82,83,84,90,91,92,94,100,101,102,103,104,105,106,107,108,109,110,111,112,113,114,115,116,117,122,123,124,125,126,128,129,130,131,134,135,136,137,138,139,142,143,144,146,147,148,151,154,156,157,160^ The most frequently used public databases for mpox studies were MSLD, MSID, and MCSI,^161,162,163^ where 84 of 1155 images (7.3%) were identified as duplicates (eTable 5 in Supplement 1). The 12 private datasets originated mainly from Asia (4 [33.3%]: China, 1 [8.3%]^24^; India, 2 [16.7%]^23,37^; the Philippines, 1 [8.3%]^34^) and the Americas (3 [25.0%]: US, 2 [16.7%]^30,39^; Peru, 1 [8.3%]^159^). The image sample size varied substantially across different datasets, ranging from 70 to 139 198. Studies showed considerable class imbalance, where the proportion of target condition images ranged from 0.1% to 71.8% of total images within datasets (median, 36.2% [IQR, 19.9%-44.7%]).

Most datasets that were used contained clinical images alone; datasets of only 8 studies (5.7%) contained additional patient metadata (sex, race, ethnicity, symptoms, etc).^52,54,55,59,60,61,62,63^ Eight studies (5.7%) reported skin tones.^33,52,54,59,61,62,64,65^ Only 15 studies (10.7%) reported technical details about image acquisition, such as image quality, camera type, or lighting conditions.^22,24,25,26,27,30,31,36,37,43,72,86,106,150,159^ Only 17 studies (12.1%) validated image diagnoses using laboratory tests, clinician-reviewed images, or both.^23,24,25,26,27,28,30,31,32,34,37,55,90,101,142,158,159^ Three studies (2.1%) standardized their image categories using *International Classification of Diseases, Ninth Revision (ICD-9)* and *International Statistical Classification of Diseases and Related Health Problems, 10th Revision (ICD-10)* codes.^52,54,59^

### Model Development

Over half of the studies (78 [55.7%]) focused on developing binary classification models,^23,25,27,33,34,35,36,44,45,46,47,48,49,50,52,53,54,56,59,60,61,64,65,66,68,69,74,75,77,79,80,85,88,89,91,92,93,95,97,99,100,102,104,105,106,108,111,112,115,117,118,120,123,124,127,128,129,130,134,135,140,141,144,145,148,152,155,157,159,160^ while 57 studies (40.7%) used multiclass classification approaches.^22,24,26,28,29,30,31,32,37,38,40,41,42,43,51,55,58,62,63,68,70,71,72,73,76,81,82,83,84,88,90,94,96,101,103,113,115,116,121,125,126,136,137,138,139,142,143,146,147,149,150,151,153,154,156,158^ Only 2 studies (1.4%) incorporated images and patient metadata,^55,59^ while all other studies used solely image data input. All studies except 17 (12.1%)^26,37,54,65,66,74,85,87,97,102,114,135,138,141,144,145,156^ reported image preprocessing procedures such as resizing, normalization, cropping, and rotation to address class imbalance, but none used synthetic images.

### Model Evaluation

All but 14 studies (10.0%) reported fully or partially splitting their data into training and testing sets,^26,37,39,70,84,95,121,124,127,130,146,148,149,159^ while only 35 studies (25.0%) used *k*-fold cross-validation for more robust validation.^23,25,27,30,33,45,53,55,57,61,62,68,72,75,80,81,83,89,90,97,100,102,105,106,108,110,117,123,136,139,142,150,152,155,158^ Eight studies (5.7%) assessed model generalizability using external validation or prospectively collected image datasets,^23,24,25,26,28,30,37,84^ and only 3 studies (2.1%) used multisite data.^59,61,66^ While 6 studies (4.3%) considered gender in their analyses,^24,27,28,30,32,37^ only 1 study (0.7%) specifically evaluated model performance for females.^28^ Only a few studies evaluated model performance across different subgroups: skin tone (5 [3.6%]^24,28,30,33,34^), lesion site (5 [3.6%]^24,26,27,28,30^), age (4 [2.9%]^24,27,28,30^), and race and ethnicity (2 [1.4%]^28,30^). Only 24 studies (17.1%) used interpretability techniques (gradient-weighted class activation mapping, local interpretable model-agnostic explanations, and Shapley additive explanations) to visualize decision-making features.^24,25,28,30,32,33,46,47,48,59,60,62,67,68,72,79,96,106,113,114,115,123,125,136^

### Model Performance

We summarized model performance metrics by target condition (Table 1). Studies commonly reported accuracy, sensitivity, specificity, positive predictive values, and F1 scores, while negative predictive values and AUROC scores were less frequently reported. Studies demonstrated high mean accuracy (>70.0%) for syphilis, herpes simplex, genital warts, mpox, scabies, herpes zoster, and penile cancer, while other conditions showed lower accuracy (ranging from 42.0% to 65.0%). Most conditions achieved high specificity (>95.0%), but other metrics showed considerable variation within conditions. For example, accuracy in herpes simplex models ranged from 10.0%^22^ to 97.0%.^41^ The highest performance was achieved with convolutional neural network–based architectures (96 studies [68.6%]),^23,25,28,29,30,31,33,35,37,39,41,44,45,46,47,48,49,51,52,53,55,56,62,64,65,66,67,68,69,71,72,73,74,75,76,78,79,80,83,86,87,88,89,90,91,92,93,94,95,96,97,98,99,100,101,106,107,109,110,111,114,115,116,117,119,120,121,122,124,125,126,127,128,129,130,131,132,133,134,135,136,137,139,140,141,142,144,145,147,148,151,153,154,156,158,159,160^ followed by hybrid or ensemble models (29 [20.7%]^27,32,36,40,43,50,57,59,60,61,63,70,77,81,82,84,85,91,96,102,105,108,110,113,118,123,138,143,146^).

**Table 1.** Summary of Model Performance Across Disease Conditions

Disease condition	Sensitivity	Specificity	PPV	NPV	AUROC	Accuracy	F1 score
Syphilis (chancre, condylomata lata) (n = 1)							
Metric, mean (SD)	0.87 (NA)	0.99 (NA)	0.91 (NA)	NA	NA	0.94 (NA)	0.95 (NA)
Studies, No.	1	1	1	1	1	1	1
Herpes simplex (n = 7)							
Metric							
Mean (SD)	0.58 (0.45)	0.98 (0.02)	0.63 (0.46)	NA	1.00 (NA)	0.74 (0.30)	0.66 (0.48)
Median (IQR)	0.62 (0.20-0.96)	0.98 (0.97-0.99)	0.87 (0.10-0.93)	NA	NA	0.84 (0.69-0.94)	0.92 (0.10-0.96)
Range	0.10-0.99	0.97-0.99	0.10-0.93	NA	NA	0.10-0.97	0.10-0.96
Studies, No.	4	2	3	0	1	7	3
Genital warts (n = 8)							
Metric							
Mean (SD)	0.82 (0.20)	0.99 (0.02)	0.69 (0.37)	1.00 (NA)	0.85 (NA)	0.86 (0.15)	0.96 (0.05)
Median (IQR)	0.93 (0.57-0.96)	1.00 (0.96-1.00)	0.86 (0.57-0.94)	NA	NA	0.94 (0.69-0.97)	0.97 (0.91-1.00)
Range	0.56-1.00	0.96-1.00	0.10-1.00	NA	NA	0.65-0.98	0.91-1.00
Studies, No.	6	3	5	1	1	6	3
Mpox (n = 110)							
Metric							
Mean (SD)	0.92 (0.09)	0.93 (0.09)	0.93 (0.06)	0.94 (0.09)	0.96 (0.06)	0.93 (0.06)	0.92 (0.08)
Median (IQR)	0.94 (0.88-1.00)	0.97 (0.90-0.99)	0.95 (0.91-1.00)	0.98 (0.93-0.99)	0.98 (0.94-1.00)	0.95 (0.91-1.00)	0.94 (0.90-1.00)
Range	0.58-1.00	0.61-1.00	0.70-1.00	0.79-1.00	0.74-1.00	0.74-1.00	0.67-1.00
Models, No.	114	37	102	5	37	127	107
Tinea cruris (n = 8)							
Metric							
Mean (SD)	0.53 (0.39)	0.99 (0.01)	0.28 (0.25)	1.00 (NA)	0.98 (NA)	0.62 (0.36)	0.52 (0.60)
Median (IQR)	0.64 (0.10-0.85)	0.99 (0.99-1.00)	0.28 (0.10-0.45)	NA	NA	0.72 (0.40-0.84)	0.52 (0.10-0.94)
Range	0.10-0.85	0.99-1.00	0.10-0.45	NA	NA	0.10-0.92	0.10-0.94
Studies, No.	3	2	2	1	1	4	2
Molluscum contagiosum (n = 6)							
Metric							
Mean (SD)	0.55 (0.38)	0.98 (0.02)	0.59 (0.43)	0.98 (NA)	0.95 (NA)	0.53 (0.35)	0.51 (0.58)
Median (IQR)	0.55 (0.23-0.92)	0.98 (0.97-0.99)	0.76 (0.10-0.92)	NA	NA	0.55 (0.25-0.80)	0.51 (0.10-0.92)
Range	0.10-0.93	0.97-0.99	0.10-0.92	NA	NA	0.10-0.92	0.10-0.92
Studies, No.	6	2	3	1	1	4	2
Lichenoid changes (n = 6)							
Metric							
Mean (SD)	0.59 (0.30)	0.97 (0.01)	0.71 (0.26)	0.99 (NA)	0.88 (NA)	0.65 (0.25)	0.83 (0.24)
Median (IQR)	0.57 (0.56-0.68)	0.97 (0.97-0.98)	0.64 (0.49-1.00)	NA	NA	0.69 (0.53-0.73)	0.83 (0.66-1.00)
Range	0.16-1.00	0.97-0.98	NA	NA	NA	0.31-0.99	0.66-1.00
Studies, No.	5	2	3	1	1	5	2
Scabies (n = 8)							
Metric							
Mean (SD)	0.83 (0.16)	1.00	0.89 (0.13)	NA	NA	0.85 (0.10)	NA
Median (IQR)	0.86 (0.71-0.95)	NA	0.89 (0.80-0.98)	NA	NA	0.85 (0.77-0.92)	NA
Range	0.63-0.98	NA	0.80-0.98	NA	NA	0.69-0.99	NA
Studies, No.	4	1	2	0	0	7	0
Folliculitis (n = 3)							
Metric							
Mean (SD)	0.36 (0.35)	NA	NA	NA	NA	0.42 (0.24)	NA
Median (IQR)	0.36 (0.11-0.60)	NA	NA	NA	NA	0.55 (0.14-0.57)	NA
Range	0.11-0.60	NA	NA	NA	NA	0.14-0.57	NA
Studies, No.	2	0	0	0	0	3	0
Herpes zoster (n = 8)							
Metric							
Mean (SD)	0.82 (0.13)	0.98 (0.03)	0.85 (0.19)	1.00 (NA)	0.94 (0.05)	0.94 (0.03)	0.95 (0.04)
Median (IQR)	0.83 (0.71-0.91)	1.00 (0.94-1.00)	0.93 (0.74-0.96)	NA	0.93 (0.89-0.99)	0.95 (0.92-0.96)	0.95 (0.92-0.99)
Range	0.67-0.96	0.94-1.00	0.57-0.97	NA	0.89-0.99	0.90-0.97	0.92-0.99
Studies, No.	5	3	4	1	3	5	3
Psoriasis (n = 7)							
Metric							
Mean (SD)	0.72 (0.35)	0.96 (NA)	0.65 (0.40)	0.98 (NA)	0.93 (NA)	0.58 (0.41)	0.55 (0.64)
Median (IQR)	0.84 (0.78-1.00)	NA	0.75 (0.36-1.00)	NA	NA	0.62 (0.25-0.92)	0.55 (0.10-1.00)
Range	0.10-1.00	NA	0.10-1.00	NA	NA	0.10-0.99	0.10-1.00
Studies, No.	5	1	4	1	1	4	2
Candidiasis (n = 1)							
Metric, mean (SD)	0.41 (NA)	1.00 (NA)	0.41 (NA)	1.00 (NA)	0.81 (NA)	NA	NA
Studies, No.	1	1	1	1	1	0	0
Balanitis (n = 2)							
Metric							
Mean (SD)	0.88 (NA)	1.00 (NA)	0.97 (NA)	NA	NA	0.64 (0.43)	0.98 (NA)
Median (IQR)	NA	NA	NA	NA	NA	0.64 (0.33-0.94)	NA
Range	NA	NA	NA	NA	NA	0.33-0.94	NA
Studies, No.	1	1	1	0	0	2	1
Penile cancer (n = 1)							
Metric, mean (SD)	0.79 (NA)	0.99 (NA)	0.89 (NA)	NA	NA	0.94 (NA)	0.93 (NA)
Studies, No.	1	1	1	0	0	1	1
STIs (n = 1)							
Metric, mean (SD)	0.95 (NA)	0.62 (NA)	0.43 (NA)	NA	0.89 (NA)	0.69 (NA)	NA
Studies, No.	1	1	1	0	1	1	1

Abbreviations: AUROC, area under the receiver operating characteristic curve; NA, not applicable; NPV, negative predictive value; PPV, positive predictive value; STI, sexually transmitted infection.

### Application

All of the studies but 1 (0.7%)^26^ remained at the proof-of-concept stage without publicly available models for external evaluation. Those studies also did not specify their intended users (clinicians or the public) or the tool’s purpose (such as triage, assisted diagnosis, or population screening). Only 1 study (0.7%) tested a public-facing skin-imaging application with prospectively collected images.^26^ No studies evaluated model accuracy in clinical settings using randomized trials.

### Meta-Analysis

For meta-analysis, we included 47 studies: 31 mpox studies (66.0%; 34 contingency tables)^25,30,35,68,72,83,88,90,96,97,105,106,110,111,113,115,116,117,123,125,126,129,136,143,144^ and 4 studies (8.5%) each for herpes simplex,^38,39,40,41^ genital warts,^23,32,34,43^ psoriasis,^23,34,150,156^ and scabies^40,125,150,156^ (Figure). We could not pool the sensitivity and specificity for other conditions due to insufficient data points. Models showed consistently high performance across conditions: mpox (pooled sensitivity: 0.96 [95% CI, 0.93-0.97]; *I*^2^, 90.98 [95% CI, 88.74-93.22]; pooled specificity: 0.98 [95% CI, 0.97-0.99]; *I*^2^, 99.90 [95% CI, 99.90-99.91]), herpes simplex (sensitivity: 0.91 [95% CI, 0.71-0.98]; *I*^2^, 93.21 [95% CI, 88.22-98.21]; specificity: 0.97 [95% CI, 0.94-0.98]; *I*^2^, 72.62 [95% CI, 44.18-100.00]), genital warts (sensitivity: 0.87 [95% CI, 0.67-0.96]; *I*^2^, 87.40 [95% CI, 76.42-98.37]; specificity: 0.98 [95% CI, 0.95-0.99]; *I*^2^, 93.88 [95% CI, 89.50-98.25]), psoriasis (sensitivity: 0.90 [95% CI, 0.78-0.95]; *I*^2^, 92.83 [95% CI, 87.47-98.19]; specificity: 0.98 [95% CI, 0.96-0.99]; *I*^2^, 92.11 [95% CI, 86.05-98.17]), and scabies (sensitivity: 0.89 [95% CI, 0.84-0.93]; *I*^2^, 69.12 [95% CI, 36.37-100.00]; specificity: 0.98 [95% CI, 0.95-0.99]; *I*^2^, 83.77 [95% CI, 68.72-98.82]). There was no evidence of publication bias (Deeks funnel plot asymmetry test). Forest plots, summary ROC graphs, and Deeks funnel plots are presented in eFigure 2 in Supplement 1.

We conducted subgroup analyses and meta-regression for heterogeneity only for mpox studies, as there were limited studies and insufficient data to perform subgroup analysis for other conditions (Table 2). Meta-regression identified sample size and model classification as the most significant factors associated with mpox model performance. Larger datasets (≥1000 vs <1000 images) and binary vs multiclass classification approaches demonstrated significantly higher pooled sensitivity (0.97 [95% CI, 0.92-0.99]; *P* = .049 and 0.97 [95% CI, 0.95-0.99]; *P* = .03, respectively). Based on our systematic review and meta-analysis findings, we summarized key research gaps and limitations in existing studies and formulated recommendations for future research, as presented in Table 3.^6,18,27,28,164,165,166,167,168,169,170,171,172,173,174,175,176,177,178,179,180,181^

**Table 2.** Summary Estimates of Pooled Performance of AI Models for Mpox

Characteristic	Models, No. (%)	Model *F*_4,29_ = 2.53 (*P* = .06)				Model *F*_4,29_ = 0.70 (*P* = .60)			
		Sensitivity	*P* value^a^	*I*^2^ (95% CI)^b^	*P* value^c^	Specificity	*P* value^a^	*I*^2^ (95% CI)^b^	*P* value^c^
Overall	34 (100)	0.96 (0.93-0.97)	<.001	90.98 (88.74-93.22)	NA	0.98 (0.97-0.99)	<.001	99.90 (99.90-99.91)	NA
Lesion types included, No.									
<5	26 (76.5)	0.96 (0.93-0.98)	<.001	89.86 (86.87-92.85)	.13	0.98 (0.97-0.99)	<.001	92.84 (90.93-94.74)	.96
≥5	8 (23.5)	0.93 (0.84-0.97)	<.001	93.47 (90.31-96.64)		0.97 (0.95-0.99)	<.001	99.95 (99.95-99.96)	
Sample size									
<1000	23 (67.6)	0.95 (0.91-0.97)	<.001	88.49 (84.74-92.24)	.049	0.98 (0.97-0.99)	<.001	91.29 (88.67-93.91)	.53
≥1000	11 (32.4)	0.97 (0.92-0.99)	<.001	94.07 (91.73-96.42)		0.97 (0.95-0.99)	<.001	99.96 (99.96-99.97)	
Model classification									
Binary	17 (50.0)	0.97 (0.95-0.99)	<.001	87.87 (83.16-92.57)	.03	0.98 (0.95-0.99)	<.001	92.43 (89.87-94.98)	.20
Multiclass	17 (50.0)	0.93 (0.88-0.96)	<.001	89.02 (84.89-93.15)		0.98 (0.97-0.99)	<.001	99.95 (99.95-99.95)	
AI algorithm									
CNN based	26 (76.5)	0.95 (0.92-0.97)	<.001	91.31 (88.86-93.76)	.54	0.98 (0.96-0.99)	<.001	99.91 (99.91-99.92)	.40
Other	8 (23.5)	0.95 (0.89-0.98)	<.001	88.42 (81.81-95.04)		0.98 (0.97-0.99)	.050	50.25 (10.19-90.31)	

Abbreviations: AI, artificial intelligence; CNN, convolutional neural network.

^a^ *P* value for heterogeneity within each subgroup.

^b^ Higgins *I*^2^ statistic, a measure of heterogeneity.

^c^ *P* value for heterogeneity between subgroups with metaregression analysis.

**Table 3.** Summary of Research Gaps and Future Recommendations for AI-Based Identification of STIs and Anogenital Dermatoses

Research gap or limitation		Recommendations
**1. Disease conditions**		
1.1	Imbalanced research focus: Studies predominantly focused on mpox (78.6%) due to the recent outbreak, while common STIs and anogenital conditions received limited attention (<6.0% each)	Balanced research agenda: Prioritize AI research into common STIs and anogenital conditions while continuing to investigate emerging outbreaks like mpox, with an emphasis on WHO-priority infections such as syphilis, genital herpes, and genital warts
1.2	Limited differential coverage: Studies lacked clinically relevant comparative conditions and comprehensive coverage of anogenital conditions as seen in sexual health clinical practice	Diverse and representative data: Include a wide variety of anogenital reference conditions covering STIs, non-STIs, tumors, inflammatory diseases, and normal anatomic variants for robust differential diagnosis^6,164,165,166^
1.3	Poor disease standardization: Lack of standardized disease definition undermined clinical relevance and hindered reproducibility (eg, herpes simplex vs herpes zoster; genital warts vs flat warts)	Adoption of *ICD-10* systems: Use standardized *ICD-10* codes for labeling and disease categorization in datasets^18^
**2. Data**		
2.1	Data scarcity challenges: Models relied heavily on open-source datasets (86.5%), with small sample sizes, limited representation of target conditions, and absence of patient metadata^167^	Coordinated data infrastructure: Establish networks for standardized image collection and develop a centralized repository (similar to the IARC Cervical Cancer Image Bank^168^) for STIs and anogenital conditions
2.2	Data quality concern: Duplicate images across public datasets compromised data quality^169,170^	Implementation of deduplication processes: Use systematic methods or tools to identify and remove duplicate images to ensure data quality Transparent reporting: Document and transparently report all methods for image quality control^171^
2.3	Inadequate technical documentation: Most studies (89.4%) lacked essential details regarding image acquisition methods and quality standards	Technical standardization: Adopt and adapt established technical guidelines to ensure consistent quality in clinical image acquisition^172^ Documentation and reporting for reproducibility: Provide detailed documentation of image acquisition methods, quality standards, and technical specifications^172^
2.4	Insufficient diagnostic validation: Limited validation of image diagnoses through laboratory tests or clinical reviews undermined data reliability	Diagnostic validation standards: Require validation of image diagnoses through laboratory confirmation and/or expert clinical review before inclusion in datasets^6^
**3. Model development**		
3.1	Unclear clinical alignment: Model development lacked clear alignment with intended clinical applications	Purpose-driven development: Define clear clinical objectives (rule in or rule out) and model approach (binary or multiclass) based on intended clinical use (screening, triage, or assisted diagnosis)^173^ Interdisciplinary collaboration: Ensure development teams include data scientists, AI experts, sexual health physicians, and end users^173,174^
3.2	Inadequate reporting of methods: Insufficient documentation of data splitting and cross-validation approaches, including crucial stratification methods with patient metadata (sex, lesion site, skin tone, etc) and diagnoses	Data splitting integrity: Prevent data leakage by ensuring images from the same patient remain in the same dataset split Stratified validation protocols: Implement stratified data splitting and cross-validation based on patient characteristics and diagnoses
3.3	Underused multimodal approach: Most models used only image data, with minimal integration of patient metadata (1.4%), despite the potential benefits of a multimodal approach^27,28^	Multimodal integration: Develop models that combine clinical images with relevant patient metadata to improve diagnostic accuracy
**4. Model evaluation**		
4.1	Limited generalizability: Model generalizability was limited by a lack of external validation and clinical trials, with most studies being single-center evaluations	Model availability: Encourage public accessibility of trained models to facilitate external validation Multicenter validation: Establish collaborative networks for prospective model testing across various clinical settings
4.2	Limited subgroup assessment: Minimal evaluation across genders, skin tones, lesion sites, ages, races, and ethnicities	Gender-specific models: Develop dedicated models specifically for females to address unique anatomic features and disease presentations Diverse subgroup analysis: Evaluate model performance across different demographic groups, anatomic sites, and clinical presentations^175^
4.3	Unexplained model decisions: Limited reporting of model interpretability techniques (17.0%), leaving model decision processes unclear	Model transparency with visualization: Implement modern interpretability techniques (eg, Grad-CAM, LIME, SHAP) to explain the model’s decision-making processes
**5. Model performance**		
5.1	Inconsistent performance reporting: Inconsistent reporting performance metrics hindered model comparison	Standardized performance reporting: Document key performance metrics (AUROC, sensitivity, specificity, PPV, and F1 scores) and contingency tables on test datasets to enable meaningful comparisons
5.2	Unclear threshold criteria: Binary classification studies lacked specified threshold selection criteria for sensitivity and specificity trade-offs based on intended use	Performance trade-off reporting: Specify how sensitivity and specificity thresholds were optimized for the intended clinical application
5.3	Variable model performance: Performance varied widely due to differences in model architectures, purposes, and reference condition selections	Facilitation of evidence synthesis: Conduct more studies on prioritized diseases using a standardized reporting framework to generate robust evidence, enabling meta-analysis to derive stronger conclusions on model accuracy and clinical utility
**6. Application**		
6.1	Limited translation to practice: Studies remained at the proof-of-concept stage without defined clinical purpose (triage or screening) or target users (clinicians or public)^176^	Bridging of research and practice: Encourage collaborations between researchers, health care practitioners, and policy makers to translate proof-of-concept studies into clinically viable solutions Development of an implementation framework: Establish frameworks to guide from the development stage to integration of AI models into existing health care workflows with a multidisciplinary team^173,177^
6.2	Minimal clinical implementation: Only 1 public-facing application was tested prospectively, with no RCTs conducted	Testing beyond conceptualization: Advance AI models from the conceptual stage by conducting pilot testing and clinical trials to generate robust evidence on their safety, efficacy, and clinical utility^178^
6.3	Limited study on user’s perspective: Limited evidence on the needs, expectations, and experience of end users, such as clinicians and patients, in adopting AI models for implementation	Incorporation of usability testing: Conduct feasibility, acceptability, and usability studies to ensure models meet the practical requirements of their target users in clinical or public health settings^179,180,181^

Abbreviations: AI, artificial intelligence; AUROC, area under the receiver operating characteristic curve; IARC, International Agency for Research on Cancer; *ICD-10*, *International Statistical Classification of Diseases and Related Health Problems, 10th Revision*; Grad-CAM, gradient-weighted class activation mapping; LIME, local interpretable model-agnostic explanations; PPV, positive predictive value; RCT, randomized clinical trial; SHAP, Shapley additive explanations; STI, sexually transmitted infection; WHO, World Health Organization.

## Discussion

Across 6 databases, we identified 140 eligible studies that used AI to detect or classify STIs and anogenital dermatoses. Anogenital conditions like syphilis, genital herpes, and other common differential dermatoses in sexual health have received limited attention. Meta-analysis demonstrated promising performance metrics, with pooled sensitivity above 0.87 and pooled specificity above 0.97 for conditions including mpox, herpes simplex, genital warts, psoriasis, and scabies. However, the high heterogeneity (*I*^2^ > 50.00) across studies suggests that these results should be interpreted with caution, as the variability could result from differences in methods, such as diagnostic thresholds, reference standards, and study populations, rather than actual performance differences in AI models.^182^ Quality assessment using the modified QUADAS-2 tool indicated high concerns for risk of bias and applicability across studies, while the CLEAR Derm checklist highlighted incomplete reporting of data characteristics, techniques used in the methods, and clinical validation.^18^ These quality assessment results align with similar AI models in dermatology conditions.^13^ Moreover, insufficient external validation and the lack of prospective testing present significant barriers to translating these AI tools from proof-of-concept to clinical practice. In this systematic review and meta-analysis, we discussed these research gaps and provided recommendations for future studies to enhance the clinical utility of AI in identification and management of STIs and anogenital dermatoses.

Although AI in dermatology shows promise, its use for STIs and anogenital dermatoses remains limited.^13^ Before 2022, few studies included anogenital dermatoses in their analyses as either target or differential diagnoses. While the 2022 mpox outbreak sparked numerous research studies, other common STIs have received limited research attention, despite their increasing global prevalence in recent years. While AI holds promise for early identification of syphilis chancres, only 2 studies addressed this application with limited sample sizes until 2024.^27,32^ The clinical relevance of current studies is further constrained by the comparative conditions they included. Most mpox studies, for instance, developed models to distinguish mpox from other conditions, such as chickenpox and measles, which are rarely seen in sexual health clinics. This limited coverage of relevant anogenital conditions hinders the practical utility of these models in clinical settings. The absence of normal anatomic variants (such as skin tags and Fordyce spots) in training datasets could lead models to misclassify these as pathologic conditions like genital warts, potentially causing unnecessary concern. Therefore, future studies should prioritize clinically important STIs and incorporate a comprehensive range of relevant conditions in model development to enhance their clinical utility.^6,164,165,166^

The poor quality of data represents a fundamental barrier to the development of reliable AI, and our systematic review and meta-analysis highlighted critical challenges regarding data scarcity, quality, and validity. The heavy reliance on limited open-source datasets, the duplication of images, and, most importantly, the lack of clinical validation for image diagnoses raise concerns about the generalizability of reported model performance. Images of anogenital dermatoses are particularly scarce compared with other dermatologic conditions.^167^ Difficulty in extracting data from clinical notes and privacy and anonymity concerns have resulted in most images lacking essential patient metadata, such as age and symptoms (pain, duration, etc), which are crucial for clinical decision-making. Establishing a centralized repository like the Cervical Cancer Image Bank,^168^ with a standardized image collection protocol for STIs and anogenital dermatoses, could address the current challenges.

Most included studies focused on technical aspects of data processing and algorithm design yet overlooked 2 important areas. First, studies rarely defined their target users (the general public or health care practitioners) and use-case scenarios (self-symptom checking or clinical diagnosis support). Clear alignment between model design and clinical application could be achieved by a multidisciplinary team approach (data scientists, AI experts, sexual health physicians, and end users) and following the OPTICA tool.^173^ Second, the predominant use of open-source or single-center data without external validation or prospective testing raises concerns about model generalizability. The limited evaluation across different demographic groups, particularly regarding gender, skin tones, and anatomic sites, suggests potential performance disparities across diverse populations.^172,175^ Future studies should prioritize external validation using diverse datasets, comprehensive demographic evaluation, and transparent reporting to improve the generalizability of AI models.

Most studies remained at the conceptual stage with limited translation into clinical applications. However, this technical focus should not preclude the exploration of end-user perspectives, which are crucial for successful implementation. A few studies explored the public’s acceptability, feasibility, and preferences for the tool when it becomes available. For example, Ly et al^179^ found that nearly 40% of users were reluctant to share clinical images for AI-based health care tools, particularly genital images due to privacy concerns. In contrast, Jakob et al^183^ reported high interest in STI-related apps among outpatients with dermatologic or venereal conditions. Soe et al^180^ also found that sexual health clinic attendees were willing to use such apps and provide comprehensive information, including symptoms and sexual behaviors, along with anogenital lesion images, if the app was developed by a reputable organization and demonstrated reliable accuracy. Future studies should explore feasibility, acceptability, and usability to ensure that models meet the practical requirements of end users in clinical or public health settings.

### Strengths and Limitations

Our review has a number of strengths. We conducted a comprehensive search across 6 databases to capture all AI applications for anogenital dermatoses. We conducted meta-analyses for 5 conditions and explored sources of heterogeneity in mpox studies through meta-regression. In addition, we provided a structured framework of research gaps and practical recommendations for future research and clinical settings.

Our study also has limitations. First, despite our comprehensive search strategy, we may have missed relevant studies in nonindexed journals and commercial AI applications. Second, we modified the QUADAS-2 tool for risk of bias assessment, as it is not specifically designed for AI studies in dermatology. The modification might introduce subjective bias. Third, while we included studies based on target diagnoses relevant to sexual health, many of these AI models were developed using images from both anogenital and nonanogenital body sites. Our findings may not accurately reflect the performance of AI models specifically designed for STIs and anogenital dermatoses. Fourth, we used standard bivariate meta-analysis rather than exact methods, which may be more appropriate for sensitivity and specificity close to 1.^184^ However, our sensitivity analysis using exact methods showed similar results. Fifth, the AI models in our study were limited to identifying visible dermatologic presentations and could not identify other important STI presentations such as discharge, bleeding, and genitourinary symptoms. These AI models would need to be integrated with other approaches to assess wider coverage of symptoms and risk factors for STIs.

## Conclusions

The findings of this systematic review and meta-analysis suggest that while AI shows promising performance metrics for identifying STIs and anogenital dermatoses, significant research gaps exist. Future work should prioritize understudied STIs and differential conditions while improving data quality, conducting external validation, and validating findings in clinical settings. Clear policy guidance and standards are needed to determine how best to implement AI tools for diagnostic purposes and to provide clear performance criteria and frameworks for AI developers, health care practitioners, and clients.

## Notes

Supplement 1. **eTable 1.** Search Strategies and Results **eTable 2**. Summary of Included Studies **eTable 3.** CLEAR Derm Checklist for Included Studies **eTable 4.** Characteristics of Included Studies **eTable 5.** Duplicate Images in Mpox Datasets **eFigure 1.** Quality Assessment by CLEAR Derm Checklist and Modified QUADAS-2 Tool **eFigure 2.** Meta-Analysis Findings (Forest Plots and SROC Graphs)
eReferences

Supplement 2.
Data Sharing Statement
