# Supplementary material for: Use of AI in Identification of Sexually Transmitted Infections and Anogenital Dermatoses: A Systematic Review and Meta-Analysis
Source: JAMA Netw Open. 2025 Oct 3;8(10):e2533512. doi: 10.1001/jamanetworkopen.2025.33512 (PMC12495501; doi:10.1001/jamanetworkopen.2025.33512)
Supplement: Supplement 1. — eTable 1. Search Strategies and Results eTable 2. Summary of Included Studies eTable 3. CLEAR Derm Checklist for Included Studies eTable 4. Characteristics of Included Studies eTable 5. Duplicate Images in Mpox Datasets eFigure 1. Quality Assessment by CLEAR Derm Checklist and Modified QUADAS-2 Tool eFigure 2. Meta-Analysis Findings (Forest Plots and SROC Graphs) eReferences [file jamanetwopen-e2533512-s001.pdf]

Supplementary Online Content

Soe NN, Kusnandar II, Latt PM, et al. Use of AI in identification of sexually transmitted infections and anogenital dermatoses: a systematic review and meta-analysis. *JAMA Netw Open*. 2025;8(10):e2533512.
doi:10.1001/jamanetworkopen.2025.33512

eTable 1. Search Strategies and Results

eTable 2. Summary of Included Studies

eTable 3. CLEAR Derm Checklist for Included Studies

eTable 4. Characteristics of Included Studies

eTable 5. Duplicate Images in Mpox Datasets

eFigure 1. Quality Assessment by CLEAR Derm Checklist and Modified QUADAS-2 Tool

eFigure 2. Meta-Analysis Findings (Forest Plots and SROC Graphs)

eReferences

This supplementary material has been provided by the authors to give readers additional information about their work.

© 2025 Soe NN et al. *JAMA Network Open*.

eTable 1. Search strategies and results

eTable 1a: Ovid Medline (R)

#	Query	Results
1	Neural Networks, Computer/	51,460
2	neural network*.mp.	81,230
3	Algorithms/	310,529
4	Artificial Intelligence/	45,089
5	artificial intelligen*.mp.	52,177
6	Intelligent system*.mp.	839
7	Deep Learning/	18,970
8	deep learning.mp.	38,712
9	few-shot learning.mp.	186
10	Support Vector Machine/	10,289
11	support vector machine*.mp.	22,281
12	Machine Learning/	38,071
13	machine learning.mp.	76,399
14	self supervised learning.mp.	456
15	Diagnosis, Computer-Assisted/	24,313
16	(aided diagnos* or comput* assist* diagnos*).mp.	5,363
17	Decision Support Systems, Clinical/	9,731
18	decision support system*.mp.	14,732
19	Mobile Applications/	12,366
20	digital health.mp.	5,658
21	Smartphone/	9,824
22	Cluster Analysis/	70,549
23	Software Design/	6,153
24	computer vision.mp.	4,796
25	multi-classification.mp.	167
26	pre-train*.mp.	4,672
27	(k-nearest neighbo?r* or knearest neighbo?r*).mp.	4,515
28	contextual transformer.mp.	8
29	yolo.mp.	470
30	swin transformer.mp.	138
31	resnet*.mp.	2,512
32	SHAP.mp.	1,076
33	1 or 2 or 3 or 4 or 5 or 6 or 7 or 8 or 9 or 10 or 11 or 12 or 13 or 14 or 15 or 16 or 17 or 18 or 19 or 20 or 21 or 22 or 23 or 24 or 25 or 26 or 27 or 28 or 29 or 30 or 31 or 32	574,847
34	Skin/	213,520
35	Skin Diseases/	63,663
36	skin*.mp.	849,728
37	Dermatology/	21,690
38	dermato*.mp.	187,212
39	skin manifestation*.mp.	5,953
40	lesion*.mp.	871,933
41	Skin Ulcer/	9,426
42	Dermatitis/	13,651
43	ulcer*.mp.	268,993
44	34 or 35 or 36 or 37 or 38 or 39 or 40 or 41 or 42 or 43	1,937,179
45	33 and 44	26,190

© 2025 Soe NN et al. *JAMA Network Open*.

46	Early Diagnosis/	30,622
47	Diagnosis, Differential/	469,604
48	34 or 35 or 36 or 37 or 38 or 39 or 40 or 41 or 42 or 43 or 46 or 47	2,336,905
49	33 and 48	33,458
50	Skin Diseases, Infectious/	5,768
51	Sexually Transmitted Diseases/	27,888
52	Sexual* transmit*.mp.	49,667
53	Infectious disease*.mp.	129,488
54	Communicable Diseases/	34,640
55	(Genital Disease* or Urogenital Abnormalit* or Urogenital Neoplasm* or urogenital disease*).mp.	35,368
56	Herpes Genitalis/ or Vulvar Diseases/ or Pruritus Vulvae/ or Vulvar Lichen Sclerosus/ or Vulvitis/ or Vulvodynia/	10,739
57	(Vulvar Disease* or Pruritus Vulvae or Vulvar Lichen Sclerosus or Vulvitis).mp.	5,618
58	"Mpox (monkeypox)"/	2,579
59	(Mpox or monkeypox).mp.	3,505
60	Monkeypox virus/	1,315
61	Monkeypox virus.mp.	1,717
62	Syphilis/	23,832
63	Sexually Transmitted Diseases, Bacterial/ or Chancroid/	1,975
64	Syphilis, Cutaneous/	1,130
65	chancre.mp.	793
66	Treponema pallidum/	4,547
67	syphil?i*.mp.	35,447
68	Warts/	5,159
69	Condylomata Acuminata/	5,602
70	wart*.mp.	18,343
71	Molluscum Contagiosum/	1,548
72	molluscum*.mp.	2,156
73	Molluscum contagiosum virus/	323
74	Herpes Simplex/	15,510
75	herpes*.mp.	133,568
76	Herpes Zoster/	11,763
77	Herpes Genitalis/	4,895
78	Herpesvirus 2, Human/	4,746
79	Scabies/	3,943
80	scabie*.mp.	5,102
81	Lichens/	3,424
82	Lichen Planus/	6,168
83	Lichen Sclerosus et Atrophicus/	1,420
84	lichen*.mp.	25,333
85	50 or 51 or 52 or 53 or 54 or 55 or 56 or 57 or 58 or 59 or 60 or 61 or 62 or 63 or 64 or 65 or 66 or 67 or 68 or 69 or 70 or 71 or 72 or 73 or 74 or 75 or 76 or 77 or 78 or 79 or 80 or 81 or 82 or 83 or 84	449,704
86	44 and 85	64,538
87	48 and 85	73,942
88	33 and 86	368
89	33 and 87	489
90	Filtered date: from 1 Jan 2010 to 12 Apr 2024	318

eTable 1b: Embase via Ovid

#	Query	Results
1	artificial neural network/	56,220
2	convolutional neural network/	30,584
3	algorithm/	381,696
4	neural network*.mp.	154,467
5	artificial intelligence/	74,937
6	artificial intelligence.mp.	85,590
7	deep learning/	54,689
8	deep learn*.mp.	77,353
9	support vector machine/	43,271
10	support vector machine*.mp.	50,731
11	automated pattern recognition/	17,677
12	computer analysis/	126,942
13	computer vision.mp.	10,726
14	machine learning/	115,599
15	machine learn*.mp.	160,862
16	image analysis/	242,484
17	image processing/	106,014
18	automated pattern recognition/	17,677
19	automat* diagnos*.mp.	2,878
20	image recogn*.mp.	2,286
21	computer assisted diagnosis/	44,466
22	computer assist* diagnos*.mp.	45,059
23	decision support system/	28,169
24	clinical decision support system/	6,799
25	information system/	43,166
26	clinic* decision support* system*.mp.	8,879
27	aid* diagnos*.mp.	12,716
28	machine learning software/	886
29	medical image management software/	271
30	healthcare software/	588
31	artificial intelligence software/	836
32	imaging software/	75,157
33	open source software/	1,924
34	software validation/	385
35	camera software/	148
36	clinical information management software/	121
37	clinical management support software/	66
38	medical information system software/	130
39	patient health record information system software/	222
40	smartphone/	28,988
41	mobile phone/	23,197
42	mobile application/	23,517
43	k-nearest neighbo?r.mp.	10,541
44	contextual transformer*.mp.	15
45	image* annotat*.mp.	556
46	yolo.mp.	978
47	swin transformer*.mp.	285
48	resnet*.mp.	5,381
49	digital health.mp.	9,818
50	intelligent system*.mp.	1,538
51	pre?train* model*.mp.	482
52	multimodal*.mp.	176,169

© 2025 Soe NN et al. *JAMA Network Open*.

53	multi?classif*.mp.	170
54	skin lesion classif*.mp.	173
55	self supervised learn*.mp.	931
56	few shot learn*.mp.	474
57	1 or 2 or 3 or 4 or 5 or 6 or 7 or 8 or 9 or 10 or 11 or 12 or 13 or 14 or 15 or 16 or 17 or 18 or 19 or 20 or 21 or 22 or 23 or 24 or 25 or 26 or 27 or 28 or 29 or 30 or 31 or 32 or 33 or 34 or 35 or 36 or 37 or 38 or 39 or 40 or 41 or 42 or 43 or 44 or 45 or 46 or 47 or 48 or 49 or 50 or 51 or 52 or 53 or 54 or 55 or 56	1,472,100
58	skin/	257,972
59	skin disease/	98,336
60	skin examination/	14,414
61	skin manifestation/	40,818
62	skin ulcer/	23,255
63	skin manifestation*.mp.	44,267
64	dermatology/	57,244
65	dermato*.mp.	315,166
66	lesion*.mp.	1,576,121
67	ulcer*.mp.	497,138
68	58 or 59 or 60 or 61 or 62 or 63 or 64 or 65 or 66 or 67	2,543,917
69	early diagnosis/	142,270
70	differential diagnosis/	440,138
71	58 or 59 or 60 or 61 or 62 or 63 or 64 or 65 or 66 or 67 or 69 or 70	3,009,777
72	genital skin/	114
73	genital skin.mp.	1,038
74	lumpy skin disease/	496
75	Lumpy skin disease virus/	308
76	skin infection/	30,361
77	viral skin disease/	1,183
78	bacterial skin disease/	3,760
79	sexually transmitted disease/	56,704
80	sexually transmi*.mp.	77,037
81	infectious disease*.mp.	176,710
82	communicable disease/	39,130
83	communicable disease*.mp.	71,151
84	genital system disease/	4,498
85	vulva disease/	3,664
86	penis disease/	5,017
87	herpes simplex/	25,274
88	Herpes simplex virus 1/	23,239
89	Herpes simplex virus 2/	12,890
90	genital herpes/	6,679
91	herpes*.mp.	176,822
92	monkeypox/	4,306
93	monkeypox*.mp.	5,525
94	Monkeypox virus/	2,180
95	mpox*.mp.	1,582
96	syphilis/	46,252
97	secondary syphilis/	2,829
98	cutaneous syphilis/	69
99	latent syphilis/	430
100	syphilis serology/	3,996
101	syphilis*.mp.	57,375
102	ulcus molle/	1,640
103	chancroid.mp.	1,136
104	Treponema pallidum/	9,009
105	treponema pallidum.mp.	11,708

© 2025 Soe NN et al. *JAMA Network Open*.

106	wart/	730
107	Wart virus/	46,407
108	condyloma acuminatum/	9,928
109	wart*.mp.	79,200
110	venereal.mp.	10,386
111	molluscum contagiosum/	3,737
112	Molluscum contagiosum virus/	143
113	molluscum contagiosum.mp.	4,223
114	herpes zoster/	31,473
115	scabies/	7,812
116	scabie*.mp.	9,345
117	"lichen (disease)"/	658
118	lichen planus/	15,464
119	lichen sclerosus et atrophicus/	5,639
120	lichen*.mp.	46,426
121	72 or 73 or 74 or 75 or 76 or 77 or 78 or 79 or 80 or 81 or 82 or 83 or 84 or 85 or 86 or 87 or 88 or 89 or 90 or 91 or 92 or 93 or 94 or 95 or 96 or 97 or 98 or 99 or 100 or 101 or 102 or 103 or 104 or 105 or 106 or 107 or 108 or 109 or 110 or 111 or 112 or 113 or 114 or 115 or 116 or 117 or 118 or 119 or 120	685,112
122	68 and 121	110,877
123	71 and 121	124,518
124	57 and 122	1,901
125	57 and 123	2,315
126	Filtered date: from 1 Jan 2010 to 12 Apr 2024	1729

eTable 1c: IEEE Xplore

#	Query	Results
1	(((All Metadata:skin OR All Metadata:dermato* OR All Metadata:sexually transmit* OR All Metadata:communicable disease* OR All Metadata:herpes OR All Metadata:monkeypox OR All Metadata:mpox OR All Metadata:chancre OR All Metadata:treponema pallidum OR All Metadata:syphilis OR All Metadata:wart* OR All Metadata:condylomata acuminata OR All Metadata:molluscum contagiosum OR All Metadata:scabie* OR All Metadata:lichen*)))<br>AND<br>((All Metadata:artificial neural network OR All Metadata:image analys?s OR All Metadata:image recogni* OR All Metadata:artificial intelligent OR All Metadata:deep learning OR All Metadata:machine learning OR All Metadata:computer assist*))<br>Limited to journals and early access articles<br>Year range from 2010 to 2024 (search result date: 12 Apr 2024)	2,213

eTable 1d: Web of Science

#	Query	Results
1	((((((((((((((((((ALL=("image classif*")) OR ALL=("neural network*")) OR ALL=("computer assist* diagnos?s")) OR ALL=("aid* diagnos?s")) OR ALL=("computer vision")) OR ALL=("machine learning")) OR ALL=("deep learning")) OR ALL=("artificial intelligen*")) OR ALL=("image analys?s")) OR ALL=("image recogni*")) OR ALL=("automated pattern recongi*")) OR ALL=("k?nearest neighbo?r")) OR ALL=("YOLO")) OR ALL=("swin transformer")) OR ALL=("support vector machine")) OR ALL=("resnet*")) OR ALL=("pre?train* model*")) OR ALL=("multimodal*")) OR ALL=("lesion classif*")) OR ALL=("supervised learn*")) OR ALL=("few shot learn*")	1,947,791
2	((((ALL=(skin)) OR ALL=("skin disease*")) OR ALL=("skin manifest*")) OR ALL=("dermato*")) OR ALL=("ulcer*")	1,673,886

© 2025 Soe NN et al. *JAMA Network Open*.

3	((((((((((((((ALL=(lichen*)) OR ALL=(scabie*)) OR ALL=(herpes)) OR ALL=("molluscum contagiosum")) OR ALL=("wart*")) OR ALL=("condylomata acuminata")) OR ALL=("syphil?i*")) OR ALL=(chancre)) OR ALL=("treponema pallidum")) OR ALL=(monkeypox)) OR ALL=(mpox)) OR TS=("*genital")) OR ALL=("genito*")) OR ALL=("communicable disease*")) OR ALL=("sexual health")) OR ALL=("sex* transmit*")	709,597
4	#2 AND #3	70,223
5	#1 AND #4	351
6	#1 AND #4 and 2024 or 2023 or 2022 or 2021 or 2020 or 2019 or 2018 or 2017 or 2016 or 2015 or 2014 or 2013 or 2012 or 2011 or 2010 (Publication Years)	303

eTable 1e: Scopus

#	Query	Results
1	((TITLE-ABS-KEY ("image classif*")) OR (TITLE-ABS-KEY ("artificial neural network*")) OR (TITLE-ABS-KEY ("convolutional neural network*")) OR (TITLE-ABS-KEY ("computer assist* diagnos?s*")) OR (TITLE-ABS-KEY ("aided diagnos?s*")) OR (TITLE-ABS-KEY ("computer vision*")) OR (TITLE-ABS-KEY ("machine learning")) OR (TITLE-ABS-KEY ("deep learning")) OR (TITLE-ABS-KEY ("artificial intelligen*")) OR ((TITLE-ABS-KEY ("image analys?s") OR TITLE-ABS-KEY ("image recogni*"))))<br>AND<br>(((TITLE-ABS-KEY (lichen*)) OR (TITLE-ABS-KEY (scabie*)) OR (TITLE-ABS-KEY (herpes)) OR (TITLE-ABS-KEY (molluscum AND contagiosum)) OR ((TITLE-ABS-KEY (wart*) OR TITLE-ABS-KEY (condylomata AND acuminata))) OR ((TITLE-ABS-KEY (syphil?i*) OR TITLE-ABS-KEY (chancre) OR TITLE-ABS-KEY (treponema AND pallidum))) OR ((TITLE-ABS-KEY (monkeypox) OR TITLE-ABS-KEY (mpox))) OR (TITLE-ABS-KEY (*genital) OR TITLE-ABS-KEY (genito*)) OR (TITLE-ABS-KEY (communicable AND disease*)) OR (TITLE-ABS-KEY (sexual* AND health)) OR (TITLE-ABS-KEY (sexual* AND transmit*))) AND ((TITLE-ABS-KEY (skin*)) OR (TITLE-ABS-KEY (dermato*)) OR (TITLE-ABS-KEY (ulcer* OR lesion* OR lump* OR "skin manifest*"))))<br>AND<br>PUBYEAR > 2009 AND PUBYEAR < 2024	1,384

eTable 1f: CINAHL

#	Query	Results
1	(MH "Image Processing, Computer Assisted/CL/EV") OR (MH "Image Interpretation, Computer Assisted") OR (MH "Support Vector Machine") OR (MH "Neural Networks (Computer)") OR (MH "Machine Learning") OR (MH "Decision Making, Computer Assisted") OR (MH "Data Analysis, Computer Assisted") OR (MH "Decision Support Techniques") OR (MH "Deep Learning") OR (MH "Therapy, Computer Assisted") OR (MH "Diagnosis, Computer Assisted") OR (MH "Decision Support Systems, Clinical") OR (MH "Artificial Intelligence") OR (MH "Decision Support Systems, Management") OR (MH "Computer-Aided Design") OR (MH "Self-Directed Learning") OR "(neural network or deep-learning or machine learning) OR artificial intelligence OR computer vision OR computer assisted decision support OR image analysis OR image processing OR image recognition OR image classification OR k-nearest neighbor OR swin-transformer OR yolo OR support vector machine"	
2	(MH "Skin Manifestations") OR (MH "Skin Diseases") OR (MH "Skin Diseases, Parasitic") OR (MH "Skin Diseases, Metabolic") OR (MH "Skin Diseases, Infectious") OR (MH "Skin Diseases, Genetic") OR (MH "Skin") OR (MH "Skin and Connective Tissue Diseases") OR (MH "Skin Neoplasms") OR "skin OR (dermatology or skin diseases or skin condition) OR skin disorders OR skin infection OR skin manifestations" OR (MH "Skin Pigmentation") OR (MH "Skin Abnormalities") OR (MH "Skin Ulcer") OR (MH "Skin Diseases, Papulosquamous") OR (MH "Skin Diseases, Vascular") OR (MH "Skin Diseases, Vesiculobullous") OR (MH "Skin Maceration") OR (MH	

© 2025 Soe NN et al. *JAMA Network Open*.

	"Staphylococcal Scalded Skin Syndrome") OR (MH "Neoplasms, Adnexal and Skin Appendage") OR (MH "Exanthema")	
3	(MH "Communicable Diseases") OR (MH "Sexually Transmitted Diseases") OR (MH "Sexually Transmitted Diseases, Viral") OR (MH "Sexually Transmitted Diseases, Protozoal") OR (MH "Sexually Transmitted Diseases, Fungal") OR (MH "Sexually Transmitted Diseases, Bacterial") OR (MH "Risk Control: Sexually Transmitted Diseases (STD) (Iowa NOC)") OR (MH "Skin Diseases, Infectious") OR (MH "Post-Infectious Disorders") OR "(*genital or genito*) OR sexual health OR sexually transmitted diseases OR (communicable disease or infectious disease) OR herpes* OR (monkeypox or monkey pox or monkey pox virus) OR (syphilis or treponema pallidum) OR (warts or condyloma acuminatum) OR molluscum contagiosum OR scabies OR lichen" OR (MH "Genital Diseases") OR (MH "Virus Diseases") OR (MH "Genital Diseases, Female") OR (MH "Genital Diseases, Male") OR (MH "Health and Disease") OR (MH "Infection Control") OR (MH "Sexual Health Clinics") OR (MH "Sexual Health Services") OR (MH "Vulvar Diseases") OR (MH "Vaginal Diseases") OR (MH "Penile Diseases") OR (MH "Anus Diseases") OR (MH "Urologic Diseases") OR (MH "Urethral Diseases") OR (MH "Ureteral Diseases") OR (MH "Testicular Diseases") OR (MH "Skin Diseases") OR (MH "Skin Diseases, Vesiculobullous") OR (MH "Disease Transmission") OR (MH "Disease Surveillance") OR (MH "Disease Outbreaks") OR (MH "Skin Diseases, Vascular") OR (MH "Skin Diseases, Parasitic") OR (MH "Skin Diseases, Papulosquamous") OR (MH "Sweat Gland Diseases") OR (MH "Bacterial and Fungal Diseases") OR (MH "Skin and Connective Tissue Diseases") OR (MH "Sexual Intercourse") OR (MH "Sexual Abstinence") OR (MH "Sexual Partners") OR (MH "Sexual Behavior") OR (MH "Warts, Venereal") OR (MH "Herpes Genitalis") OR (MH "Herpes Zoster Vaccine") OR (MH "Syphilis") OR (MH "Genitalia") OR (MH "Warts") OR (MH "Scabies") OR (MH "Human Papillomavirus Viruses") OR (MH "Warts, Plantar") OR (MH "Lichen Planus") OR (MH "Herpes Simplex") OR (MH "Genitalia, Male") OR (MH "Herpes Zoster") OR (MH "Pelvic Floor Disorders")	
4	#2 AND #3	
5	#1 AND #4	
6	Year range from 2010 to 2024 (search result date: 12 Apr 2024)	85

© 2025 Soe NN et al. *JAMA Network Open*.

eTable 2. Summary of included studies

eTable 2a: Syphilis

No	Author, Year	Image Data Source	Type of Lesions included	Sample Size (Target/Total)	Reference Conditions	Study Type	Best Performing AI Algorithm	AUC-ROC	Accuracy	Sensitivity	Specificity	PPV		Modified QUADAS-2		
														P	I	R
1	Allan, 2024 [1]	◆ Multiple (India, Sri Lanka, Singapore, Australia, United States, United Kingdom)	6	190/1,570	1, 2, 4, 5, 15, 99	MD	U-Net + Inception-ResNet-V2	NR	0.944	0.865 (0.712-0.955)	0.985 (0.957 - 0.999)	0.914 (0.822 - 0.999)	RoB	+	+	+
													App	?	+	+

AUC-ROC, Area Under the Curve-Receiver Operating Characteristic; PPV, Positive Predictive Value; P: Population; I: Index Test; R: Reference Standard; RoB: Risk of Bias; App: Applicability; NR: Not Reported; MD: Model Development and Internal Validation; MC: Model Comparison; EV: External Validation; PS-NR: Prospective study (Non-Randomised); CNN: Convolutional Neural Networks;
MSID: Mpox Image Dataset; MSLD: Mpox Skin Lesion Dataset;
◆ *: reviewed by clinician/dermatologist;* ❖ *: confirmed by laboratory and reviewed by clinician/dermatologist*
Reference conditions: (1=Syphilis chancre; 2=Condylomata lata; 3=Syphilis rash; 4=Herpes simplex; 5=Genital warts; 6=Mpox; 7=Molluscum Contagiosum; 8=Tinea Cruris; 9=Lichenoid conditions including lichen sclerosus, lichen planus; 10=Scabies; 11=Folliculitis; 12=Herpes Zoster; 13=Psoriasis; 14=Normal variant/healthy skin; 15=Balanitis; 19=Others)

\+ *Low Concern* ? *Unsure* - *High Concern*

eTable 2b: Herpes Simplex

No	Author, Year	Image Data Source	Type of Lesions included	Sample Size (Target/Total)	Reference Conditions	Study Type	Best Performing AI Algorithm	AUC-ROC	Accuracy	Sensitivity	Specificity	PPV		Modified QUADAS-2		
														P	I	R
1	Gaffoor, 2023 [2]	Kaggle	10	100/1,000	4, 5, 6, 7, 8, 13, 99	MD, MC	SVM	NR	0.100	0.100	NR	0.100	RoB	-	?	-
													App	-	-	-
2	Mehta, 2024 [3]	❖ Private dataset from STI and genital diseases clinic in North India (Jan-2021 to Jun-2022)	8	4/257	4, 5, 6, 7, 8, 9, 10, 99	PS-NR	CNN-based app (DermAId)	NR	0.689	0.300	0.992	NR	RoB	+	+	+
													App	-	+	+
3	Wei, 2018 [4]	NR	3	30/90	99	MD	SVM	NR	0.850	NR	NR	NR	RoB	?	?	-
													App	-	?	-
4	Yadav, 2023 [5]	DermNet	3	810/3,468	99	MD	VGG-16	NR	0.840	NR	NR	NR	RoB	-	?	-
													App	-	-	-
5	Allan, 2024 [1]	◆ Multiple (India, Sri Lanka, Singapore,	6	212/1,570	1, 2, 4, 5, 15, 99	MD		NR	0.944			0.870 (0.772	RoB	+	+	+

© 2025 Soe NN et al. *JAMA Network Open.*

No	Author, Year	Image Data Source	Type of Lesions included	Sample Size (Target/Total)	Reference Conditions	Study Type	Best Performing AI Algorithm	AUC-ROC	Accuracy	Sensitivity	Specificity	PPV		P	I	R
		Australia, United States, United Kingdom)					U-Net + Inception-ResNet-V2			0.930 (0.810-0.985)	0.969 (0.935 - 0.989)	- 0.967)	App	?	+	+
6	Nurul, 2019 [6]	NR	6	NR/72	10, 99	MD	Local Binary Pattern (LBP) + CNN	NR	0.800	NR	NR	NR	RoB	?	-	-
													App	-	-	-
7	Sadik, 2023 [7]	HAM10000 + DermNet	5	5,140/18,692	99	MD, MC	Xception	0.997	0.970	0.990	NR	0.925	RoB	-	?	-
													App	?	?	-

AUC-ROC, Area Under the Curve-Receiver Operating Characteristic; PPV, Positive Predictive Value; P: Population; I: Index Test; R: Reference Standard; RoB: Risk of Bias; App: Applicability; NR: Not Reported; MD: Model Development and Internal Validation; MC: Model Comparison; EV: External Validation; PS-NR: Prospective study (Non-Randomised); CNN: Convolutional Neural Networks;

MSID: Mpox Image Dataset; MSLD: Mpox Skin Lesion Dataset;

◆ *: reviewed by clinician/dermatologist;* ❖ *: confirmed by laboratory and reviewed by clinician/dermatologist*

Reference conditions: (1=Syphilis chancre; 2=Condylomata lata; 3=Syphilis rash; 4=Herpes simplex; 5=Genital warts; 6=Mpox; 7=Molluscum Contagiosum; 8=Tinea Cruris; 9=Lichenoid conditions including lichen sclerosus, lichen planus; 10=Scabies; 11=Folliculitis; 12=Herpes Zoster; 13=Psoriasis; 14=Normal variant/healthy skin; 15=Balanitis; 19=Others)

\+ *Low Concern* ? *Unsure* - *High Concern*

eTable 2c: Genital Warts

No	Author, Year	Image Data Source	Type of Lesions included	Sample Size (Target/Total)	Reference Conditions	Study Type	Best Performing AI Algorithm	AUC-ROC	Accuracy	Sensitivity	Specificity	PPV		Modified QUADAS-2		
														P	I	R
1	Assoc, 2019 [8]	Wise-Geek group	3	NR/240	99	MD	k-nearest neighbor algorithm (k-NN)	NR	0.982	NR	NR	NR	RoB	-	?	-
													App	-	-	-
2	Casuayan, 2020 [9]	◆ Private dataset	7	100/600	14, 99	MD	Support Vector Machine (SVM)	NR	NR	0.906	NR	0.936	RoB	+	?	+
													App	-	-	?
3	Gaffoor, 2023 [2]	Kaggle	10	100/1,000	4, 5, 6, 7, 8, 13, 99	MD, MC	SVM	NR	0.970	0.950	NR	0.100	RoB	-	?	-
													App	-	-	-
4	Mehta, 2024 [3]	❖ Private dataset from STI and genital diseases clinic in North India (Jan-2021 to Jun-2022)	8	95/257	4, 5, 6, 7, 8, 9, 10, 99	MD, PS-NR	CNN-based app (DermAId)	NR	0.689	0.558	1000	NR	RoB	+	+	+
													App	-	+	+
5	Pangti, 2021 [10]	❖ Hellenic Dermatological Atlas + DanDerm	40	NR/15,418	5, 7, 8, 9, 12, 14, 99	MD, PS-NR	DenseNet-161	0.850	NR	0.571 (0.394 – 0.737)	0.997	0.571	RoB	+	+	+
													App	?	+	+

© 2025 Soe NN et al. *JAMA Network Open*.

6	Allan, 2024 [1]	◆ Multiple (India, Sri Lanka, Singapore, Australia, United States, United Kingdom)	6	217/1,570	1, 2, 4, 5, 15, 99	MD	U-Net + Inception-ResNet-V2	NR	0.944	0.956 (0.849 - 0.995)	0.964 (0.927 - 0.985)	0.860 (0.764 - 0.956)	**RoB**	+	+	+
													**App**	?	+	+
7	Zaar, 2020 [11]	❖ Department of Dermatology and Venereology at Sahlgrenska University Hospital in Gothenburg (Apr-2018 to May-2019)	44	NR	44 conditions	PS-NR	NR	NR	Top-1 Accuracy: 0.651 Top-5 Accuracy: 0.914	NR	NR	NR	**RoB**	+	?	+
													**App**	?	+	+
8	Dodia, 2022 [12]	Google + DermNet	5	45/257	99	MD	VGG-16+XGBoost	NR	0.94	1.000	NR	1.000		-	-	-
														-	-	-

AUC-ROC, Area Under the Curve-Receiver Operating Characteristic; PPV, Positive Predictive Value; P: Population; I: Index Test; R: Reference Standard; RoB: Risk of Bias; App: Applicability; NR: Not Reported; MD: Model Development and Internal Validation; MC: Model Comparison; EV: External Validation; PS-NR: Prospective study (Non-Randomised); CNN: Convolutional Neural Networks;
MSID: Mpox Image Dataset; MSLD: Mpox Skin Lesion Dataset;
◆ *: reviewed by clinician/dermatologist;* ❖ *: confirmed by laboratory and reviewed by clinician/dermatologist*
Reference conditions: (1=Syphilis chancre; 2=Condylomata lata; 3=Syphilis rash; 4=Herpes simplex; 5=Genital warts; 6=Mpox; 7=Molluscum Contagiosum; 8=Tinea Cruris; 9=Lichenoid conditions including lichen sclerosus, lichen planus; 10=Scabies; 11=Folliculitis; 12=Herpes Zoster; 13=Psoriasis; 14=Normal variant/healthy skin; 15=Balanitis; 19=Others)

\+ *Low Concern* ? *Unsure* - *High Concern*

eTable 2d: Mpox

**No**	**Author, Year**	**Image Data Source**	**Type of Lesions included**	**Sample Size (Target/Total)**	**Reference Conditions**	**Study Type**	**Best Performing AI Algorithm**	**AUC-ROC**	**Accuracy**	**Sensitivity**	**Specificity**	**PPV**		**Modified QUADAS-2**		
														**P**	**I**	**R**
**1**	Abdelhamid, 2022 [13]	Kaggle	2	279/572	14	MD	GoogLeNet	NR	0.938	0.625	0.998	NR	**RoB**	-	-	-
													**App**	-	-	-
**2**	Agrawal, 2022 [14]	Kaggle	4	222/870	14, 99	MD	EfficientNet-B3	NR	0.930	0.940	NR	0.930	**RoB**	-	?	-
													**App**	-	-	-
**3.1**	Ahsan, 2024 [15]	Arxiv	2	43/76	14	MD	M-VGG16	NR	0.880 ± 0.076	0.880 ± 0.076	NR	0.900 ± 0.069	**RoB**	-	-	-
													**App**	-	-	-
**3.2**	Ahsan, 2024 [15]	Arxiv	2	587/1,754	99	MD	M-VGG16	NR	0.760 ± 0.023	0.770 ± 0.022	NR	0.770 ± 0.022	**RoB**	-	-	-
													**App**	-	-	-

© 2025 Soe NN et al. *JAMA Network Open*.

No	Author, Year	Image Data Source	Type of Lesions included	Sample Size (Target/Total)	Reference Conditions	Study Type	Best Performing AI Algorithm	AUC-ROC	Accuracy	Sensitivity	Specificity	PPV		Modified QUADAS-2		
														**P**	**I**	**R**
**3.3**	Ahsan, 2024 [15]	Arxiv	4	264/656	14, 99	MD	M-Resnet50	NR	0.890 ± 0.021	0.840 ± 0.025	NR	0.830 ± 0.026	**RoB**	-	-	-
													**App**	-	-	-
**3.4**	Ahsan, 2024 [15]	Arxiv	2	866/2,524	14	MD	M-VGG16	NR	0.770 ± 0.036	0.740 ± 0.038	NR	0.820 ± 0.026	**RoB**	-	-	-
													**App**	-	-	-
**4.1**	Ahsan, 2023 [16]	Kaggle	2	43/76	14	MD	VGG-19	NR	0.930	0.940	0.850	0.940	**RoB**	-	-	-
													**App**	-	-	-
**4.2**	Ahsan, 2023 [16]	Kaggle	2	587/818	14	MD	MobileNet-V2	NR	0.990	0.990	0.970	0.990	**RoB**	-	-	-
													**App**	-	-	-
**5.1**	Ahsan, 2023 [17]	Kaggle	2	43/76	14	MD	Extreme Inception (Xception)	NR	0.940 ± 0.759	0.940 ± 0.054	NR	0.940 ± 0.054	**RoB**	-	?	-
													**App**	-	-	-
**5.2**	Ahsan, 2023 [17]	Kaggle	2	587/1,754	14	MD	Extreme Inception (Xception)	NR	0.800 ± 0.021	0.80 ± 0.021	NR	0.800 ± 0.021	**RoB**	-	?	-
													**App**	-	-	-
**5.3**	Ahsan, 2023 [17]	Kaggle	4	264/659	14, 99	MD	ResNet-101	NR	0.990 ± 0.800	0.990 ± 0.008	NR	0.990 ± 0.008	**RoB**	-	?	-
													**App**	-	-	-
**6**	Alcalá-Rmz, 2023 [18]	Kaggle	4	1168/2,607	99	MD	MiniGoogLeNet	0.740	0.970	NR	NR	NR	**RoB**	-	?	-
													**App**	-	-	-
**7**	Alharbi, 2023 [19]	Kaggle	2	NR/770	99	MD	GoogLeNet + Decision Tree Classifier	NR	0.944	0.950	0.610	0.890	**RoB**	-	-	-
													**App**	-	-	-
**8**	Alhasson, 2023 [20]	MSID	4	NR/770	14, 99	MD	MobileNet-V2	0.990	0.990	1000	NR	0.950	**RoB**	-	?	-
													**App**	-	-	-
**9.1**	Almufareh, 2023 [21]	MSID	4	279/477	14, 99	MD	MobileNet-V2	0.981	0.960	1000	0.931	NR	**RoB**	-	-	-
													**App**	-	-	-
**9.2**	Almufareh, 2023 [21]	MSLD	4	102/228	14, 99	MD	Inception-V3	0.986	0.933	0.880	1000	NR	**RoB**	-	-	-
													**App**	-	-	-
**10.1**	Almutairi, 2022 [22]	MSID	2	NR/770	99	MD	VGG-19	0.967	0.977	0.952	0.980	0.951	**RoB**	-	?	-
													**App**	-	-	-
**10.2**	Almutairi, 2022 [22]	MSLD	2	NR/659	99	MD	VGG-16	0.967	0.975	0.949	0.945	0.950	**RoB**	-	?	-
													**App**	-	-	-
**11**	Aloraini, 2024 [23]	MSLD	3	NR/3,192	99	MD	Vision Transformer	NR	0.947	0.950	NR	0.950	**RoB**	-	?	-
													**App**	-	-	-
**12**		◆ Google	4	50/200	14, 99	MD	VGG-16	NR	0.960	0.910	NR	0.920	**RoB**	-	?	+

© 2025 Soe NN et al. *JAMA Network Open*.

No	Author, Year	Image Data Source	Type of Lesions included	Sample Size (Target/Total)	Reference Conditions	Study Type	Best Performing AI Algorithm	AUC-ROC	Accuracy	Sensitivity	Specificity	PPV		Modified QUADAS-2		
														P	I	R
	Alrusaini, 2023[24]												App	-	-	?
13	Altun, 2023 [25]	Google + MSLD	5	1200/1,742	99	MD	MobileNet-V3s	0.99	0.968	0.962	NR	NR	RoB	-	?	-
													App	-	-	-
14	Amin, 2023 [26]	MSID	4	NR/659	14, 99	MD	Javeria Network (J-Net) + KNN	NR	0.999	1000	NR	1000	RoB	-	?	-
													App	-	-	-
15	Arshed, 2024 [27]	MSID	4	279/770	14, 99	MD	Vision Transformer (ViT)	NR	0.930	0.930	NR	0.930	RoB	-	?	-
													App	-	-	-
16.1	Asif, 2024 [28]	MSLD	2	1428/3,192	99	MD	Esemble Model	1	1.000	1.000	NR	1.000	RoB	-	+	-
													App	?	-	-
16.2	Asif, 2024 [28]	MSID	4	279/771	14, 99	MD	Esemble Model ((MobileNetV1 + MobileNet-V2 + DenseNet-121 + DenseNet-169 + Inception-V3)	0.998	0.942	0.942	NR	0.942	RoB	-	+	-
													App	?	-	-
17	Asif, 2023 [29]	MSLD	4	1428/3,192	99	MD	Esemble Model ((MobileNetV1 + MobileNet-V2 + DenseNet-121 + DenseNet-169 + Inception-V3)	NR	0.978	1000	NR	0.952	RoB	-	?	-
													App	-	-	-
18.1	Attallah, 2023 [30]	MSID	4	102/228	14, 99	MD	Ensemble (Xception + ResNet-101 + ResNet-50) for MSID, (Xception + ResNet-101 + ResNet-18)	0.950	0.971	0.957	0.983	0.982	RoB	?	+	-
													App	-	-	-
18.2	Attallah, 2023 [30]	MSLD	4	102/228	14, 99	MD	Ensemble (Xception + ResNet-101 + ResNet-50) for MSID, (Xception + ResNet-101 + ResNet-18)	0.980	0.987	0.990	0.984	0.981	RoB	-	+	-
													App	-	-	-
19	Bala, 2023 [31]	MSID	4	279/770	14, 99	MD	Modified DenseNet-201	0.985	0.919	0.919	NR	0.919	RoB	-	+	-
													App	-	-	-

© 2025 Soe NN et al. *JAMA Network Open*.

No	Author, Year	Image Data Source	Type of Lesions included	Sample Size (Target/Total)	Reference Conditions	Study Type	Best Performing AI Algorithm	AUC-ROC	Accuracy	Sensitivity	Specificity	PPV		Modified QUADAS-2		
														P	I	R
20	Bansal, 2023 [32]	Different sources	6	412/1,503	14, 99	MD	Ensemble (Majority voting among VGG-16, Inception-V3, MobileNet-V2)	NR	0.900	0.850	NR	0.950	RoB	-	+	-
													App	-	-	-
21	Bogar, 2023 [33]	MSLD	3	102/456	99	MD	EfficientNet-B3	NR	0.980	0.961	-	0.994	RoB	-	?	-
													App	-	-	-
22.1	Campana, 2024 [34]	MCSI	4	100/400	14, 99	MD	MobileNet-V3 (Small)	NR	0.930 ± 0.410	0.877 ± 0.670	0.780 ± 0.123	NR	RoB	?	+	-
													App	-	-	-
22.2	Campana, 2024 [34]	MCSI	4	100/400	14, 99	MD	MobileNet-V3 (large)	NR	0.882 ± 0.570	0.881 ± 0.550	0.960 ± 0.019	NR	RoB	?	+	-
													App	-	-	-
23	Chandrahaas, 2023 [35]	Google	4	300/801	12, 99	MD, MC	AlexNet	NR	0.836	NR	NR	NR	RoB	-	-	-
													App	-	-	-
24.1	Chauhan, 2023 [36]	MSLD	2	43/76	99	MD, MC	ResNet-50	NR	0.889	0.750	0.880	NR	RoB	-	?	-
													App	-	-	-
24.2	Chauhan, 2023 [36]	Monkeypox-2022	2	1,168/2,607	99	MD, MC	ResNet-101	NR	0.936	0.890	1000	NR	RoB	-	?	-
													App	-	-	-
25	Chen, 2023 [37]	Monkeypox-2022	3	NR/447	99	MD	Self-supervised cross-domain adaption (SCA)	NR	0.876 ± 0.004	NR	NR	NR	RoB	-	?	-
													App	-	-	-
26	Chen, 2023 [38]	Kaggle	6	160/1,027	14, 99	MD, MC	LaCTResNet-56	0.944	0.919	0.9	NR	0.947	RoB	-	+	-
													App	-	-	-
27	Chintamaneni, 2024 [39]	MSID	4	NR/1,429	14, 99	MD, MC	Custom Deep Learning Neural Network (CDCNN) - MonkeypoxNet	NR	0.991	0.987	0.991	0.994	RoB	-	?	-
													App	-	-	-
28	Ciran, 2023 [40]	Kaggle	4	279/770	14, 99	MD	DenseNet-201+ResNet-101+DarkNet-53+SMOTE+BPSO+BGWO	NR	0.995	0.990	NR	0.99	RoB	-	?	-
													App	-	-	-
29	Dahiya, 2023 [41]	Roboflow	3	NR/971	14, 99	MD	YOLOv5	NR	0.983	0.928	NR	0.991	RoB	-	+	-
													App	-	-	-
30	Dan, 2022 [42]	◆ Monkeypox-2022 + MSLD + MSID	4	NR/2,090	8, 14, 99	MD, MC	LSANet (Lesion-Specific Attention Network)	0.987 ± 0.003	0.935 ± 0.001	0.916 ± 0.001	0.977 ± 0.004	0.940 ± 0.001	RoB	-	+	+
													App	-	-	?

© 2025 Soe NN et al. *JAMA Network Open*.

No	Author, Year	Image Data Source	Type of Lesions included	Sample Size (Target/Total)	Reference Conditions	Study Type	Best Performing AI Algorithm	AUC-ROC	Accuracy	Sensitivit y	Specificit y	PPV		Modified QUADAS-2		
														P	I	R
31	Dwivedi, 2022 [43]	Kaggle	6	160/847	14, 99	MD, MC	EfficientNet-B3	NR	0.870	0.870	NR	0.920	RoB	-	?	-
													App	-	-	-
32	Eliwa, 2023 [44]	Kaggle		-	99	MD	CNN optimized by Grey Wolf Optimizer (GWO)	0.927	0.953	0.982	NR	0.956	RoB	-	-	-
													App	-	-	-
33	Ezenkwu, 2023 [45]	MSLD	3	102/228	99	MD, MC	Inception-V3	NR	0.942 ± 0.024	0.964 ± 0.020	NR	0.935 ± 0.039	RoB	-	?	-
													App	-	-	-
34	Fisranda, 2023 [46]	MSID	4	56/770	14, 99	MD, MC	YOLOv5n	NR	0.982	NR	NR	NR	RoB	-	-	-
													App	-	-	-
35	Gairola, 2022 [47]	Kaggle	3	1,000/2,187	99	MD, MC	GoogLeNet +VGG and RF classifier	0.993	0.956	1000	NR	0.909	RoB	-	?	-
													App	-	-	-
36	Gupta, 2023 [48]	MSLD	2	587/1,905	99	MD	ResNet-50	NR	0.988	0.988	NR	0.988	RoB	-	?	-
													App	-	-	-
37	Gupta, 2023 [49]	Kaggle (Augmented)	6	422/825	99	MD, MC	EfficientNet-B3	NR	0.975	0.970	NR	0.970	RoB	-	?	-
													App	-	-	-
38	Haque, 2022 [50]	MSLD	3	228/2,142	99	MD, MC	Xception-CBAM-Dense	NR	0.839	0.891	NR	0.907	RoB	-	?	-
													App	-	-	-
39.1	Haque, 2023 [51]	MSID	4	279/770	14, 99	MD, MC	Ensemble model with majority voting	NR	0.987	1000	NR	1000	RoB	-	?	-
													App	-	-	-
39.2	Haque, 2023 [51]	Monkeypox-2022	4	43/171	14, 99	MD, MC	Ensemble model with majority voting	NR	0.894	0.800	NR	1000	RoB	-	?	-
													App	-	-	-
39.3	Harikiran, 2023 [51]	MSLD	4	387/2,337	99	MD	Modified ResNet-50	NR	0.947	0.883	0.883	0.913	RoB	-	?	-
													App	-	-	-
39.4	Harikiran, 2023 [51]	MSLD	4	340/680	14, 99	EV	Modified ResNet-50	NR	0.765	0.744	0.749	0.700	RoB	-	?	-
													App	-	-	-
40	Haripriya, 2024 [52]	MSID	4	279/770	14, 99	MD, MC	Gradient Boosting with Wavelets + GLCM features	NR	0.844	0.835	NR	0.845	RoB	-	?	-
													App	-	-	-
41	Hossen, 2023 [53]	MSID	4	279/770	14, 99	MD	EfficientNet-B0	NR	0.965	0.965	NR	0.966	RoB	-	+	-
													App	-	-	-
42	Huong, 2023 [54]	MSLD + MSID + Monkeypox-2022	4	264/659	14, 99	MD. MC	MobileNet + Logistic Regression	NR	0.970	0.980	NR	0.990	RoB	-	-	-
													App	-	-	-

© 2025 Soe NN et al. *JAMA Network Open*.

No	Author, Year	Image Data Source	Type of Lesions included	Sample Size (Target/Total)	Reference Conditions	Study Type	Best Performing AI Algorithm	AUC-ROC	Accuracy	Sensitivity	Specificity	PPV		Modified QUADAS-2		
														P	I	R
43	Hussain, 2023 [55]	Google	2	NR/1,139	14	MD, MC	Ehanced CNN (Ensemble ResNet50, Inception-V3, VGG-16, Modified VGG16)	NR	0.991	0.992	NR	0.991	RoB	-	?	-
													App	-	-	-
44	Ieee, 2023 [56]	Google	2	102/228	99	MD, MC	DarkNet-19	0.995	0.967	0.980	0.958	0.950	RoB	-	?	-
													App	-	-	-
45	Ieee, 2022 [57]	MSLD	4	1,162/2,142	99	MD, MC	EfficientNet-B7	NR	0.900	0.950	NR	0.830	RoB	-	-	-
													App	-	-	-
46	Ieee, 2022 [58]	MSID	4	279/770	14, 99	MD, MC	MobileNet-V2	NR	0.914	0.868	NR	0.905	RoB	-	?	-
													App	-	-	-
47	Ieee, 2023 [59]	MSID	4	279/770	14, 99	MD, MC	ResNet50-based features with GWO	NR	0.968	0.980	NR	0.953	RoB	-	?	-
													App	-	-	-
48.1	Islam, 2022 [60]	◆ Web-scraped	6	117/804	14, 99	MD	ShuffleNet-V2	NR	0.790	0.580	NR	0.790	RoB	-	+	+
													App	-	-	?
48.2	Islam, 2022 [60]	◆ Web-scraped	6	117/804	14, 99	MD, MC	ShuffleNet-V2	NR	0.790	0.580	NR	0.790	RoB	-	+	+
													App	-	-	?
49	Jahan, 2023 [61]	MSLD	4	102/228	99	MD, MC	Proposed model (FL + supervised CNN)	0.930	0.900	NR	NR	NR	RoB	-	?	-
													App	-	-	-
50	Jaradat, 2023 [62]	Monkeypox-2022 + MSLD	4	45/117	14, 99	MD, MC	MobileNet-V2	NR	0.982	0.960	NR	0.990	RoB	-	?	-
													App	-	-	-
51	Kakulapati, 2023 [63]	Web-scraped	NR	NR/150	99	MD, MC	UNET-VGG-16	NR	NR	NR	NR	NR	RoB	-	?	-
													App	-	-	-
52	Kaushal, 2023 [64]	Kaggle	4	279/770	14, 99	MD	VGG-16	NR	0.920	NR	NR	NR	RoB	-	-	-
													App	-	-	-
53	Khafaga, 2022 [65]	MSID	4	279/293	14, 99	MD	Al-Brunei Earth radius optimisation-based stochastic fractal search (BERSFS-CNN)	NR	0.988	0.857	0.992	0.760	RoB	-	-	-
													App	-	-	-
54		Web-scraped	4	752/3,073	14, 99	MD		NR	0.976	0.976	NR	0.976	RoB	-	+	-

© 2025 Soe NN et al. *JAMA Network Open*.

No	Author, Year	Image Data Source	Type of Lesions included	Sample Size (Target/Total)	Reference Conditions	Study Type	Best Performing AI Algorithm	AUC-ROC	Accuracy	Sensitivity	Specificity	PPV		Modified QUADAS-2		
														P	I	R
	Khan, 2024 [66]						DenseNet-201 + Logistic Regression (DNLR-NET)						App	-	-	-
55	Kottath, 2023 [67]	Kaggle	-	1,116/2,288	99	MD, MC	EfficientNet-B0	0.956	0.950	0.923	0.975	0.972	RoB	-	+	-
													App	-	-	-
56	Krishnan, 2023 [68]	MSLD	4	102/228	14, 99	MD	2D CNN-based autoencoder	NR	0.980	NR	NR	NR	RoB	-	?	-
													App	-	-	-
57	Krishnan, 2024 [69]	MSLD	4	NR/2562	99	MD	CNN with RootSIFT	NR	0.997	NR	NR	NR	RoB	-	?	-
													App	-	-	-
58	Krishnan, 2023 [70]	MSLD	4	102/228	14, 99	MD	Hyper-parameter tuned transferable CNN (HPT-TCNN)	NR	0.936	0.936	0.936	0.936	RoB	-	-	-
													App	-	-	-
59	Kumar, 2023 [71]	◆ Google + ArXiv	4	143/492	14, 99	MD, MC	XRS-YOLO v3-tiny	NR	0.890	0.970	NR	0.960	RoB	-	+	+
													App	-	-	?
60	Kumar, 2022 [72]	MSLD	NR	102/228	99	MD	Vgg16Net + Naive Bayes	NR	0.911	0.950	NR	0.905	RoB	-	-	-
													App	-	-	-
61	Kundu, 2024 [73]	MSID + arXiv	4	381/886	14, 99	MD, MC	ViT-B32	0.990	0.979	0.960	NR	0.980	RoB	-	?	-
													App	-	-	-
62	Kundu, 2022 [74]	Kaggle	4	102/228	99	MD, MC	ViT	NR	0.930	0.910	NR	0.930	RoB	-	?	-
													App	-	-	-
63	Kundu, 2023 [75]	MSLD	4	102/228	99	MD	EfficientNetV2-B3 + ResNet152-V2	NR	0.965	0.965	NR	0.966	RoB	-	+	-
													App	-	-	-
64	Lakshmi, 2023 [76]	Kaggle + Web-scraped	4	432/835	14, 99	MD, MC	ResNet-101	0.980	0.940	0.940	0.940	0.940	RoB	-	+	-
													App	-	-	-
65	Liu, 2023 [77]	MSLD	4	102/228	99	MD	VGG-16 Siamese network	0.906	0.882	0.868	NR	0.904	RoB	-	?	-
													App	-	-	-
66	Liu, 2023 [78]	MSLD	NR	102/228	99	MD	Multilinear bilinear pooling of EfficientNet and DenseNet	NR	0.945 ± 0.027	0.945 ± 0.027	NR	0.946 ± 0.027	RoB	-	?	-
													App	-	-	-
67	Madhu, 2023 [79]	MSID	4	NR/770	14, 99	MD	Three Dimensional	NR	0.969	0.982	NR	0.978	RoB	-	?	-
													App	-	-	-

© 2025 Soe NN et al. *JAMA Network Open*.

No	Author, Year	Image Data Source	Type of Lesions included	Sample Size (Target/Total)	Reference Conditions	Study Type	Best Performing AI Algorithm	AUC-ROC	Accuracy	Sensitivity	Specificity	PPV		Modified QUADAS-2		
														P	I	R
							DenseUNet with LSTM									
68	Magboo, 2023 [80]	MSID	4	102/228	99	MD, MC	Inception-V3	NR	0.956	0.950	0.960	0.950	RoB	-	+	-
													App	-	-	-
69	Meena, 2024 [81]	MSLD	4	102/228	99	MD, MC	InceptionV3	0.976	0.980	0.980	NR	0.980	RoB	-	+	-
													App	-	-	-
70	Muduli, 2023 [82]	MSLD	NR	102/288	14	MD, MC	VGG-16 + Xception + SVM	1000	0.971	0.938	1000	NR	RoB	-	?	-
													App	-	-	-
71	Munoz-Saavedra, 2023 [83]	Web-scraped (such as CSC or WHO)	3	100 /300	14, 99	MD, MC	Ensemble of ResNet-50, EfficientNet-B0, and MobileNet-V2	NR	0.983	1000	0.975	0.952	RoB	-	+	-
													App	?	-	-
72	Nayak, 2023 [84]	MSID	4	279/770	14, 99	MD, MC	ResNet-18	NR	0.911	0.904	NR	0.947	RoB	-	+	-
													App	-	-	-
73	Nayak, 2023 [85]	MSID	4	102/228	99	MD, MC	ResNet-18	NR	0.995	0.989	1000	1000	RoB	-	+	-
													App	-	-	-
74	Nazmee, 2023 [86]	MSID	4	1,116/1,172	14, 99	MD, MC	MobileNet	1000	0.970	0.968	NR	0.960	RoB	-	+	-
													App	-	-	-
75	Ozaltin, 2023 [87]	MSLD	4	102/228.	99	MD	MobileNet-V2	0.999	0.986	0.981	0.990	0.987	RoB	-	+	-
													App	-	-	-
76	Oztel, 2023 [88]	MSLD + PAD-UFES-20	10	102/2,298	99	MD, MC	ResNet-18	NR	0.743	0.714	NR	0.768	RoB	-	?	-
													App	?	-	-
77	Pabbi, 2023 [89]	MSID	4	279/770	14, 99	MD	VGG-16 +Extra Trees Classifier	NR	0.950	0.950	NR	0.950	RoB	-	?	-
													App	-	-	-
78	Pal, 2023 [90]	Kaggle	4	102/228	14, 99	MD	Inception-V3	0.941	0.966	0.960	NR	0.970	RoB	-	?	-
													App	-	-	-
79	Pal, 2023 [91]	MSLD + Monkeypox-2022	NR	NR/9,900 (augmented)	99	MD, MC	Inception-V3	NR	0.950	NR	NR	NR	RoB	-	?	-
													App	-	-	-
80	Pasha, 2023 [92]	Web-scraped	NR	NR	99	MD, MC	Multilayer-CNN (ML-CNN)	NR	0.991	0.991	NR	0.991	RoB	-	-	-
													App	-	-	-
81	Pramanik, 2023 [93]	MSID	4	NR/926	14, 99	MD, MC	InceptionNet-V3	NR	0.936	0.839	0.990	0.979	RoB	-	-	-
													App	-	-	-

© 2025 Soe NN et al. *JAMA Network Open*.

No	Author, Year	Image Data Source	Type of Lesions included	Sample Size (Target/Total)	Reference Conditions	Study Type	Best Performing AI Algorithm	AUC-ROC	Accuracy	Sensitivity	Specificity	PPV		Modified QUADAS-2		
														P	I	R
82	Pramanik, 2023 [94]	MSLD	4	102/228	99	MD, MC	Ensemble of InceptionV3, Xception and DenseNet169	0.990	0.934	0.968	NR	0.889	RoB	-	+	-
													App	-	-	-
83	Prasher, 2023 [95]	MSLD	4	102/228	99	MD, MC	CNN with SGD optimizer	NR	0.934	NR	NR	NR	RoB	-	-	-
													App	-	-	-
84	Raha, 2024 [96]	MSID + DermNet	8	279/1,285	7, 10, 14, 99	MD, MC	Proposed attention-based MobileNetV2	NR	0.923	0.894	0.989	0.905	RoB	-	+	-
													App	-	?	-
85	Rai, 2023 [97]	MSLD	4	279/720	14, 99	MD, MC	Proposed MobileNetV2 with GAP + Dense 160	NR	0.885	0.874	NR	0.863	RoB	-	+	-
													App	-	-	-
86	Rao, 2023 [98]	Web-scraped	4	-	14, 99	MD, MC	Proposed machine learning CNN	NR	0.991	0.991	NR	0.991	RoB	-	?	-
													App	-	-	-
87.1	Ren, 2023 [99]	MSLD	4	1,428/3,192 (Augmented)	99	MD, MC	DenseNet-201	NR	0.989	NR	NR	NR	RoB	-	-	-
													App	-	-	-
87.2	Ren, 2023 [99]	Monkeypox-2022	4	301/1,468	14, 99	MD, MC	DenseNet-201	NR	1.000	NR	NR	NR	RoB	-	-	-
													App	-	-	-
87.3	Ren, 2023 [99]	Online (https://www.heywhale.com/mw/dataset/62eb75d6fef0903951b1f199/content)	6	5,733/39,396	14, 99	MD, MC	DenseNet-201	NR	0.999	NR	NR	NR	RoB	-	-	-
													App	-	-	-
88	Sahin, 2022 [100]	MSLD	4	102/228	99	MD, MC	MobileNet-V2	NR	0.911	0.900	NR	0.900	RoB	-	+	-
													App	-	-	-
89	Sathwik, 2023 [101]	MSID + DermNet	>4	102/228	99	MD, MC	VGG-19, (Resnet-50 got same performance)	NR	0.920	0.890	NR	0.930	RoB	-	-	-
													App	-	-	-
90	Shah, 2022 [102]	MSLD + MSID + Monkeypox-2022	4	9,068/20,826 (Augmented)	99	MD, MC	MobileNet-V2	NR	0.988	NR	NR	NR	RoB	-	-	-
													App	-	-	-
91	Sharma, 2023 [103]	MSID	4	102/228	99	MD	Proposed CNN model + Adam optimizer	NR	0.922	0.910	NR	0.940	RoB	-	?	-
													App	-	-	-
92	Sharma, 2023 [104]	MSLD	4	630/1,528 (Augmented)	14,99	MD	ResNet-18	NR	0.846	0.846	NR	0.847	RoB	-	?	-
													App	-	-	-
93.1		Monkeypox-2022	4	164/361	14, 99	MD		0.833					RoB	-	?	-

© 2025 Soe NN et al. *JAMA Network Open*.

No	Author, Year	Image Data Source	Type of Lesions included	Sample Size (Target/Total)	Reference Conditions	Study Type	Best Performing AI Algorithm	AUC-ROC	Accuracy	Sensitivity	Specificity	PPV		Modified QUADAS-2		
														P	I	R
	Sharma, 2023 [105]						Modified VGG-16		0.830± 0.085	0.660 ± 0.120	0.89 ± 0.008	0.880 ± 0.072	App	-	-	-
93.2	Sharma, 2023 [105]	Monkeypox-2022	4	164/361	14, 99	MD	VGG-16	0.748	0.780 ± 0.022	0.650 ± 0.280	0.83 ± 0.019	0.750 ± 0.023	RoB	-	?	-
													App	-	-	-
94	Singh, 2022 [106]	MSID	2	NR	14	MD, MC	GoogLeNet	NR	0.883	0.865	NR	0.863	RoB	-	-	-
													App	-	-	-
95	Soe, 2023 [107]	❖ Melbourne Sexual Health Centre (2010-2022) + Kaggle + Web-scraped	9	635/2,200	1, 2, 3, 4, 5, 7, 8, 12	MD, MC, EV	DenseNet-121	0.982 ± 0.002	0.951 ± 0.003	0.940 ± 0.006	NR	0.956 ± 0.000	RoB	+	+	+
													App	?	+	+
96	Sorayaie, 2023 [108]	Kaggle	4	43/171	14, 99	MD, MC	DenseNet-121	0.943	0.976	0.918	0.985	0.900	RoB	-	+	-
													App	-	-	-
97	Supanich, 2023 [109]	MSLD + random image	4	102/1,645	99	MD, MC	EfficientNet-B4	NR	0.952	0.950	NR	0.950	RoB	-	?	-
													App	-	-	-
98	Surati, 2023 [110]	MSLD +MSID + Monkeypox-2022	3	755/2,323	99	MD, MC	SENet + Inception-V3	NR	0.980	0.980	NR	0.981	RoB	-	?	-
													App	-	-	-
199	Taruno, 2023 [111]	Web-scraped	2	102/228	99	MD, MC	EfficientNet-B0	NR	0.851	0.785	0.918	NR	RoB	-	?	-
													App	-	-	-
100	Thieme, 2023 [112]	❖ Danderm + DermIS + AD + DermNet + DermNet NZ + PAD-UFES-20 + Fitzpatrick 17k + Web-scraped + Prospective cohort of the Standford University Medical Center	NR	676/139,198	99	MD, EV, PS-NR	MPXV-CNN	0.967 ± 0.003	NR	0.910	0.898	NR	RoB	+	+	+
													App	?	+	+
101	Thorat, 2024 [113]	MSLD	2	102/228	99	MD, MC	ResNet50-V2	0.986	0.987	1000	0.977	0.973	RoB	-	+	-
													App	-	-	-
102	Tiwari, 2023 [114]	Kaggle	4	43/161	14, 99	MD, MC	MPox-DenseConvNet using HSV color channel	0.990	0.990	1000	NR	0.990	RoB	-	+	-
													App	-	-	-
103		-	2	43/70	99		DenseNet-121	NR	0.937	0.875	NR	1000	RoB	-	?	-

© 2025 Soe NN et al. *JAMA Network Open*.

No	Author, Year	Image Data Source	Type of Lesions included	Sample Size (Target/Total)	Reference Conditions	Study Type	Best Performing AI Algorithm	AUC-ROC	Accuracy	Sensitivity	Specificity	PPV		Modified QUADAS-2		
														P	I	R
	Torky, 2022 [115]					MD, MC							App	-	-	-
104	Ural, 2023 [116]	Kaggle + Web-scraped	4	100/400	14, 99	MD, MC	VGG-16	NR	0.750 ± 0.071	1000	0.700 ± 0.140	NR	RoB	-	?	+
													App	-	-	?
105	Uysal, 2023 [117]	MSLD	4	279/770	14, 99	MD, MC	Proposed CNN-LSTM hybrid model	0.934	0.87 ± 0.035	0.870	NR	0.930	RoB	-	+	-
													App	-	-	-
106	Uzun, 2023 [118]	Kaggle + Web-scraped	2	102/342	99	MD, MC	Customised CNN model	NR	0.990	1000	NR	0.980	RoB	-	+	-
													App	-	-	-
107	Vajpayee, 2023 [119]	MSID	4	285/782	14, 99	MD, MC	EfficientNet-V2L	0.977	0.925	0.921	NR	0.925	RoB	-	?	-
													App	-	-	-
108	Yadav, 2024 [120]	MSLD + Web-scraped	4	299/1,433	99	MD	ResNet-50 + XGBoost	NR	0.974	0.970	NR	0.980	RoB	-	?	-
													App	-	-	-
109	Yasmin, 2023 [121]	MSLD	2	1,428/3,192	99	MD, MC	Proposed PoxNet22 (based off InceptionV3) + Adam optimiser	NR	0.990	0.990	NR	0.990	RoB	-	?	-
													App	-	-	-
110	Zi, 2022 [122]	MSLD	2	Mpox: 102/228	99	MD, MC	Custom CNN	NR	0.908 ± 0.011	0.910 ± 0.010	NR	0.900 ± 0.020	RoB	-	?	-
													App	-	-	-

AUC-ROC, Area Under the Curve-Receiver Operating Characteristic; PPV, Positive Predictive Value; P: Population; I: Index Test; R: Reference Standard; RoB: Risk of Bias; App: Applicability; NR: Not Reported; MD: Model Development and Internal Validation; MC: Model Comparison; EV: External Validation; PS-NR: Prospective study (Non-Randomised); CNN: Convolutional Neural Networks;
MSID: Mpox Image Dataset; MSLD: Mpox Skin Lesion Dataset;
◆ *: reviewed by clinician/dermatologist;* ❖ *: confirmed by laboratory and reviewed by clinician/dermatologist*
Reference conditions: (1=Syphilis chancre; 2=Condylomata lata; 3=Syphilis rash; 4=Herpes simplex; 5=Genital warts; 6=Mpox; 7=Molluscum Contagiosum; 8=Tinea Cruris; 9=Lichenoid conditions including lichen sclerosus, lichen planus; 10=Scabies; 11=Folliculitis; 12=Herpes Zoster; 13=Psoriasis; 14=Normal variant/healthy skin; 15=Balanitis; 19=Others)

\+ *Low Concern* ? *Unsure* - *High Concern*

© 2025 Soe NN et al. *JAMA Network Open*.

eTable 2e: Molluscum Contagiosum

No	Author, Year	Image Data Source	Type of Lesions included	Sample Size (Target/Total)	Reference Conditions	Study Type	Best Performing AI Algorithm	AUC-ROC	Accuracy	Sensitivity	Specificity	PPV		Modified QUADAS-2		
														P	I	R
1	Danpakdee, 2017 [123]	Web-scrapped	4	25/100	8, 12, 99	MD,MC	Multi-layer perceptron with Back propagation (MLP)	NR	NR	NR	NR	NR	RoB	-	-	-
													App	-	-	-
2	Gaffoor, 2023 [2]	Kaggle	10	100/1,000	4, 5, 6, 7, 8, 13, 99	MD, MC	SVM	NR	0.100	0.100	NR	0.100	RoB	-	?	-
													App	-	-	-
3	Mehta, 2024 [3]	❖ Private dataset from STI and genital diseases clinic in North India (Jan-2021 to Jun-2022)	8	14/257	4, 5, 6, 7, 8, 9, 10, 99	PS-NR	CNN-based app (DermAId)	NR	0.689	0.643	0.996	NR	RoB	+	+	+
													App	-	+	+
4	Pangti, 2021 [10]	❖ Hellenic Dermatological Atlas + DanDerm	40	282/15,418	5, 7, 8, 9, 12, 14, 99	MD, PS-NR	DenseNet-161	0.980	NR	0.846 (0.719 – 0.931)	0.989	0.454	RoB	+	+	+
													App	?	+	+
5	Raha, 2024 [96]	MSID + DermNet	8	NR/1,285	6, 10, 14, 99	MD, MC	Proposed attention-based MobileNetV2	NR	0.923	NR	NR	NR	RoB	-	+	-
													App	-	?	-
6	Zaar, 2020 [11]	❖ Department of Dermatology and Venereology at Sahlgrenska University Hospital in Gothenburg (Apr-2018 to May-2019)	44	NR	44 conditions	PS-NR	NR	NR	Top-1 Accuracy: 0.749 Top-5 Accuracy: 1.000	NR	NR	NR	RoB	+	?	+
													App	?	+	+

AUC-ROC, Area Under the Curve-Receiver Operating Characteristic; PPV, Positive Predictive Value; P: Population; I: Index Test; R: Reference Standard; RoB: Risk of Bias; App: Applicability; NR: Not Reported; MD: Model Development and Internal Validation; MC: Model Comparison; EV: External Validation; PS-NR: Prospective study (Non-Randomised); CNN: Convolutional Neural Networks;
MSID: Mpox Image Dataset; MSLD: Mpox Skin Lesion Dataset;
◆ *: reviewed by clinician/dermatologist;* ❖ *: confirmed by laboratory and reviewed by clinician/dermatologist*
Reference conditions: (1=Syphilis chancre; 2=Condylomata lata; 3=Syphilis rash; 4=Herpes simplex; 5=Genital warts; 6=Mpox; 7=Molluscum Contagiosum; 8=Tinea Cruris; 9=Lichenoid conditions including lichen sclerosus, lichen planus; 10=Scabies; 11=Folliculitis; 12=Herpes Zoster; 13=Psoriasis; 14=Normal variant/healthy skin; 15=Balanitis; 19=Others)

\+ *Low Concern* ? *Unsure* - *High Concern*

© 2025 Soe NN et al. *JAMA Network Open*.

Dermatosis (Tinea Cruris)

No	Author, Year	Image Data Source	Type of Lesions included	Sample Size (Target/Total)	Reference Conditions	Study Type	Best Performing AI Algorithm	AUC-ROC	Accuracy	Sensitivity	Specificity	PPV		Modified QUADAS-2 P	I	R
1	Bajwa, 2020 [124]	NR	6	49/588	8, 10, 13, 99	MD	ANN (Artificial Neural Network)	NR	NR	NR	NR	NR	RoB	-	-	-
													App	-	-	-
2	Danpakdee, 2017 [123]	Web-scrapped	4	25/100	7, 12, 99	MD,MC	Multi-layer perceptron with Back propagation (MLP)	NR	NR	NR	NR	NR	RoB	-	-	-
													App	-	-	-
3	Gaffoor, 2023 [2]	Kaggle	10	100/1,000	4, 5, 6, 7, 8, 13, 99	MD, MC	SVM	NR	0.100	0.100	NR	0.100	RoB	-	?	-
													App	-	-	-
4	Hestiningsih, 2023 [125]	◆ Google + DermNetnz	3	48/180	10, 99	MD, MC	NASNetMobile	NR	0.916	0.916	NR	0.920	RoB	-	?	-
													App	-	-	-
5	Liu, 2020 [126]	◆ Private dataset from a teledermatology service serving 17 primary care and specialist sites from 2 states in the U.S (2010-2018)	26	294/20,838	11, 99	MD,MC (comparison with clinician), EV	Inception-v4	NR	0.400	0.230	NR	NR	RoB	+	+	+
													App	?	?	+
6	Mehta, 2024 [3]	❖ Private dataset from STI and genital diseases clinic in North India (Jan-2021 to Jun-2022)	8	15/257	4, 5, 6, 7, 8, 9, 10, 99	PS-NR	CNN-based app (DermAId)	NR	0.689	0.933	0.992	NR	RoB	+	+	+
													App	-	+	+
7	Pangti, 2021 [10]	❖ Hellenic Dermatological Atlas + DanDerm	40	1,096/15,418	5, 7, 8, 9, 12, 14, 99	MD, PS-NR	DenseNet-161	0.950	NR	0.817 (0.782 – 0.848)	0.968	0.755	RoB	+	+	+
													App	?	+	+

© 2025 Soe NN et al. *JAMA Network Open*.

8	Shen, 2024 [127]	❖ Web-scraped + dermatologists' personal channels + publicly available resources in China (ImageNet, Derm)	22	8,672/133,297	22 conditions, (4, 8, 9, 11, 99)	MD, PS-NR	SwAV (Swapping Assignments between multiple Views) contrastive learning	NR	NR	0.280	NR	NR	**RoB**	?	+	+
													**App**	?	+	+

AUC-ROC, Area Under the Curve-Receiver Operating Characteristic; PPV, Positive Predictive Value; P: Population; I: Index Test; R: Reference Standard; RoB: Risk of Bias; App: Applicability; NR: Not Reported; MD: Model Development and Internal Validation; MC: Model Comparison; EV: External Validation; PS-NR: Prospective study (Non-Randomised); CNN: Convolutional Neural Networks;
MSID: Mpox Image Dataset; MSLD: Mpox Skin Lesion Dataset;
◆ *: reviewed by clinician/dermatologist;* ❖ *: confirmed by laboratory and reviewed by clinician/dermatologist*
Reference conditions: (1=Syphilis chancre; 2=Condylomata lata; 3=Syphilis rash; 4=Herpes simplex; 5=Genital warts; 6=Mpox; 7=Molluscum Contagiosum; 8=Tinea Cruris; 9=Lichenoid conditions including lichen sclerosus, lichen planus; 10=Scabies; 11=Folliculitis; 12=Herpes Zoster; 13=Psoriasis; 14=Normal variant/healthy skin; 15=Balanitis; 19=Others)

\+ *Low Concern* ? *Unsure* - *High Concern*

eTable 2f: Lichenification (Lichen Sclerosus, Lichen Planus)

No	Author, Year	Image Data Source	Type of Lesions included	Sample Size (Target/Total)	Reference Conditions	Study Type	Best Performing AI Algorithm	AUC-ROC	Accuracy	Sensitivity	Specificity	PPV		Modified QUADAS-2		
														**P**	**I**	**R**
1	Chaurasia, 2019 [128]	NR	7	NR	99	MD,MC	Support Vector Machine (SVM)	NR	0.987	1.000	NR	1.000	**RoB**	?	?	-
													**App**	-	-	-
2	Ieee, 2020 [129]	◆ Dermnet + Web-scraped	7	NR/3,000	99	MD	CNN model (customized)	NR	0.730	0.680	NR	0.640	**RoB**	-	?	-
													**App**	-	-	-
3	Mehta, 2024 [3]	❖ Private dataset from STI and genital diseases clinic in North India (Jan-2021 to Jun-2022)	8	60/257	4, 5, 6, 7, 8, 9, 10, 99	PS-NR	CNN-based app (DermAId)	NR	0.689	0.559	0.965	NR	**RoB**	+	+	+
													**App**	-	+	+
4	Pangti, 2021 [10]	❖ Hellenic Dermatological Atlas + DanDerm	40	220/15,418	5, 7, 8, 9, 12, 14, 99	MD	DenseNet-161	0.880	NR	Lichen planus: 0.696	Lichen planus: 0.957	Lichen planus: 0.362	**RoB**	+	+	+

© 2025 Soe NN et al. *JAMA Network Open*.

										(0.621 - 0.764) Lichen sclerosus: 0.444 (0.215 - 0.692)	Lichen sclerosus: 0.999	Lichen sclerosus: 0.615	**App**	?	+	+
5	Shen, 2024 [127]	❖ Web-scraped + dermatologists' personal channels + publicly available resources in China (ImageNet, Derm)	22	8,672/133,297	22 conditions, (4, 8, 9, 11, 99)	MD, PS-NR	SwAV (Swapping Assignments between multiple Views) contrastive learning	NR	0.306	0.160	NR	NR	**RoB**	?	+	+
													**App**	?	+	+
6	Zaar, 2020 [11]	❖ Department of Dermatology and Venereology at Sahlgrenska University Hospital in Gothenburg (Apr-2018 to May-2019)	44	NR	44 conditions	PS-NR	NR	NR	Top-1 Accuracy: 0.528 Top-5 Accuracy: 0.108	NR	NR	NR	**RoB**	+	?	+
													**App**	?	+	+

AUC-ROC, Area Under the Curve-Receiver Operating Characteristic; PPV, Positive Predictive Value; P: Population; I: Index Test; R: Reference Standard; RoB: Risk of Bias; App: Applicability; NR: Not Reported; MD: Model Development and Internal Validation; MC: Model Comparison; EV: External Validation; PS-NR: Prospective study (Non-Randomised); CNN: Convolutional Neural Networks;
MSID: Mpox Image Dataset; MSLD: Mpox Skin Lesion Dataset;
◆ *: reviewed by clinician/dermatologist;* ❖ *: confirmed by laboratory and reviewed by clinician/dermatologist*
Reference conditions: (1=Syphilis chancre; 2=Condylomata lata; 3=Syphilis rash; 4=Herpes simplex; 5=Genital warts; 6=Mpox; 7=Molluscum Contagiosum; 8=Tinea Cruris; 9=Lichenoid conditions including lichen sclerosus, lichen planus; 10=Scabies; 11=Folliculitis; 12=Herpes Zoster; 13=Psoriasis; 14=Normal variant/healthy skin; 15=Balanitis; 19=Others)

+ *Low Concern* ? *Unsure* - *High Concern*

eTable 2g: Scabies

No	Author, Year	Image Data Source	Type of Lesions included	Sample Size (Target/Total)	Reference Conditions	Study Type	Best Performing AI Algorithm	AUC-ROC	Accuracy	Sensitivity	Specificity	PPV		Modified QUADAS-2		
														P	I	R
1	Andryani, 2023 [130]	ISIC	6	226/1,166	99	MD	Inception ResNet-V2	NR	0.770	0.790	NR	0.800	**RoB**	-	-	-
													**App**	-	-	-
2	Aziz, 2023 [131]	Private dataset from Indonesia	2	200/400	14	MD	Support Vector Machine (SVM)	NR	0.990	0.98	NR	0.980	**RoB**	?	-	-
													**App**	?	-	-

© 2025 Soe NN et al. *JAMA Network Open*.

3	Bajwa, 2020 [124]	NR	6	224/588	8, 10, 13, 99	MD	ANN (Artificial Neural Network)	NR	NR	NR	NR	NR	RoB	-	-	-
													App	-	-	-
4	Mehta, 2024 [3]	❖ Private dataset from STI and genital diseases clinic in North India (Jan-2021 to Jun-2022)	8	8/257	4, 5, 6, 7, 8, 9, 10, 99	PS-NR	CNN-based app (DermAId)	NR	0.689	0.625	1000	NR	RoB	+	+	+
													App	-	+	+
5	Oraño, 2023 [132]	DermNet	8	369/4,500	13, 14, 99	MC	CNN	NR	0.849	0.919	NR	NR	RoB	-	+	?
													App	-	-	?
6	Raha, 2024 [96]	MSID + Dermnet	8	105/1,285	6, 7, 14, 99	MD, MC	Proposed attention-based MobileNetV2	NR	0.923	NR	NR	NR	RoB	-	+	-
													App	-	?	-
7	Yotsu, 2023 [133]	Prospectively collected in the West African countries	5	389/1,286	99	MD, MC	ResNet-50	NR	0.846	NR	NR	NR	RoB	+	?	+
													App	?	-	+
8	Nurul, 2019 [6]	NR	6	NR/72	4	MD	Local Binary Pattern (LBP) + CNN	NR	0.900	NR	NR	NR	RoB	?	-	-
													App	-	-	-

AUC-ROC, Area Under the Curve-Receiver Operating Characteristic; PPV, Positive Predictive Value; P: Population; I: Index Test; R: Reference Standard; RoB: Risk of Bias; App: Applicability; NR: Not Reported; MD: Model Development and Internal Validation; MC: Model Comparison; EV: External Validation; PS-NR: Prospective study (Non-Randomised); CNN: Convolutional Neural Networks;
MSID: Mpox Image Dataset; MSLD: Mpox Skin Lesion Dataset;
◆ *: reviewed by clinician/dermatologist;* ❖ *: confirmed by laboratory and reviewed by clinician/dermatologist*
Reference conditions: (1=Syphilis chancre; 2=Condylomata lata; 3=Syphilis rash; 4=Herpes simplex; 5=Genital warts; 6=Mpox; 7=Molluscum Contagiosum; 8=Tinea Cruris; 9=Lichenoid conditions including lichen sclerosus, lichen planus; 10=Scabies; 11=Folliculitis; 12=Herpes Zoster; 13=Psoriasis; 14=Normal variant/healthy skin; 15=Balanitis; 19=Others)

+ *Low Concern* ? *Unsure* - *High Concern*

eTable 2h: Folliculitis

No	Author, Year	Image Data Source	Type of Lesions included	Sample Size (Target/Total)	Reference Conditions	Study Type	Best Performing AI Algorithm	AUC-ROC	Accuracy	Sensitivity	Specificity	PPV	Modified QUADAS-2			
														P	I	R
1	Liu, 2020 [126]	◆ Private dataset from a teledermatology service serving 17 primary care and specialist sites from 2 states in the U.S (2010-2018)	26	480/20,838	8, 99	MD, EV, PS-NR	Inception-v4	NR	0.550	0.600	NR	NR	RoB	+	+	+
													App	?	?	+

© 2025 Soe NN et al. *JAMA Network Open*.

2	Shen, 2024 [127]	❖ Web-scraped + dermatologists' personal channels + publicly available resources in China (ImageNet, Derm)	22	900/133,297	22 conditions, (4, 8, 9, 11, 99)	MD, PS-NR	SwAV (Swapping Assignments between multiple Views) contrastive learning	NR	0.565	0.110	NR	NR	**RoB**	?	+	+
													**App**	?	+	+
3	Zaar, 2020 [11]	❖ Department of Dermatology and Venereology at Sahlgrenska University Hospital in Gothenburg (Apr-2018 to May-2019)	44	NR	44 conditions	PS-NR	NR	NR	Top-1 Accuracy: 0.143 Top-5 Accuracy: 0.607	NR	NR	NR	**RoB**	+	?	+
													**App**	?	+	+

AUC-ROC, Area Under the Curve-Receiver Operating Characteristic; PPV, Positive Predictive Value; P: Population; I: Index Test; R: Reference Standard; RoB: Risk of Bias; App: Applicability; NR: Not Reported; MD: Model Development and Internal Validation; MC: Model Comparison; EV: External Validation; PS-NR: Prospective study (Non-Randomised); CNN: Convolutional Neural Networks;
MSID: Mpox Image Dataset; MSLD: Mpox Skin Lesion Dataset;
◆ *: reviewed by clinician/dermatologist;* ❖ *: confirmed by laboratory and reviewed by clinician/dermatologist*
Reference conditions: (1=Syphilis chancre; 2=Condylomata lata; 3=Syphilis rash; 4=Herpes simplex; 5=Genital warts; 6=Mpox; 7=Molluscum Contagiosum; 8=Tinea Cruris; 9=Lichenoid conditions including lichen sclerosus, lichen planus; 10=Scabies; 11=Folliculitis; 12=Herpes Zoster; 13=Psoriasis; 14=Normal variant/healthy skin; 15=Balanitis; 19=Others)

\+ *Low Concern* ? *Unsure* - *High Concern*

eTable 2i: Herpes Zoster

No	Author, Year	Image Data Source	Type of Lesions included	Sample Size (Target/Total)	Reference Conditions	Study Type	Best Performing AI Algorithm	AUC-ROC	Accuracy	Sensitivity	Specificity	PPV		Modified QUADAS-2		
														P	I	R
1	Back, 2021 [134]	SD-HZ dataset (SD-198 + SD-260 + Custom dataset HZ-W)	5	412/8,371	99	MD	Deep Neural Network Ensemble-E	0.927	0.920	0.830	NR	0.970	**RoB**	?	+	+
													**App**	?	-	?
2	Danpakdee, 2017 [123]	Web-scrapped	4	25/100	7, 8, 99	MD,MC	Multi-layer perceptron with Back propagation (MLP)	NR	NR	NR	NR	NR	**RoB**	-	-	-
													**App**	-	-	-
3		Web-scraped	2	622/1,262	99			0.990	0.950	0.960	0.940	0.940	**RoB**	-	?	-

© 2025 Soe NN et al. *JAMA Network Open*.

	Eze, 2023 [135]					MD, MC	MSHA (novel multi-modal contextual fusion model)						App	-	-	-
4	Krammer, 2022 [136]	◆ Private dataset at the Department of Dermatology and Allergy, University Hospital, LMU Munich	10	1,510/34,665	13, 99	MD,MC	EfficientNet-B4	NR	0.957	0.907	NR	0.917	RoB	+	+	+
													App	-	-	?
5	Mejia, 2022 [137]	◆ NR	Not relevant	NR/1,000	14 (lesion detection model)	MD	CNN (Lesion detection model)	NR	0.896	NR	1000	NR	RoB	-	?	?
													App	-	-	?
6	Negi, 2024 [138]	Web-scraped	2	650/1250	14	MD	CNN with GELU activation and Nadam optimizer	NR	0.971	NR	NR	NR	RoB	-	?	-
													App	-	-	-
7	Pangti, 2021 [10]	❖ Hellenic Dermatological Atlas + DanDerm	40	251/15,418	5, 7, 8, 9, 12, 14, 99	MD, PS-NR	DenseNet-161	0.890	NR	0.667 (0.498 - 0.809)	0.996	0.565	RoB	+	+	+
													App	?	+	+
8	Shen, 2024 [127]	❖ Web-scraped + dermatologists' personal channels + publicly available resources in China (ImageNet, Derm)	22	999/133,297	22 conditions, (4, 8, 9, 11, 99)	MD, PS-NR	SwAV (Swapping Assignments between multiple Views) contrastive learning	NR	NR	0.710	NR	NR	RoB	?	+	+
													App	?	+	+

AUC-ROC, Area Under the Curve-Receiver Operating Characteristic; PPV, Positive Predictive Value; P: Population; I: Index Test; R: Reference Standard; RoB: Risk of Bias; App: Applicability; NR: Not Reported; MD: Model Development and Internal Validation; MC: Model Comparison; EV: External Validation; PS-NR: Prospective study (Non-Randomised); CNN: Convolutional Neural Networks;
MSID: Mpox Image Dataset; MSLD: Mpox Skin Lesion Dataset;
◆ *: reviewed by clinician/dermatologist;* ❖ *: confirmed by laboratory and reviewed by clinician/dermatologist*
Reference conditions: (1=Syphilis chancre; 2=Condylomata lata; 3=Syphilis rash; 4=Herpes simplex; 5=Genital warts; 6=Mpox; 7=Molluscum Contagiosum; 8=Tinea Cruris; 9=Lichenoid conditions including lichen sclerosus, lichen planus; 10=Scabies; 11=Folliculitis; 12=Herpes Zoster; 13=Psoriasis; 14=Normal variant/healthy skin; 15=Balanitis; 19=Others)

\+ *Low Concern* ? *Unsure* - *High Concern*

eTable 2j: Psoriasis

No	Author, Year	Image Data Source	Type of Lesions included	Sample Size (Target/Total)	Reference Conditions	Study Type	Best Performing AI Algorithm	AUC-ROC	Accuracy	Sensitivity	Specificity	PPV	Modified QUADAS-2

© 2025 Soe NN et al. *JAMA Network Open*.

														P	I	R
1	Bajwa, 2020 [124]	NR	6	98/588	8, 10, 13, 99	MD	ANN (Artificial Neural Network)	NR	NR	NR	NR	NR	RoB	-	-	-
													App	-	-	-
2	Casuayan, 2020 [9]	◆ Private dataset	7	100/600	14, 99	MD	Support Vector Machine (SVM)	NR	NR	0.867	NR	0.867	RoB	+	?	+
													App	-	-	?
3	Chaurasia, 2019 [128]	NR	7	NR	99	MD	Support Vector Machine (SVM)	NR	0.986	1.000	NR	1.000	RoB	?	?	-
													App	-	-	-
4	Gaffoor, 2023 [2]	Kaggle	10	100/1,000	4, 5, 6, 7, 8, 13, 99	MD, PS-NR	SVM	NR	0.100	0.100	NR	0.100	RoB	-	?	-
													App	-	-	-
5	Oraño, 2023 [132]	DermNet	8	677/4,500	10, 14, 99	MC	CNN	NR	0.849	0.838	NR	NR	RoB	-	+	?
													App	-	-	?
6	Pangti, 2021 [10]	❖ Hellenic Dermatological Atlas + DanDerm	40	873/15,418	5, 7, 8, 9, 12, 14, 99	MD	Modified Densenet-161 with optimised augmentation and inference pipelines	0.930	NR	0.777 (0.732 - 0.817)	0.961	0.628	RoB	+	+	+
													App	?	+	+
7	Zaar, 2020 [11]	❖ Department of Dermatology and Venereology at Sahlgrenska University Hospital in Gothenburg (Apr-2018 to May-2019)	44	NR	44 conditions	PS-NR	NR	NR	Top-1 Accuracy: 0.399; Top-5 Accuracy: 0.111	NR	NR	NR	RoB	+	?	+
													App	?	+	+

AUC-ROC, Area Under the Curve-Receiver Operating Characteristic; PPV, Positive Predictive Value; P: Population; I: Index Test; R: Reference Standard; RoB: Risk of Bias; App: Applicability; NR: Not Reported; MD: Model Development and Internal Validation; MC: Model Comparison; EV: External Validation; PS-NR: Prospective study (Non-Randomised); CNN: Convolutional Neural Networks;
MSID: Mpox Image Dataset; MSLD: Mpox Skin Lesion Dataset;
◆ *: reviewed by clinician/dermatologist;* ❖ *: confirmed by laboratory and reviewed by clinician/dermatologist*
Reference conditions: (1=Syphilis chancre; 2=Condylomata lata; 3=Syphilis rash; 4=Herpes simplex; 5=Genital warts; 6=Mpox; 7=Molluscum Contagiosum; 8=Tinea Cruris; 9=Lichenoid conditions including lichen sclerosus, lichen planus; 10=Scabies; 11=Folliculitis; 12=Herpes Zoster; 13=Psoriasis; 14=Normal variant/healthy skin; 15=Balanitis; 19=Others)

+ *Low Concern* ? *Unsure* - *High Concern*

eTable 2k: Candidiasis

No	Author, Year	Image Data Source	Type of Lesions included	Sample Size (Target/Total)	Reference Conditions	Study Type	Best Performing AI Algorithm	AUC-ROC	Accuracy	Sensitivity	Specificity	PPV		Modified QUADAS-2		
														P	I	R
1			40	305/15,418			DenseNet-161	0.810	NR		0.998	0.412	RoB	+	+	+

© 2025 Soe NN et al. *JAMA Network Open.*

	Pangti, 2021 [10]	❖ Hellenic Dermatological Atlas + DanDerm			5, 7, 8, 9, 12, 14, 99	MD, PS-NR				0.412 (0.184–0.671)			App	?	+	+

AUC-ROC, Area Under the Curve-Receiver Operating Characteristic; PPV, Positive Predictive Value; P: Population; I: Index Test; R: Reference Standard; RoB: Risk of Bias; App: Applicability; NR: Not Reported; MD: Model Development and Internal Validation; MC: Model Comparison; EV: External Validation; PS-NR: Prospective study (Non-Randomised); CNN: Convolutional Neural Networks;
MSID: Mpox Image Dataset; MSLD: Mpox Skin Lesion Dataset;
◆ *: reviewed by clinician/dermatologist;* ❖ *: confirmed by laboratory and reviewed by clinician/dermatologist*
Reference conditions: (1=Syphilis chancre; 2=Condylomata lata; 3=Syphilis rash; 4=Herpes simplex; 5=Genital warts; 6=Mpox; 7=Molluscum Contagiosum; 8=Tinea Cruris; 9=Lichenoid conditions including lichen sclerosus, lichen planus; 10=Scabies; 11=Folliculitis; 12=Herpes Zoster; 13=Psoriasis; 14=Normal variant/healthy skin; 15=Balanitis; 19=Others)

\+ *Low Concern* ? *Unsure* - *High Concern*

eTable 2l: Balanitis

No	Author, Year	Image Data Source	Type of Lesions included	Sample Size (Target/Total)	Reference Conditions	Study Type	Best Performing AI Algorithm	AUC-ROC	Accuracy	Sensitivity	Specificity	PPV		Modified QUADAS-2 P	I	R
1	Allan, 2024 [1]	◆ Multiple (India, Sri Lanka, Singapore, Australia, United States, United Kingdom)	6	290/1,570	1, 2, 4, 5, 15, 99	MD	U-Net + Inception-ResNet-V2	NR	0.944	0.875 (0.732-0.958)	0.995 (0.972 - 0.999)	0.972 (0.919 - 0.999)	RoB	+	+	+
													App	?	+	+
2	Zaar, 2020 [11]	❖ Department of Dermatology and Venereology at Sahlgrenska University Hospital in Gothenburg (Apr-2018 to May-2019)	44	NR	44 conditions	PS-NR	NR	NR	Top-1 Accuracy: 0.331 Top-5 Accuracy: 0.778	NR	NR	NR	RoB	+	?	+
													App	?	+	+

AUC-ROC, Area Under the Curve-Receiver Operating Characteristic; PPV, Positive Predictive Value; P: Population; I: Index Test; R: Reference Standard; RoB: Risk of Bias; App: Applicability; NR: Not Reported; MD: Model Development and Internal Validation; MC: Model Comparison; EV: External Validation; PS-NR: Prospective study (Non-Randomised); CNN: Convolutional Neural Networks;
MSID: Mpox Image Dataset; MSLD: Mpox Skin Lesion Dataset;
◆ *: reviewed by clinician/dermatologist;* ❖ *: confirmed by laboratory and reviewed by clinician/dermatologist*
Reference conditions: (1=Syphilis chancre; 2=Condylomata lata; 3=Syphilis rash; 4=Herpes simplex; 5=Genital warts; 6=Mpox; 7=Molluscum Contagiosum; 8=Tinea Cruris; 9=Lichenoid conditions including lichen sclerosus, lichen planus; 10=Scabies; 11=Folliculitis; 12=Herpes Zoster; 13=Psoriasis; 14=Normal variant/healthy skin; 15=Balanitis; 19=Others)

\+ *Low Concern* ? *Unsure* - *High Concern*

eTable 2m: Penile Cancer

© 2025 Soe NN et al. *JAMA Network Open*.

No	Author, Year	Image Data Source	Type of Lesions included	Sample Size (Target/Total)	Reference Conditions	Study Type	Best Performing AI Algorithm	AUC-ROC	Accuracy	Sensitivity	Specificity	PPV	Modified QUADAS-2			
														P	I	R
1	Allan, 2024 [1]	◆ Multiple (India, Sri Lanka, Singapore, Australia, United States, United Kingdom)	6	211/1,570	1, 2, 4, 5, 15, 99	MD	U-Net + Inception-ResNet-V2	NR	0.944	0.793 (0.603-0.920)	0.986 (0.959 - 0.997)	0.885 (0.762 - 0.999)	RoB	+	+	+
													App	?	+	+

AUC-ROC, Area Under the Curve-Receiver Operating Characteristic; PPV, Positive Predictive Value; P: Population; I: Index Test; R: Reference Standard; RoB: Risk of Bias; App: Applicability; NR: Not Reported; MD: Model Development and Internal Validation; MC: Model Comparison; EV: External Validation; PS-NR: Prospective study (Non-Randomised); CNN: Convolutional Neural Networks;
MSID: Mpox Image Dataset; MSLD: Mpox Skin Lesion Dataset;
◆ *: reviewed by clinician/dermatologist;* ❖ *: confirmed by laboratory and reviewed by clinician/dermatologist*
Reference conditions: (1=Syphilis chancre; 2=Condylomata lata; 3=Syphilis rash; 4=Herpes simplex; 5=Genital warts; 6=Mpox; 7=Molluscum Contagiosum; 8=Tinea Cruris; 9=Lichenoid conditions including lichen sclerosus, lichen planus; 10=Scabies; 11=Folliculitis; 12=Herpes Zoster; 13=Psoriasis; 14=Normal variant/healthy skin; 15=Balanitis; 19=Others)

+ *Low Concern* ? *Unsure* - *High Concern*

eTable 2n: Multiple sexually transmitted infections

No	Author, Year	Image Data Source	Type of Lesions included	Sample Size (Target/Total)	Reference Conditions	Study Type	Best Performing AI Algorithm	AUC-ROC	Accuracy	Sensitivity	Specificity	PPV	Modified QUADAS-2			
														P	I	R
1	Soe, 2024 [139]	❖ Melbourne Sexual Health Centre (Jan-2010 to Jan-2023)	15	1,583 (STIs)/4,913	8, 9, 14, 15, 99	MD, MC	CNN+ FCN (Fully Connected Neural Network)	0.893 ± 0.018	0.692 ± 0.093	0.951 ± 0.003	0.622 ± 0.116	0.433 ± 0.061	RoB	+	+	+
													App	?	+	+

AUC-ROC, Area Under the Curve-Receiver Operating Characteristic; PPV, Positive Predictive Value; P: Population; I: Index Test; R: Reference Standard; RoB: Risk of Bias; App: Applicability; NR: Not Reported; MD: Model Development and Internal Validation; MC: Model Comparison; EV: External Validation; PS-NR: Prospective study (Non-Randomised); CNN: Convolutional Neural Networks;
MSID: Mpox Image Dataset; MSLD: Mpox Skin Lesion Dataset;
◆ *: reviewed by clinician/dermatologist;* ❖ *: confirmed by laboratory and reviewed by clinician/dermatologist*
Reference conditions: (1=Syphilis chancre; 2=Condylomata lata; 3=Syphilis rash; 4=Herpes simplex; 5=Genital warts; 6=Mpox; 7=Molluscum Contagiosum; 8=Tinea Cruris; 9=Lichenoid conditions including lichen sclerosus, lichen planus; 10=Scabies; 11=Folliculitis; 12=Herpes Zoster; 13=Psoriasis; 14=Normal variant/healthy skin; 15=Balanitis; 19=Others)

© 2025 Soe NN et al. *JAMA Network Open*.

+ *Low Concern* ? *Unsure* - *High Concern*

© 2025 Soe NN et al. *JAMA Network Open*.

eTable 3. CLEAR Derm[140] Checklist for included studies

No	Author, Year	Data															Technique				Technical Assessment				Application	
		Image types	Image artifacts (e.g., image quality, pen markings)	Technical acquisition details	Preprocessing procedures	Synthetic images made public if used	Public images adequately referenced	Patient-level metadata: sex and gender distribution, etc.	Skin tone information and procedure for assessment	Potential biases by patient information and metadata	Data set partitions	Sample sizes of training, validation, and test sets	External test set	Multivendor images	Class distribution and balance	Out-of-distribution images	Labeling method	References to accepted diagnostic labels	Histopathologic review for malignant neoplasms	Detailed description of algorithm development	How to publicly evaluate algorithm	Performance measures	Benchmarking, technical comparison, and novelty	Bias assessment	Use cases and target conditions	Potential impacts on healthcare team & patients
1	Abdelhamid, 2022	-	-	-	+	ø	+	-	-	-	+	-	-	-	-	-	-	-	ø	+	-	+	-	-	-	-
2	Agrawal, 2022	-	-	-	+	ø	+	-	-	-	+	+	-	-	-	-	-	-	ø	+	-	+	+	?	-	-
3	Ahsan, 2024	-	-	-	?	ø	+	-	-	-	+	+	-	-	-	-	-	-	ø	+	-	+	+	-	-	-
4	Ahsan, 2023	-	-	-	?	ø	+	-	-	-	+	+	-	-	-	-	-	-	ø	+	-	+	?	-	-	-
5	Ahsan, 2023	-	-	-	?	ø	?	-	-	-	+	+	-	-	-	-	-	-	ø	+	-	+	+	-	-	-
6	Alcalá-Rmz, 2023	-	-	-	+	ø	?	-	-	-	+	?	-	-	-	-	-	-	ø	+	-	?	?	-	-	-
7	Alharbi, 2023	-	-	-	?	ø	?	-	-	-	+	?	-	-	-	-	-	-	ø	+	-	+	+	-	-	-
8	Alhasson, 2023	-	-	-	?	ø	?	-	-	-	+	+	-	-	-	-	-	-	ø	+	-	+	?	-	-	-
9	Almufareh, 2023	-	-	-	+	ø	+	-	-	-	+	+	-	-	-	-	-	-	ø	+	-	+	+	-	-	-
10	Almutairi, 2022	?	-	-	?	ø	+	-	-	-	+	+	-	-	-	-	-	-	ø	+	-	+	?	-	-	-
11	Aloraini, 2024	-	-	-	-	ø	+	-	-	-	+	?	-	-	-	-	-	-	ø	+	-	+	+	-	-	-
12	Alrusaini, 2023	-	-	-	?	ø	-	-	-	-	+	?	-	-	-	-	?	-	ø	+	-	+	+	-	-	-
13	Altun, 2023	-	-	-	?	ø	?	-	-	-	+	?	-	-	-	-	-	-	ø	+	-	+	+	-	-	-
14	Amin, 2023	-	-	-	?	ø	+	-	-	-	+	?	-	-	-	-	-	-	ø	+	-	+	+	-	-	-
15	Andryani, 2023	-	-	-	+	ø	?	-	-	-	+	?	-	-	-	-	-	-	ø	+	-	+	+	-	-	-
16	Arshed, 2024	?	-	-	+	ø	+	-	-	-	+	+	-	-	-	-	-	-	ø	+	-	+	+	-	-	-
17	Asif, 2024	-	-	-	+	ø	+	-	-	-	+	+	+	-	-	-	-	-	ø	+	-	+	+	-	-	-
18	Asif, 2023	-	-	-	+	ø	+	-	-	-	+	+	+	-	-	-	-	-	ø	+	-	+	+	-	-	-
19	Assoc, 2019	-	-	-	+	ø	?	-	-	-	?	?	-	-	-	-	-	-	ø	+	-	?	?	-	-	-
20	Attallah, 2023	-	-	-	+	ø	+	-	-	-	+	+	-	-	-	-	-	-	ø	+	-	+	+	-	-	-
21	Aziz, 2023	?	-	?	+	ø	-	-	-	-	+	-	-	-	-	-	-	-	ø	+	-	+	+	-	-	-
22	Back, 2021	?	?	?	+	ø	+	-	-	-	+	+	-	?	-	-	-	-	ø	+	-	+	+	-	-	-
23	Bajwa, 2020	-	?	-	+	ø	-	-	-	-	+	+	-	-	-	-	-	-	ø	+	-	+	+	?	-	-
24	Bala, 2023	+	-	-	+	ø	?	-	-	-	+	+	-	-	-	-	-	-	ø	+	-	+	+	-	-	-
25	Bansal, 2023	-	-	-	+	ø	-	-	-	-	+	+	-	-	-	-	-	-	ø	+	-	+	+	-	-	-

© 2025 Soe NN et al. *JAMA Network Open*.

26	Bogar, 2023	-	-	-	+	ø	+	-	-	-	+	+	-	-	-	-	-	-	ø	+	-	?	+	-	-	-
27	Campana, 2024	-	-	-	+	ø	+	-	+	+	+	+	-	-	+	-	-	-	ø	+	-	+	+	+	-	-
28	Casuayan, 2020	-	-	-	+	ø	ø	-	+	-	+	+	-	-	-	-	+	-	ø	+	-	?	?	-	-	-
29	Chandrahaas, 2023	-	-	-	-	ø	-	-	-	-	?	?	-	-	-	-	-	-	ø	?	-	?	?	-	-	-
30	Chauhan, 2023	-	-	-	-	ø	?	-	-	-	+	+	-	-	-	-	-	-	ø	?	-	+	+	-	-	-
31	Chaurasia, 2019	-	-	-	+	ø	-	-	-	-	?	-	-	-	-	-	-	-	ø	+	-	+	+	-	-	-
32	Chen, 2023	-	-	-	+	ø	+	-	-	-	+	?	+	-	-	-	-	-	ø	+	-	+	+	-	-	-
33	Chen, 2023	-	-	-	?	ø	+	-	-	-	+	+	+	-	-	-	-	-	ø	+	-	+	+	-	-	-
34	Chintamaneni, 2024	-	-	-	+	ø	+	-	-	-	+	?	-	-	-	-	-	-	ø	+	-	+	+	-	-	-
35	Ciran, 2023	?	-	-	+	ø	+	-	-	-	-	?	-	-	-	-	-	-	ø	+	-	+	+	-	-	-
36	Dahiya, 2023	-	-	-	+	ø	+	-	-	-	+	+	-	-	-	-	-	-	ø	+	-	+	+	-	-	-
37	Dan, 2022	-	?	-	+	ø	?	-	-	-	+	+	-	-	-	-	-	-	ø	+	-	+	+	-	-	-
38	Danpakdee, 2017	?	-	-	+	ø	-	-	-	-	-	-	-	-	-	-	-	-	ø	+	-	?	+	-	-	-
39	Dodia, 2022	-	?	-	+	ø	+	-	-	-	?	?	-	-	-	-	-	-	ø	+	-	+	+	-	-	-
40	Dwivedi, 2022	-	-	-	+	ø	+	-	-	-	+	-	-	-	-	-	-	-	ø	+	-	?	+	-	-	-
41	Eliwa, 2023	-	-	-	-	?	+	-	-	-	+	-	-	-	-	-	-	-	ø	+	-	+	+	-	-	-
42	Eze, 2023	-	-	-	+	ø	+	-	-	-	+	+	-	-	-	-	-	-	ø	+	-	+	+	-	-	-
43	Ezenkwu, 2023	?	-	-	+	ø	+	-	-	-	+	+	-	-	-	-	-	-	ø	+	-	+	+	?	-	-
44	Fisranda, 2023	-	-	-	?	ø	+	-	-	-	+	?	-	-	-	-	-	-	ø	+	-	?	+	-	-	-
45	Gaffoor, 2023	-	?	-	+	ø	-	-	-	-	?	?	-	-	-	-	-	-	ø	?	-	+	-	-	-	-
46	Gairola, 2022	-	-	-	+	ø	+	-	-	-	?	+	-	-	-	-	-	-	ø	+	-	+	+	-	-	-
47	Gupta, 2023	-	-	-	+	ø	-	-	-	-	+	?	-	-	-	-	-	-	ø	?	-	+	+	-	-	-
48	Gupta, 2023	-	-	-	+	ø	+	-	-	-	+	-	-	-	-	-	-	-	ø	+	-	+	+	-	-	-
49	Haque, 2022	-	-	-	+	ø	+	-	-	-	+	?	-	-	-	-	-	-	ø	+	-	+	+	-	-	-
50	Haque, 2023	-	-	-	+	ø	+	-	-	-	+	+	-	-	-	-	-	-	ø	+	-	+	+	-	-	-
51	Harikiran, 2023	-	-	-	+	ø	+	-	-	-	+	+	-	-	-	-	-	-	ø	+	-	+	+	-	-	-
52	Haripriya, 2024	-	-	-	+	ø	+	-	-	-	+	-	-	-	-	-	-	-	ø	+	-	+	+	-	-	-
53	Hestiningsih, 2023	-	-	-	+	ø	?	-	-	-	?	?	-	-	-	-	-	-	ø	+	-	+	+	-	-	-
54	Hossen, 2023	?	-	-	+	ø	+	-	-	-	+	+	-	-	-	-	-	-	ø	+	-	+	+	-	-	-
55	Huong, 2023	?	-	-	+	ø	+	-	-	-	-	-	-	-	-	-	-	-	ø	+	-	+	+	-	-	-
56	Hussain, 2023	-	-	-	-	ø	-	-	-	-	+	+	-	-	-	-	-	-	ø	+	-	+	+	-	-	-
57	Ieee, 2023	?	?	-	+	ø	-	-	-	-	+	+	-	-	-	-	-	-	ø	+	-	+	+	-	-	-
58	Ieee, 2022	-	-	-	-	ø	+	-	-	?	+	+	-	-	-	-	-	-	ø	+	-	+	+	-	-	-
59	Ieee, 2022	-	-	-	+	ø	+	-	-	-	+	+	-	-	-	-	-	-	ø	+	-	+	+	-	-	-
60	Ieee, 2020	-	-	-	+	ø	-	-	-	-	+	+	-	-	-	-	-	-	ø	+	-	+	+	-	-	-
61	Ieee, 2023	-	-	-	+	ø	+	-	-	-	+	+	-	-	-	-	-	-	ø	+	-	+	+	-	-	-
62	Islam, 2022	?	-	-	+	ø	+	-	-	-	+	+	-	-	-	-	+	-	ø	+	-	+	+	-	-	-

© 2025 Soe NN et al. *JAMA Network Open*.

63	Jahan, 2023	?	-	-	+	ø	+	-	-	-	+	+	-	-	-	-	-	-	ø	+	-	+	+	-	-	-
64	Jaradat, 2023	-	-	-	+	ø	+	-	-	-	?	?	+	-	-	-	-	-	ø	+	-	+	+	-	-	-
65	Kakulapati, 2023	-	-	-	+	ø	-	-	-	-	+	?	-	-	-	-	-	-	ø	+	-	+	+	-	-	-
66	Kaushal, 2023	-	-	-	+	ø	+	-	-	-	+	+	-	-	-	-	-	-	ø	+	-	+	+	-	-	-
67	Khafaga, 2022	-	-	-	+	ø	-	-	-	-	-	-	-	-	-	-	-	-	ø	+	-	+	+	-	-	-
68	Khan, 2024	-	-	-	+	ø	-	-	-	-	+	+	-	-	-	-	-	-	ø	+	-	+	+	-	-	-
69	Kottath, 2023	-	-	-	-	ø	-	-	-	-	+	+	-	-	-	-	-	-	ø	+	-	+	+	-	-	-
70	Krammer, 2022	?	-	-	+	ø	ø	-	-	-	+	+	-	-	-	-	+	-	ø	+	-	+	+	-	-	-
71	Krishnan, 2023	-	-	-	+	ø	-	-	-	-	+	+	-	-	-	-	-	-	ø	+	-	+	+	-	-	-
72	Krishnan, 2024	-	-	-	+	ø	-	-	-	-	+	+	-	-	-	-	-	-	ø	+	-	+	+	-	-	-
73	Krishnan, 2023	-	-	-	+	ø	?	-	-	-	+	+	-	-	-	-	-	-	ø	+	-	+	+	-	-	-
74	Kumar, 2023	-	-	-	+	ø	+	-	-	-	+	+	-	-	-	-	+	-	ø	+	-	+	+	?	-	-
75	Kumar, 2022	-	-	-	-	ø	+	-	-	-	+	-	-	-	-	-	-	-	ø	+	-	+	+	-	-	-
76	Kundu, 2024	-	-	-	+	ø	+	-	-	-	+	+	-	-	-	-	-	-	ø	+	-	+	+	-	-	-
77	Kundu, 2022	-	-	-	+	ø	+	-	-	-	+	-	-	-	-	-	-	-	ø	+	-	+	+	-	-	-
78	Kundu, 2023	-	-	-	+	ø	+	-	-	-	+	+	-	-	-	-	-	-	ø	+	-	+	+	-	-	-
79	Lakshmi, 2023	-	?	-	+	ø	+	-	-	-	+	+	-	-	-	-	-	-	ø	+	-	+	+	-	-	-
80	Liu, 2023	-	-	-	+	ø	+	-	-	-	+	+	-	-	-	-	-	-	ø	+	-	+	+	-	-	-
81	Liu, 2023	-	-	-	+	ø	+	-	-	-	+	+	-	-	-	-	-	-	ø	+	-	+	+	-	-	-
82	Liu, 2020	+	-	+	+	ø	ø	+	+	?	+	+	-	+	+	-	+	+	ø	+	-	+	+	+	+	+
83	Madhu, 2023	-	-	-	+	ø	+	-	-	-	+	+	-	-	-	-	-	-	ø	+	-	+	+	-	-	-
84	Magboo, 2023	-	-	-	+	ø	+	-	-	-	+	+	-	-	-	-	-	-	ø	+	-	+	+	-	-	-
85	Meena, 2024	-	-	-	?	ø	+	-	-	-	+	?	-	-	-	-	-	-	ø	+	-	+	+	-	-	-
86	Mehta, 2024	-	?	?	-	ø	ø	-	-	-	ø	ø	-	-	-	+	+	-	+	+	-	+	+	-	+	-
87	Mejia, 2022	-	?	?	+	ø	-	-	-	-	-	?	-	-	-	-	?	-	ø	+	-	+	+	-	?	-
88	Muduli, 2023	-	-	-	+	ø	+	-	-	-	+	+	-	-	-	-	-	-	ø	+	-	+	+	-	-	-
89	Munoz-Saavedra, 2023	?	-	-	+	ø	+	-	-	-	+	+	-	-	-	-	-	-	ø	+	-	+	+	-	-	-
90	Nayak, 2023	?	-	-	-	ø	+	-	-	-	+	+	-	-	-	-	-	-	ø	+	-	+	+	-	-	-
91	Nayak, 2023	-	-	-	+	ø	+	-	-	-	+	+	-	-	-	-	-	-	ø	+	-	+	+	-	-	-
92	Nazmee, 2023	-	-	-	+	ø	+	-	-	-	+	+	-	-	-	-	-	-	ø	+	-	+	+	-	-	-
93	Negi, 2024	-	-	-	+	ø	+	-	-	-	+	+	-	-	-	-	-	-	ø	+	-	+	+	-	-	-
94	Oraño, 2023	-	-	-	-	ø	+	-	-	-	+	+	-	-	-	-	-	-	ø	+	-	+	+	-	?	-
95	Ozaltin, 2023	?	-	-	+	ø	+	-	-	-	+	+	-	-	-	-	-	-	ø	+	-	+	+	-	-	-
96	Oztel, 2023	-	-	-	+	ø	+	?	-	-	+	+	-	-	-	-	-	-	ø	+	-	+	+	-	-	-
97	Pabbi, 2023	-	-	-	+	ø	-	-	-	-	?	?	-	-	-	-	-	-	ø	+	-	+	?	-	-	-
98	Pal, 2023	-	-	-	+	ø	-	-	-	-	+	+	-	-	-	-	-	-	ø	+	-	+	+	-	-	-
99	Pal, 2023	-	-	-	+	ø	+	-	-	-	+	?	+	-	-	-	-	-	ø	+	-	+	+	-	-	-

© 2025 Soe NN et al. *JAMA Network Open*.

100	Pangti, 2021	-	-	-	+	ø	+	ø	-	?	+	+	+	-	-	-	+	-	?	+	-	+	+	?	-	-
101	Pasha, 2023	-	-	-	+	ø	-	-	-	-	-	-	-	-	-	-	-	-	ø	+	-	+	+	-	-	-
102	Pramanik, 2023	-	-	-	+	ø	+	-	-	-	+	+	-	-	-	-	-	-	ø	+	-	+	+	-	-	-
103	Pramanik, 2023	-	-	-	+	ø	+	-	-	-	+	?	-	-	-	-	-	-	ø	+	-	+	+	-	-	-
104	Prasher, 2023	-	-	-	?	ø	?	-	-	-	-	-	-	-	-	-	-	-	ø	+	-	?	?	-	-	-
105	Raha, 2024	-	-	-	+	ø	+	-	-	-	+	+	-	-	-	-	-	-	ø	+	-	+	+	-	-	-
106	Rai, 2023	-	-	-	+	ø	?	-	-	-	+	+	-	-	-	-	-	-	ø	+	-	+	+	-	-	-
107	Rao, 2023	-	-	-	?	ø	-	-	-	-	-	-	-	-	-	-	-	-	ø	+	-	+	+	-	-	-
108	Ren, 2023	-	-	-	?	ø	+	-	-	-	+	+	-	-	-	-	-	-	ø	+	-	+	+	-	-	-
109	Sadik, 2023	-	-	-	+	ø	+	-	-	-	+	+	-	-	-	-	-	-	ø	+	-	+	+	-	-	-
110	Sahin, 2022	-	-	-	+	ø	+	-	-	-	+	+	-	-	-	-	-	-	ø	+	-	+	+	-	?	-
111	Sathwik, 2023	-	-	-	?	ø	+	-	-	-	-	-	-	-	-	-	-	-	ø	+	-	+	+	-	-	-
112	Shah, 2022	-	-	-	+	ø	?	-	-	-	+	+	-	-	-	-	-	-	ø	+	-	+	+	-	-	-
113	Sharma, 2023	-	-	-	?	ø	+	-	-	-	?	?	-	-	-	-	-	-	ø	+	-	+	+	-	-	-
114	Sharma, 2023	-	-	-	+	ø	+	-	-	-	+	+	-	-	-	-	-	-	ø	+	-	+	+	-	-	-
115	Sharma, 2023	-	-	-	+	ø	-	-	-	-	?	?	-	-	-	-	-	-	ø	+	-	+	?	-	-	-
116	Shen, 2024	-	?	?	+	ø	?	?	?	?	+	+	+	-	?	+	+	+	ø	+	-	+	+	+	+	-
117	Singh, 2022	-	-	-	-	ø	+	-	-	-	?	-	-	-	-	-	-	-	ø	?	-	+	+	-	-	-
118	Soe, 2023	?	?	-	+	ø	+	-	-	-	+	+	+	-	?	-	+	-	ø	+	-	+	+	?	?	-
119	Sorayaie, 2023	-	-	-	+	ø	+	-	-	-	+	+	-	-	-	-	-	-	ø	+	-	+	+	-	?	-
120	Supanich, 2023	-	-	-	+	ø	+	-	-	-	+	+	-	-	-	+	-	-	ø	+	-	+	+	-	?	-
121	Surati, 2023	-	-	-	-	ø	?	-	-	-	+	-	-	-	-	-	-	-	ø	+	-	+	+	-	-	-
122	Taruno, 2023	-	-	-	+	ø	?	-	-	-	+	+	-	-	-	-	-	-	ø	+	-	+	+	-	-	-
123	Thieme, 2023	?	?	?	+	ø	+	?	+	+	+	+	+	+	+	-	+	-	ø	+	-	+	?	+	?	-
124	Thorat, 2024	-	-	-	+	ø	+	-	?	?	+	+	-	-	-	-	-	-	ø	+	-	+	+	?	-	-
125	Tiwari, 2023	-	-	-	?	ø	-	-	-	-	+	+	-	-	-	-	-	-	ø	+	-	+	+	-	-	-
126	Torky, 2022	-	-	-	-	ø	-	-	-	-	+	+	-	-	-	-	-	-	ø	+	-	+	+	-	-	-
127	Ural, 2023	-	-	-	+	ø	+	-	-	-	+	+	-	-	-	-	+	-	ø	+	-	+	+	-	-	-
128	Uysal, 2023	-	-	-	+	ø	+	-	-	-	+	+	-	-	-	-	-	-	ø	+	-	+	+	-	-	-
129	Uzun, 2023	-	-	-	-	ø	+	-	-	-	+	+	-	-	-	-	-	-	ø	+	-	+	+	-	-	-
130	Vajpayee, 2023	-	-	-	-	ø	-	-	-	-	+	+	-	-	-	-	-	-	ø	+	-	+	+	-	-	-
131	Wei, 2018	-	-	-	+	ø	ø	-	-	-	+	+	-	-	-	-	-	-	ø	+	-	+	+	-	-	-
132	Yadav, 2023	?	-	-	+	ø	+	-	-	-	-	-	-	-	-	-	-	-	ø	+	-	+	+	-	-	-
133	Yadav, 2024	-	-	-	+	ø	+	-	-	-	-	-	-	-	-	-	-	-	ø	+	-	+	+	-	-	-
134	Yasmin, 2023	-	-	-	+	ø	+	-	-	-	?	?	-	-	-	-	-	-	ø	+	-	+	+	-	-	-
135	Yotsu, 2023	?	?	?	+	ø	ø	+	?	+	+	+	-	-	-	-	+	-	ø	+	-	+	+	+	?	-
136	Zi, 2022	-	-	-	?	ø	?	-	-	-	-	-	-	-	-	-	-	-	ø	+	-	+	+	-	-	-

© 2025 Soe NN et al. *JAMA Network Open*.

137	Allan, 2024	-	-	-	+	ø	ø	?	-	?	+	+	-	-	-	-	+	-	ø	+	-	+	+	?	?	-
138	Zaar, 2020	?	?	?	ø	ø	ø	?	?	+		ø	+	-	?	-	+	+	?	ø	-	+	+	+	+	-
139	Nurul, 2019	-	-	-	?	ø	-	-	-	-	?	?	-	-	-	-	-	-	ø	+	-	?	?	-	-	-
140	Soe, 2024	-	?	?	+	ø	ø	+	-	?	+	+	-	-	+	-	+	-	ø	+	-	+	+	?	?	-

\+ *Present;* ? *Partially Present;* - *Absent;* ø *Not Applicable;*

© 2025 Soe NN et al. *JAMA Network Open*.

eTable 4. Characteristics of included studies

Category	Number of Study	Percentage (%)
Disease Conditions reported[#]		
Mpox	110	62.2
Tinea Cruris	8	4.5
Genital Warts	8	4.5
Scabies	8	4.5
Herpes Zoster	8	4.5
Psoriasis	7	3.9
Herpes Simplex	7	3.9
Lichenoid Changes	6	3.4
Molluscum Contagiosum	6	3.4
Folliculitis	3	1.7
Balanitis	2	1.1
Syphilis chancre and condylomata lata	1	0.6
Candidiasis	1	0.6
Penile Cancer	1	0.6
STIs (Combined)	1	0.6
Image Database Type		
Public	121	86.4
Private	12	8.6
Public + Private	5	3.6
Not Reported	2	1.4
Private Database Region (n=12)		
Asia	4	33.3
Americas	3	25.0
Europe	2	16.7
Oceania	2	16.7
Africa	1	8.3
Image Diagnosis Validated		
No	123	87.9
Yes	17	12.1
Data Input Type		

© 2025 Soe NN et al. *JAMA Network Open*.

Image Only	138	98.6
Image + Metadata	2	1.4
Gender Considerations		
No	136	95.7
Yes	6	4.3
Subgroup Analysis		
No	132	94.3
Yes	8	5.7
Model Interpretability		
No	116	82.9
Yes	24	17.1

[#] *The total number of disease conditions exceeded the number of studies because some studies reported multiple conditions. Percentages in the Disease Conditions reported section are calculated based on the total number of conditions reported.*

© 2025 Soe NN et al. *JAMA Network Open*.

eTable 5. Duplicate images in Mpox datasets

Mpox Dataset	Total Images	Number of Duplicates	% of Duplicates	Sources
**Monkeypox Skin Images Dataset (MSID)**	772	25	3.24%	https://www.kaggle.com/datasets/dipuiucse/monkeypoxskinimagedataset
**Mpox Close Skin Images (MCSI)**	400	8	2.00%	https://zenodo.org/records/7948350
**Mpox Skin Lesion Dataset (MSLD)**	755	5	0.66%	https://www.kaggle.com/datasets/nafin59/monkeypox-skin-lesion-dataset
**MSID Vs MCSI**	1172	95	8.11%	
**MSID Vs MSLD**	1527	66	4.32%	
**MSLD Vs MCSI**	1155	84	7.27%	

© 2025 Soe NN et al. *JAMA Network Open*.

A.

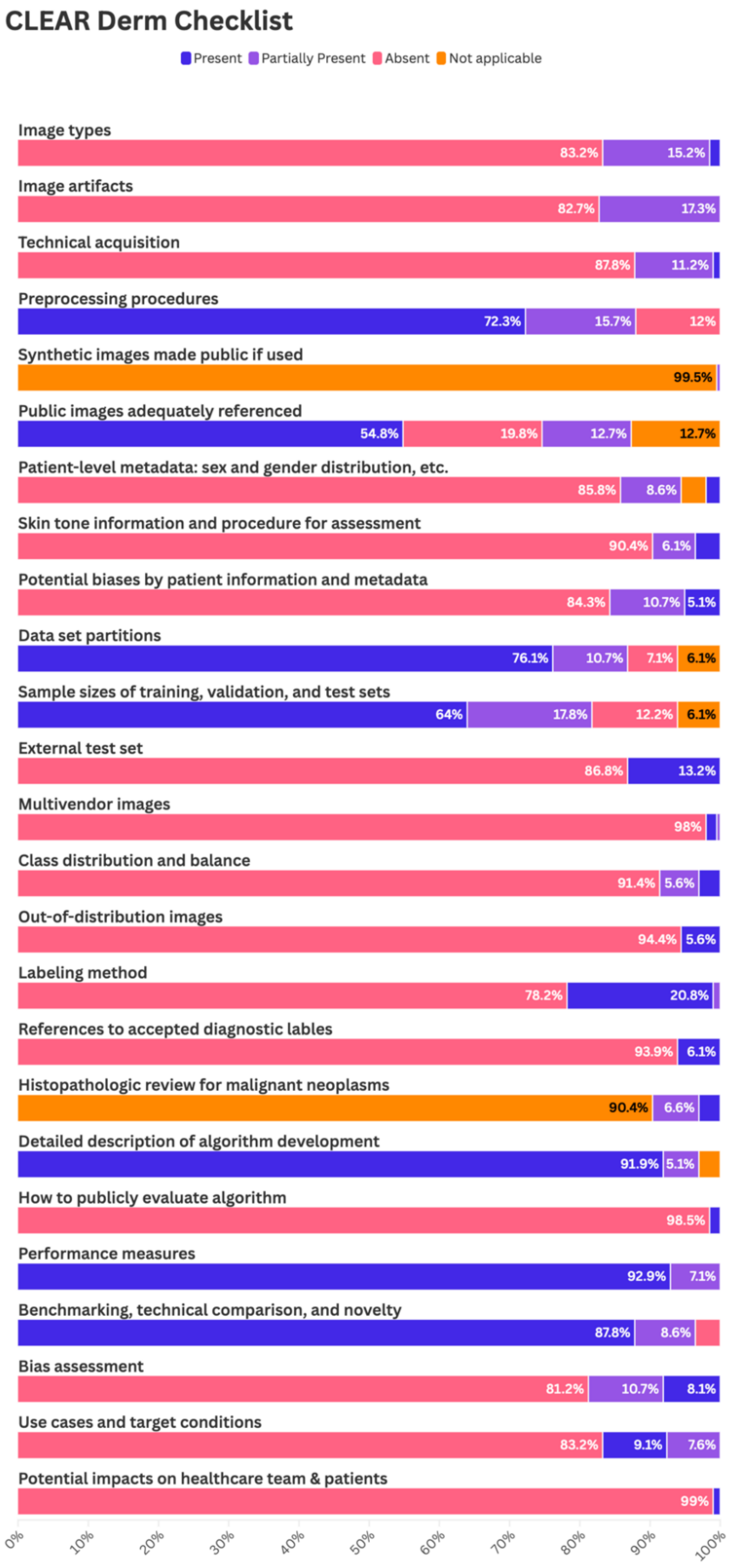

B.

© 2025 Soe NN et al. *JAMA Network Open*.

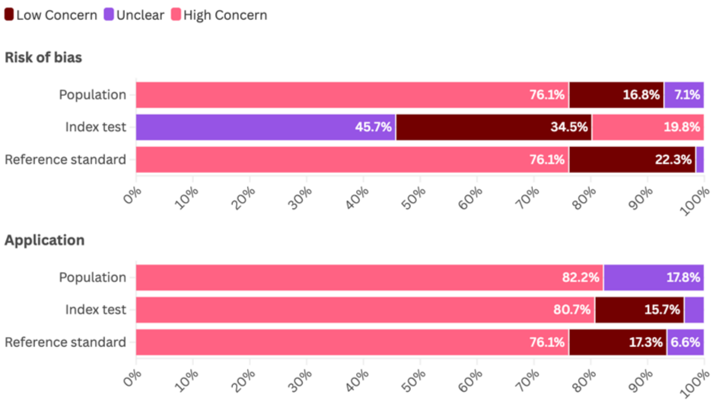

eFigure 1 Quality assessment by (a) CLEAR Derm Checklist and (b) modified QUADAS-2 tool

© 2025 Soe NN et al. *JAMA Network Open*.

eFigure 2. Meta-analysis Findings (Forest plots and SROC graphs)

A. **Mpox**

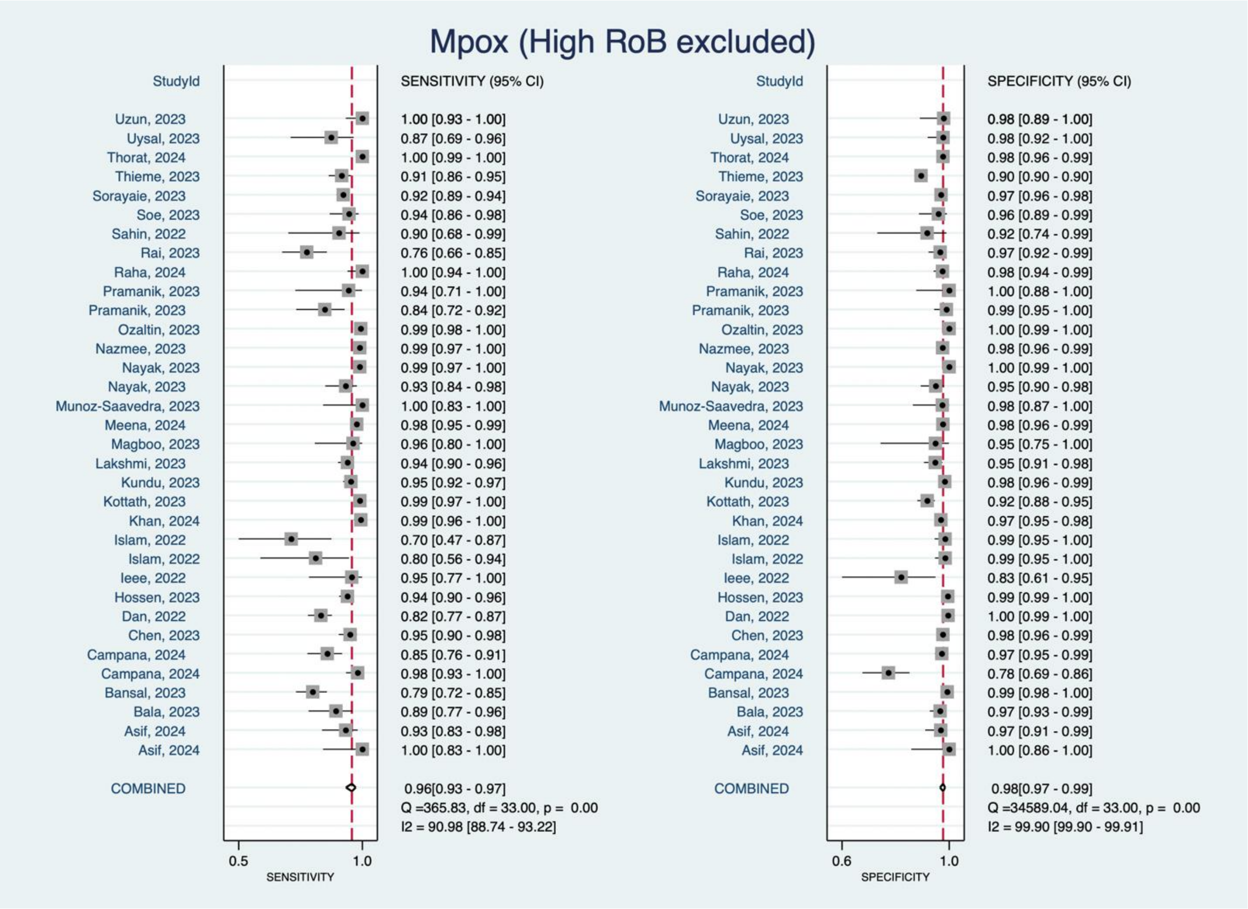

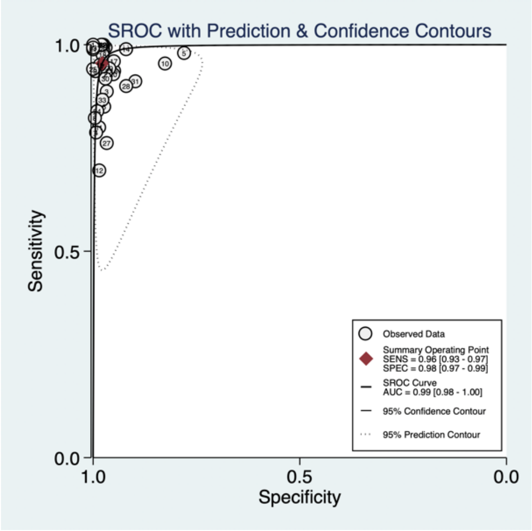

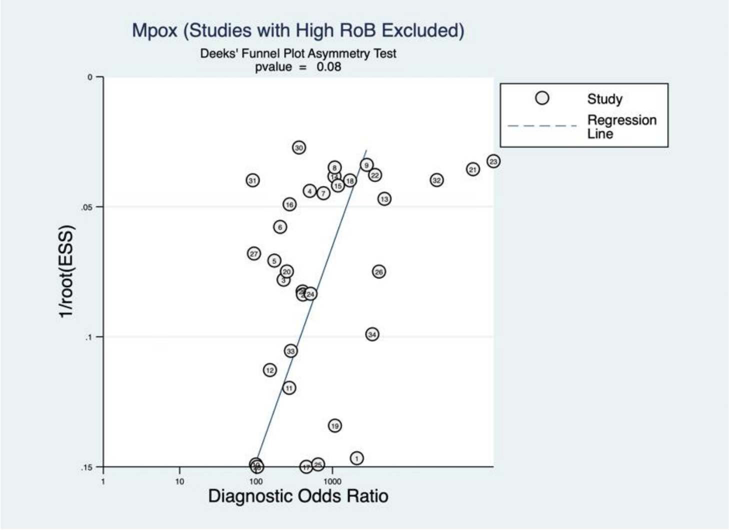

© 2025 Soe NN et al. *JAMA Network Open*.

B. **Herpes Simplex**

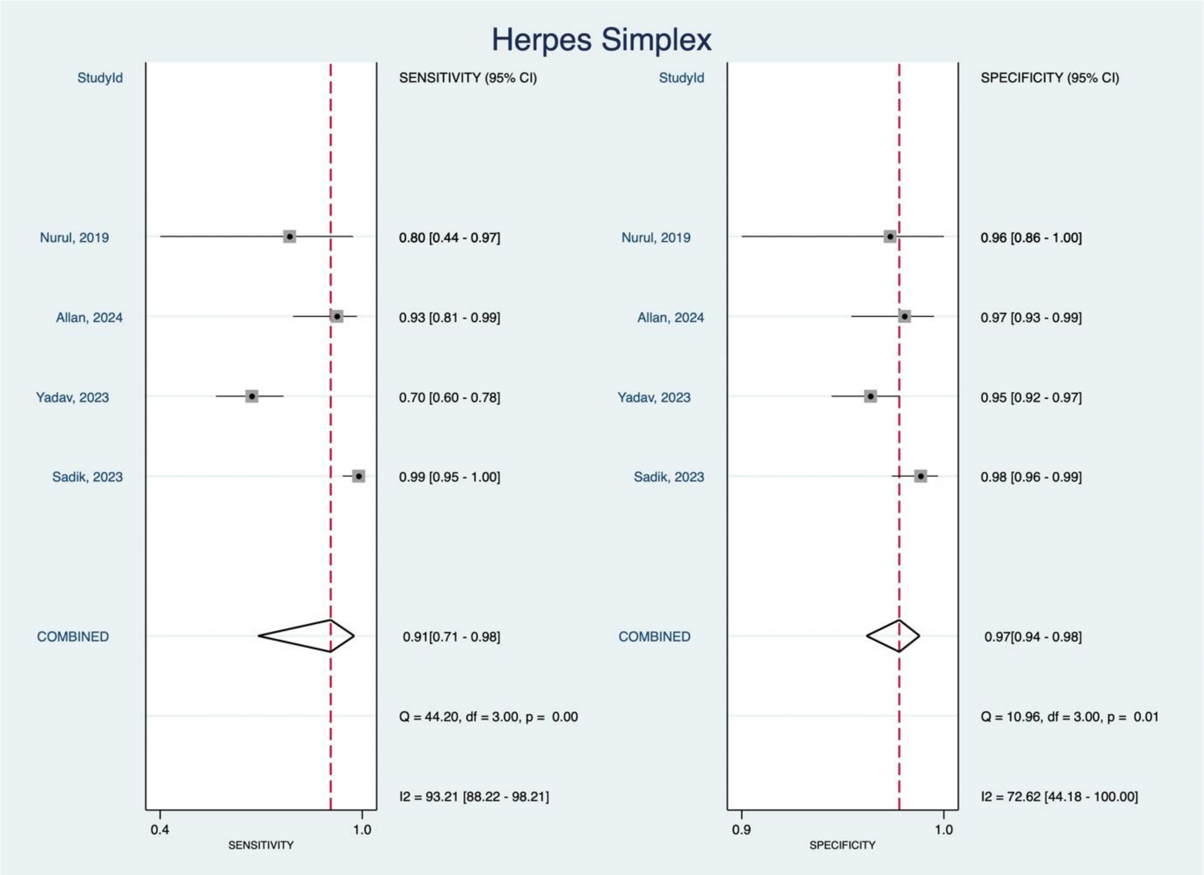

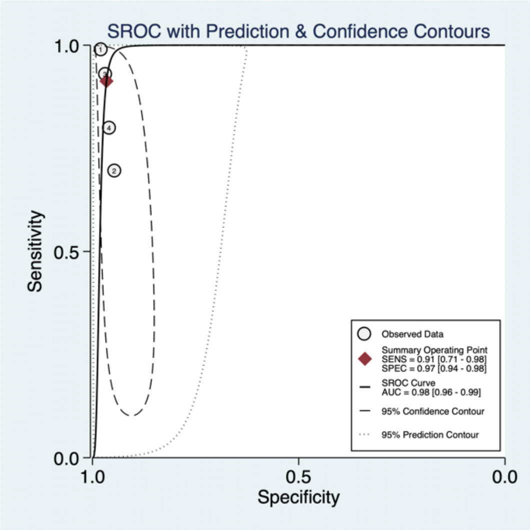

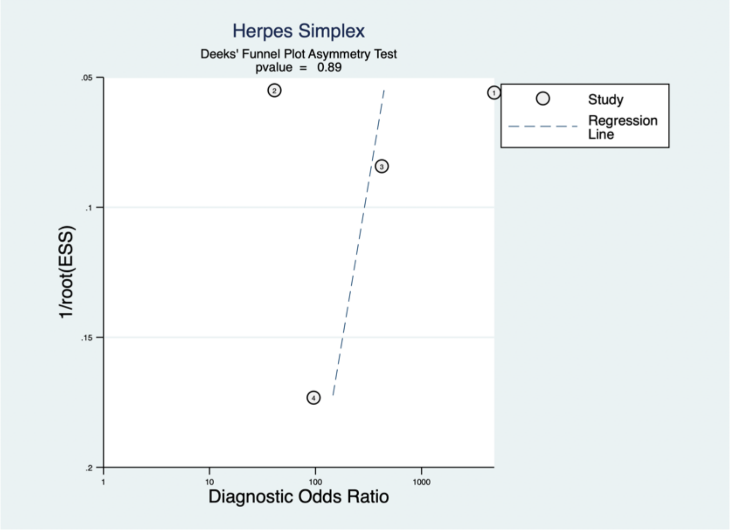

© 2025 Soe NN et al. *JAMA Network Open*.

C. **Genital Warts**

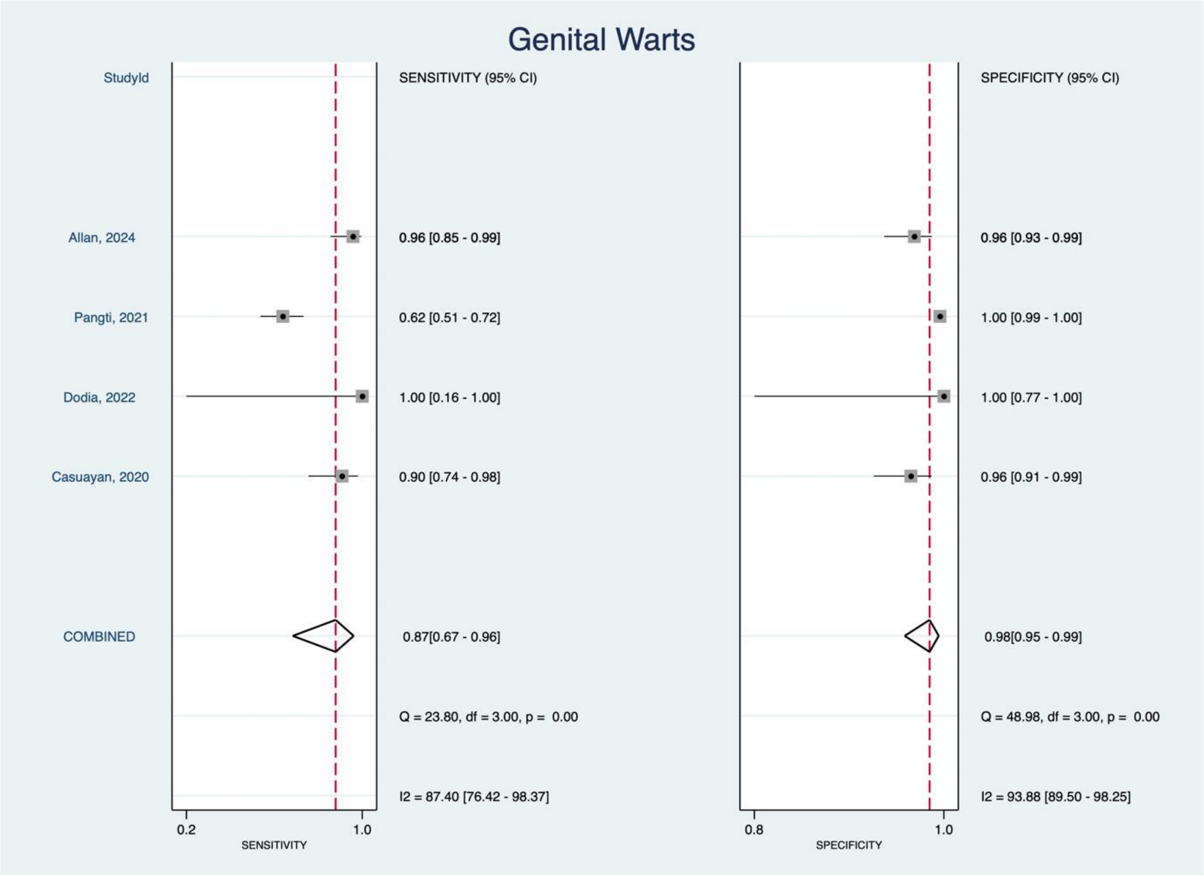

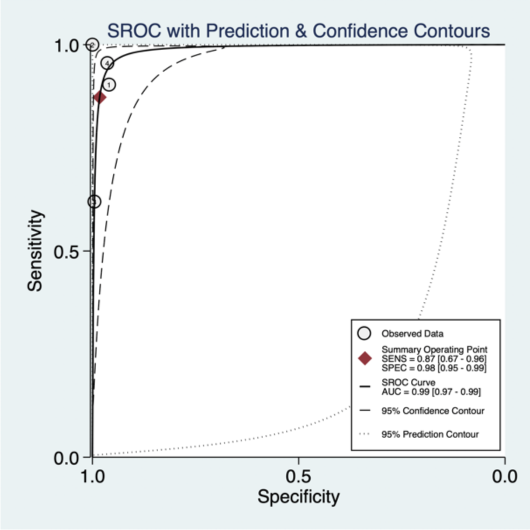

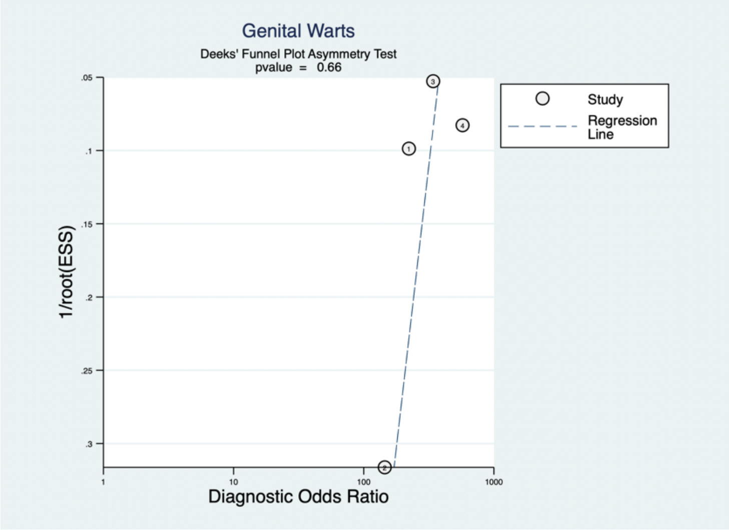

© 2025 Soe NN et al. *JAMA Network Open*.

D. **Psoriasis**

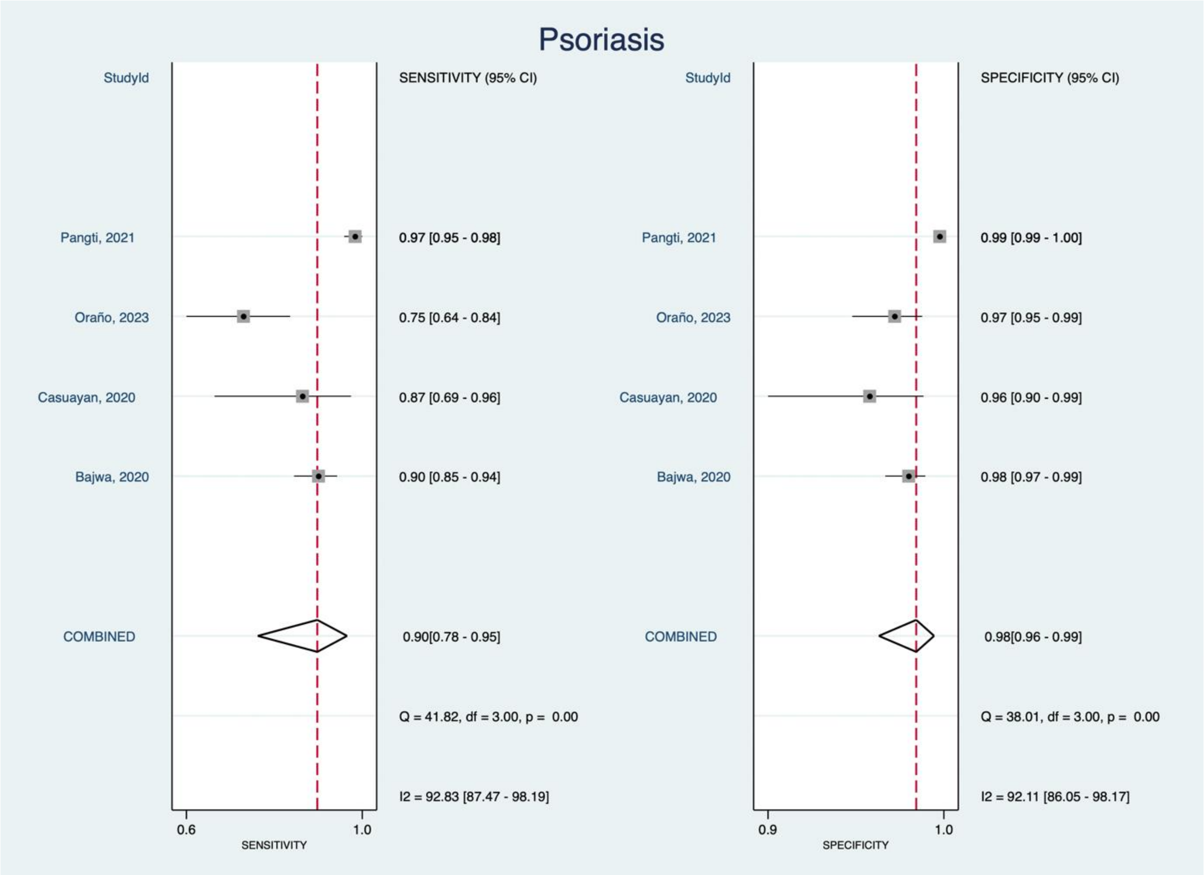

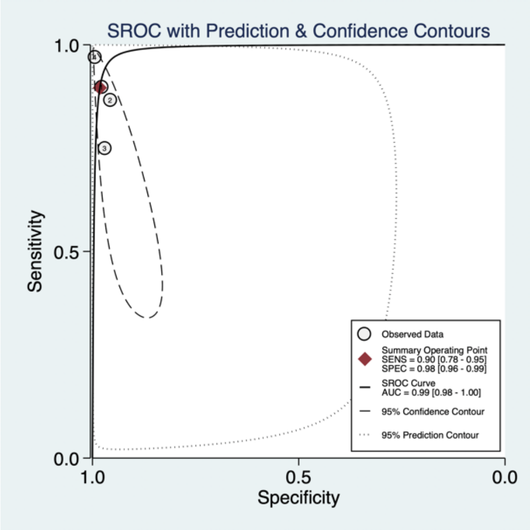

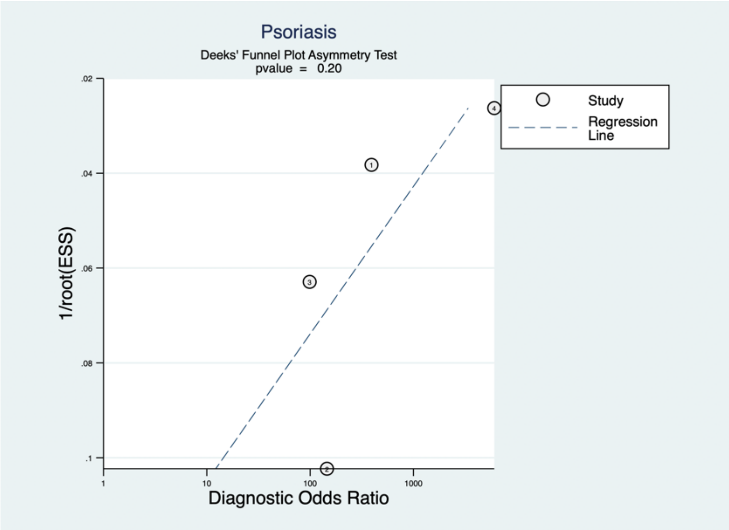

© 2025 Soe NN et al. *JAMA Network Open*.

E. **Scabies**

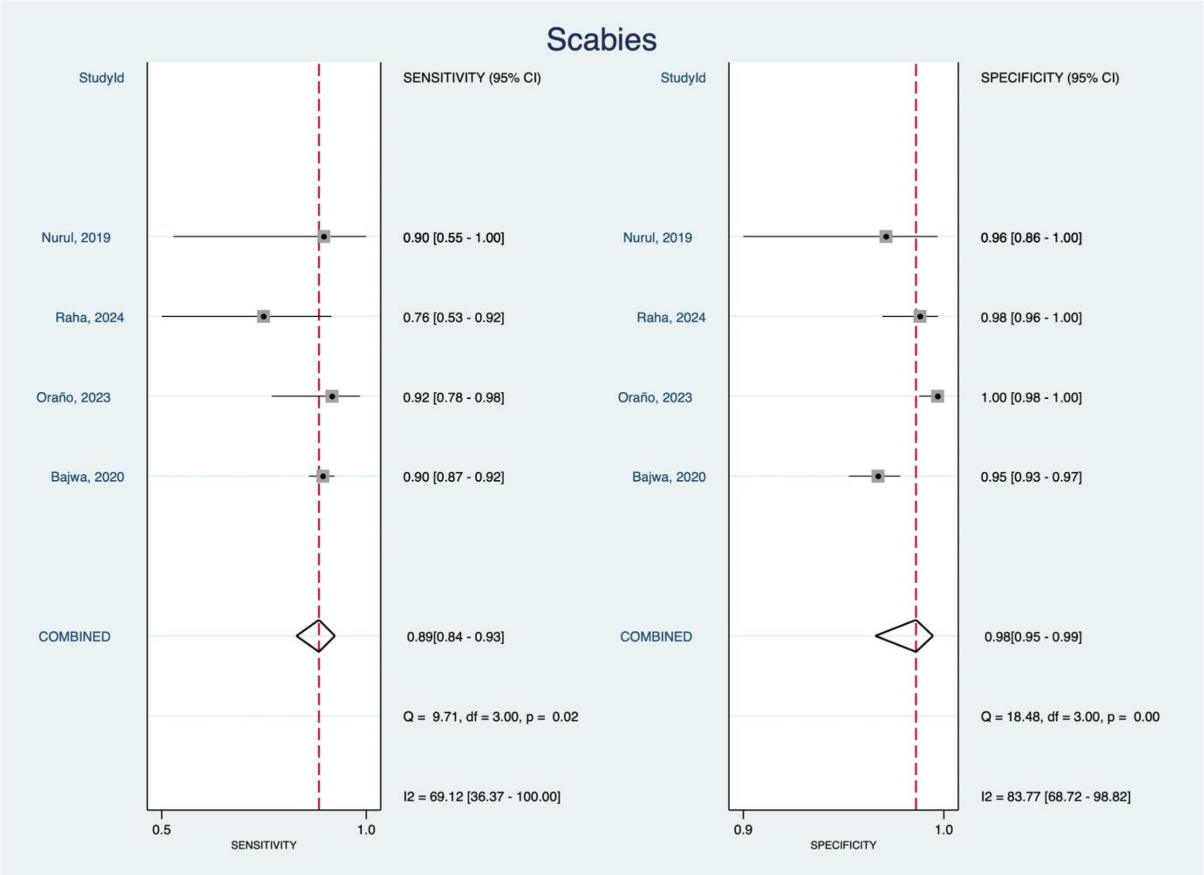

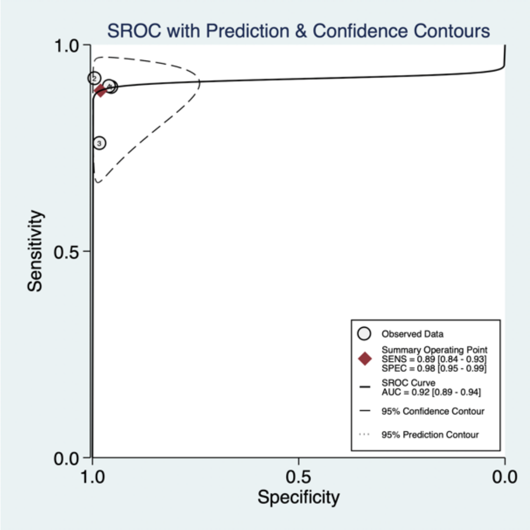

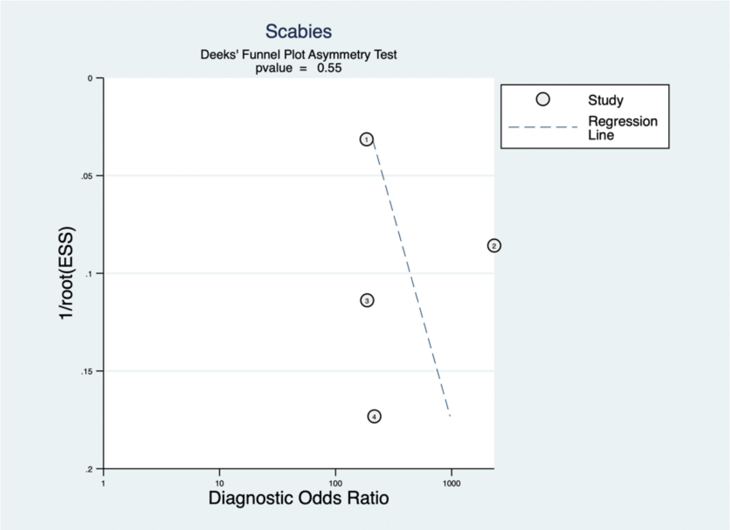

© 2025 Soe NN et al. *JAMA Network Open*.

eReferences

1. Allan-Blitz L-T, Ambepitiya S, Tirupathi R, Klausner JD: **The Development and Performance of a Machine-Learning Based Mobile Platform for Visually Determining the Etiology of 5 Penile Diseases**. *Mayo Clinic Proceedings: Digital Health* 2024, **2**(2):280-288.
2. Gaffoor N, Soomro S: **Skin Disease Detection and Classification Using ResNet-50 and Support Vector Machine: An Effective Approach for Dermatological Diagnosis**. In*: 2023*; 2023: 140-145.
3. Mehta N, Khan E, Choudhary R, Dholakia D, Goel S, Gupta S: **The performance of an artificial intelligence-based computer vision mobile application for the image diagnosis of genital dermatoses: a prospective cross-sectional study**. *International Journal of Dermatology* 2024((Mehta, Khan, Choudhary, Goel, Gupta) Department of Dermatology and Venereology, All India Institute of Medical Sciences, New Delhi, India(Dholakia) Rajiv Gandhi Cancer Institute and Research Centre, New Delhi, India).
4. Wei L-S, Gan Q, Ji T: **Skin Disease Recognition Method Based on Image Color and Texture Features**. *Computational and mathematical methods in medicine* 2018, **2018**(101277751):8145713.
5. Yadav A, Sharma V, Seth J: **Skin Disease Recognition by VGG-16 Model**. In*: 2023*; 2023: 833-847.
6. Akmalia N, Sihombing P, Suherman: **Skin Diseases Classification Using Local Binary Pattern and Convolutional Neural Network**. In: *2019 3rd International Conference on Electrical, Telecommunication and Computer Engineering (ELTICOM): 16-17 Sept. 2019 2019*; 2019: 168-173.
7. Sadik R, Majumder A, Biswas AA, Ahammad B, Rahman MM: **An in-depth analysis of Convolutional Neural Network architectures with transfer learning for skin disease diagnosis**. *Healthcare Analytics* 2023, **3**((Sadik, Majumder, Ahammad) Department of Computer Science and Engineering, Jahangirnagar University, Savar, Dhaka, Bangladesh(Biswas, Rahman) Department of Computer Science and Engineering, Daffodil International University, Dhaka, Bangladesh):100143.
8. Assoc Comp M, Nosseir A, Shawky MA: **Automatic Classifier for Skin Disease Using k-NN and SVM**. In.; 2019: 259-262.
9. Casuayan De Goma J, Devaraj M: **Recognizing Common Skin Diseases in the Philippines Using Image Processing and Machine Learning Classification**. In*: 2020*; 2020: 68-72.
10. Pangti R, Mathur J, Chouhan V, Kumar S, Rajput L, Shah S, Gupta A, Dixit A, Dholakia D, George M *et al*: **A machine learning-based, decision support, mobile phone application for diagnosis of common dermatological diseases**. *Journal of the European Academy of Dermatology and Venereology* 2021, **35**(2):536-545.
11. Zaar O, Larson A, Polesie S, Saleh K, Tarstedt M, Olives A, Suarez A, Gillstedt M, Neittaanmaki N: **Evaluation of the diagnostic accuracy of an online artificial intelligence application for skin disease diagnosis**. *Acta Dermato-Venereologica* 2020, **100**(16):1-6.

© 2025 Soe NN et al. *JAMA Network Open*.

12. Dodia D, Jakharia H, Soni R, Borade S, Jain N: **Human Skin Disease Detection using MLXG model**. In: *CVMLH-2022: Workshop on Computer Vision and Machine Learning for Healthcare: 2022*; 2022: 1-14.
13. Abdelhamid AA, El-Kenawy ESM, Khodadadi N, Mirjalili S, Khafaga DS, Alharbi AH, Ibrahim A, Eid MM, Saber M: **Classification of Monkeypox Images Based on Transfer Learning and the Al-Biruni Earth Radius Optimization Algorithm**. *MATHEMATICS* 2022, **10**(19).
14. Agrawal S, Castelino K, Mehta J, Bhavathankar P: **EfficientNet-B3 and Image Processing for Monkeypox Detection using Skin Lesion Images**. In*: 2022*; 2022.
15. Ahsan MM, Alam TE, Haque MA, Ali MS, Rifat RH, Nafi AAN, Hossain MM, Islam MK: **Enhancing Monkeypox diagnosis and explanation through modified transfer learning, vision transformers, and federated learning**. *Informatics in Medicine Unlocked* 2024, **45**((Ahsan, Alam) Department of Industrial and Systems Engineering, University of Oklahoma, Norman, OK 73019, United States(Haque) Department of Cyber-Physical Systems, Clark Atlanta University, Atlanta, GA 30314, United States(Ali, Hossain, Islam) Department):101449.
16. Ahsan MM, Ali MS, Hassan MM, Abdullah TA, Gupta KD, Bagci U, Kaushal C, Soliman NF: **Monkeypox Diagnosis With Interpretable Deep Learning**. *IEEE Access* 2023, **11**:81965-81980.
17. Ahsan MM, Uddin MR, Ali MS, Islam MK, Farjana M, Sakib AN, Al Momin K, Luna SA: **Deep transfer learning approaches for Monkeypox disease diagnosis**. *EXPERT SYSTEMS WITH APPLICATIONS* 2023, **216**.
18. Alcalá-Rmz V, Villagrana-Bañuelos KE, Celaya-Padilla JM, Galván-Tejada JI, Gamboa-Rosales H, Galván-Tejada CE: **Convolutional Neural Network for Monkeypox Detection**. In., vol. 594; 2023: 89-100.
19. Alharbi AH, Towfek SK, Abdelhamid AA, Ibrahim A, Eid MM, Khafaga DS, Khodadadi N, Abualigah L, Saber M: **Diagnosis of Monkeypox Disease Using Transfer Learning and Binary Advanced Dipper Throated Optimization Algorithm**. *BIOMIMETICS* 2023, **8**(3).
20. Alhasson HF, Almozainy E, Alharbi M, Almansour N, Alharbi SS, Khan RU: **A Deep Learning-Based Mobile Application for Monkeypox Detection**. *APPLIED SCIENCES-BASEL* 2023, **13**(23).
21. Almufareh MF, Tehsin S, Humayun M, Kausar S: **A Transfer Learning Approach for Clinical Detection Support of Monkeypox Skin Lesions**. *Diagnostics* 2023, **13**(8):1503.
22. Almutairi SA: **DL-MDF-OH²: Optimized Deep Learning-Based Monkeypox Diagnostic Framework Using the Metaheuristic Harris Hawks Optimizer Algorithm**. *ELECTRONICS* 2022, **11**(24).
23. Aloraini M: **An effective human monkeypox classification using vision transformer**. *INTERNATIONAL JOURNAL OF IMAGING SYSTEMS AND TECHNOLOGY* 2024, **34**(1).
24. Alrusaini OA: **Deep Learning Models for the Detection of Monkeypox Skin Lesion on Digital Skin Images**. *INTERNATIONAL JOURNAL OF ADVANCED COMPUTER SCIENCE AND APPLICATIONS* 2023, **14**(1):637-644.
25. Altun M, Guruler H, Ozkaraca O, Khan F, Khan J, Lee Y: **Monkeypox Detection Using CNN with Transfer Learning**. *Sensors (Basel, Switzerland)* 2023, **23**(4).

© 2025 Soe NN et al. *JAMA Network Open*.

26. Amin J, Gul N, Naqvi SA: **J-Net:Convolutional Neural Network based on Grey Binary Wolf Optimization Model for Classification of Skin Lesion**. In*: 2023*; 2023.
27. Arshed MA, Rehman HA, Ahmed S, Dewi C, Christanto HJ: **A 16 x 16 Patch-Based Deep Learning Model for the Early Prognosis of Monkeypox from Skin Color Images**. *COMPUTATION* 2024, **12**(2).
28. Asif S, Zhao M, Li Y, Tang F, Zhu Y: **CGO-ensemble: Chaos game optimization algorithm-based fusion of deep neural networks for accurate Mpox detection**. *Neural networks : the official journal of the International Neural Network Society* 2024, **173**(drv, 8805018):106183.
29. Asif S, Zhao M, Tang F, Zhu Y, Zhao B: **Metaheuristics optimization-based ensemble of deep neural networks for Mpox disease detection**. *Neural networks : the official journal of the International Neural Network Society* 2023, **167**(drv, 8805018):342-359.
30. Attallah O: **MonDiaL-CAD: Monkeypox diagnosis via selected hybrid CNNs unified with feature selection and ensemble learning**. *DIGITAL HEALTH* 2023, **9**.
31. Bala D, Hossain MS, Hossain MA, Abdullah MI, Rahman MM, Manavalan B, Gu N, Islam MS, Huang Z: **MonkeyNet: A robust deep convolutional neural network for monkeypox disease detection and classification**. *Neural networks : the official journal of the International Neural Network Society* 2023, **161**(drv, 8805018):757-775.
32. Bansal M, Arora R, Keshari S, Panchal S: **Discerning Monkeypox from Other Viruses of the Poxviridae Family in a Deep Learning Paradigm**. In*: 2023*; 2023: 23-42.
33. Bogar SM, Deshmukh P, Reddy CVR, Muvva S: **Monkeypox Detection using CNN-Based Pretrained Models**. In*: 2023*; 2023: 173-178.
34. Campana MG, Colussi M, Delmastro F, Mascetti S, Pagani E: **A Transfer Learning and Explainable Solution to Detect mpox from Smartphones images**. *PERVASIVE AND MOBILE COMPUTING* 2024, **98**.
35. Chandrahaas BV, Mohanty SN, Panda SK, Michael G: **An Empirical Study on Classification of Monkeypox Skin Lesion Detection**. *EAI Endorsed Transactions on Pervasive Health and Technology* 2023, **9**(1).
36. Chauhan N, Bala A: **Comparative performance analysis of CNN with Transfer Learning Models for Detection of Monkeypox Skin disease**. In: *2023 4th IEEE Global Conference for Advancement in Technology (GCAT): 2023*; 2023.
37. Chen B, Han Y, Yan L: **A Few-shot learning approach for Monkeypox recognition from a cross-domain perspective**. *Journal of biomedical informatics* 2023, **144**(100970413, d2m):104449.
38. Chen J, Han J: **A study on the recognition of monkeypox infection based on deep convolutional neural networks**. *Frontiers in immunology* 2023, **14**(101560960):1225557.
39. Chintamaneni V, Krishna BH, Suresh M, Bukkaptanm K, Sujatha CN, Swaraja K, Kumar PM: **Deep Learning-Based Diagnostic Model for Automated Detection of Monkeypox: Introducing MonkeypoxNet**. *TRAITEMENT DU SIGNAL* 2024, **41**(1):493-502.

© 2025 Soe NN et al. *JAMA Network Open*.

40. Ciran A, Ozbay E: **Optimization-Based Feature Selection in Deep Learning Methods for Monkeypox Skin Lesion Detection**. In: *2023 7th International Symposium on Multidisciplinary Studies and Innovative Technologies: 2023*; 2023.
41. Dahiya N, Sharma YK, Rani U, Hussain S, Nabilal KV, Mohan A, Nuristani N: **Hyper-parameter tuned deep learning approach for effective human monkeypox disease detection**. *Scientific reports* 2023, **13**(1):15930.
42. Dan R, Wu Q, Ji X, Gu R, Chen X, Jia G, Huang X, Ye X, Wang Y: **LSANet: Lesion-Specific Attention Network for Monkeypox Categorization**. In*: 2022*; 2022: 210-217.
43. Dwivedi M, Tiwari RG, Ujjwal N: **Deep Learning Methods for Early Detection of Monkeypox Skin Lesion**. In: *2022 8th International Conference on Signal Processing and Communication (ICSC): 2022*; 2022: 343-348.
44. Eliwa EHI, El Koshiry AM, Abd El-Hafeez T, Farghaly HM: **Utilizing convolutional neural networks to classify monkeypox skin lesions**. *Scientific reports* 2023, **13**(1):14495.
45. Ezenkwu CP, Stephen BUA, Affiah I, Daniel B: **A Green AI Model Selection Strategy for Computer-Aided Mpox Detection**. In: *2023 IEEE AFRICON: 2023*; 2023.
46. Fisranda F, Austin F, Sadrawi M: **Comparison Analysis of YOLO, ResNet, and EfficientNet for contagious skin diseases**. In*: 2023*; 2023.
47. Gairola AK, Kumar V: **Monkeypox Disease Diagnosis using Machine Learning Approach**. In*: 2022*; 2022: 423-427.
48. Gupta A, Bhagat M, Jain V: **Blockchain-enabled healthcare monitoring system for early Monkeypox detection**. *JOURNAL OF SUPERCOMPUTING* 2023, **79**(14):15675-15699.
49. Gupta P, Mittal U, Jha T, Agarwal M, Tiwari A: **Efficient Prediction and Analysis of Monkeypox Skin Lesion: A Comparative Study for Web based Application**. In*: 2023*; 2023.
50. Haque ME, Ahmed MR, Nila RS, Islam S: **Human Monkeypox Disease Detection Using Deep Learning and Attention Mechanisms**. In*: 2022*; 2022: 1069-1073.
51. Haque R, Sultana A, Haque P: **Ensemble of Fine-tuned Deep Learning Models for Monkeypox Detection: A Comparative Study**. In*: 2023*; 2023.
52. Haripriya KP, Hannah Inbarani H: **Performance Analysis of Various Feature Extraction Methods for Classification of Pox Virus Images**. In*: 2024*; 2024: 211-223.
53. Hossen MR, Alfaz N, Sami A, Tanim SA, Bin Sarwar T, Islam MK: **An EfficientNet to Classify Monkeypox-Comparable Skin Lesions Using Transfer Learning**. In*: 2023*; 2023.
54. Huong LH, Khang NH, Quynh L, Thang L, Canh DM, Sang HP: **A Proposed Approach for Monkeypox Classification**. *INTERNATIONAL JOURNAL OF ADVANCED COMPUTER SCIENCE AND APPLICATIONS* 2023, **14**(8):643-651.
55. Hussain SM, Ghouse S: **Detection and prediction of monkey pox disease by enhanced convolutional neural network approach**. *International Journal of Public Health Science* 2023, **12**(2):673-681.

© 2025 Soe NN et al. *JAMA Network Open*.

56. Ieee, Gupta K, Bajaj V: **A self-assessment framework for initial screening of human monkeypox**. In.; 2023: 168-173.
57. Ieee, Gürbüz S, Aydin G: **Monkeypox Skin Lesion Detection Using Deep Learning Models**. In.; 2022: 66-70.
58. Ieee, Irmak MC, Aydin T, Yaganoglu M: **Monkeypox Skin Lesion Detection with MobileNetV2 and VGGNet Models**. In.; 2022.
59. Ieee, Trang K, Nguyen AH, Thao NGM, Vuong BQ, Ton-That L: **Performance Enhancement in Pre-Trained Deep Learning Models for Monkeypox Skin Lesions Identification Using Feature Selection Algorithms**. In.; 2023: 56-61.
60. Islam T, Hussain, Chowdhury FUH, Islam BMR: **Can Artificial Intelligence Detect Monkeypox from Digital Skin Images?** *bioRxiv* 2022((Islam) Department of Computer Science and Engineering, Northern University Bangladesh, Dhaka 1215, Bangladesh(Hussain, Chowdhury) Department of Medicine, Dhaka Medical College Hospital, Dhaka 1000, Bangladesh(Islam) Health Information Unit, Directorate G).
61. Jahan N, Bajwa G, Akilan T: **Federated Learning-Assisted Self-supervised CNN for Monkeypox Diagnosis**. In*: 2023*; 2023.
62. Jaradat AS, Al Mamlook RE, Almakayeel N, Alharbe N, Almuflih AS, Nasayreh A, Gharaibeh H, Gharaibeh M, Gharaibeh A, Bzizi H: **Automated Monkeypox Skin Lesion Detection Using Deep Learning and Transfer Learning Techniques**. *International journal of environmental research and public health* 2023, **20**(5).
63. Kakulapati V: **Analysis of Monkey Pox (MPox) Detection Using UNETs and VGG16 Weights**. In*: 2023*; 2023: 321-332.
64. Kaushal C: **Monkeypox and other Skin Conditions Detection Utilizing CNN with VGG16 Transfered Language**. In*: 2023*; 2023: 931-938.
65. Khafaga DS, Ibrahim A, El-Kenawy ESM, Abdelhamid AA, Karim FK, Mirjalili S, Khodadadi N, Lim WH, Eid MM, Ghoneim ME: **An Al-Biruni Earth Radius Optimization-Based Deep Convolutional Neural Network for Classifying Monkeypox Disease**. *Diagnostics* 2022, **12**(11):2892.
66. Khan SUR, Asif S, Bilal O, Ali S: **Deep hybrid model for Mpox disease diagnosis from skin lesion images**. *INTERNATIONAL JOURNAL OF IMAGING SYSTEMS AND TECHNOLOGY* 2024, **34**(2).
67. Kottath AV, Malarvel M: **Comparison of Deep Learning Models for Monkeypox Disease Detection**. In*: 2023*; 2023.
68. Krishnan T, Selvakumar K, Vairachilai S: **Monkeypox Detection Through Watershed Segmentation and Appending 2D CNN Based Auto Encoder Monkeypox Detection Through CNN-Auto Encoder**. *International Journal on Recent and Innovation Trends in Computing and Communication* 2023, **11**(9s):598-606.
69. Krishnan T, Selvakumar K, Vairachilai S: **MULTI-TASK LEARNING FOR MONKEYPOX SKIN LESION SEGMENTATION AND CLASSIFICATION USING CNN AND ROOTSIFT**. *Journal of Theoretical and Applied Information Technology* 2024, **101**(1):339-349.
70. Krishnan VG, Liya BS, Lakshmi SV, Sathyamoorthy K, Ganesan S: **Monkeypox Detection Using Hyper-Parameter Tuned Based Transferable CNN Model**. *International Journal of Experimental Research and Review* 2023, **33**:18-29.

© 2025 Soe NN et al. *JAMA Network Open*.

71. Kumar A: **An XNOR-ResNet and spatial pyramid pooling-based YOLO v3-tiny algorithm for Monkeypox and similar skin disease detection**. *IMAGING SCIENCE JOURNAL* 2023, **71**(1):50-65.
72. Kumar V: **Analysis of CNN features with multiple machine learning classifiers in diagnosis of monkeypox from digital skin images**. *medRxiv* 2022((Kumar) Graphic Era Deemed to be University, India).
73. Kundu D, Rahman MM, Rahman A, Das D, Siddiqi UR, Alam MGR, Dey SK, Muhammad G, Ali Z: **Federated Deep Learning for Monkeypox Disease Detection on GAN-Augmented Dataset**. *IEEE ACCESS* 2024, **12**:32819-32829.
74. Kundu D, Siddiqi UR, Rahman MM: **Vision Transformer based Deep Learning Model for Monkeypox Detection**. In*: 2022*; 2022: 1021-1026.
75. Kundu NK, Karim M, Kobir S, Farid DM: **Attention Based Feature Fusion Network for Monkeypox Skin Leison Detection**. In*: 2023*; 2023.
76. Lakshmi M, Das R: **Classification of Monkeypox Images Using LIME-Enabled Investigation of Deep Convolutional Neural Network**. *DIAGNOSTICS* 2023, **13**(9).
77. Liu R: **Convolutional Siamese network-based few-shot learning for monkeypox detection under data scarcity**. In*: 2023*; 2023.
78. Liu W: **Implementation of detection of skin lesions in monkeypox based on a deep learning model - using an improved bilinear pooling model**. In*: 2023*; 2023.
79. Madhu HS, Rallapalli S, Thatikonda R: **Three Dimensional DenseUNet with CKHA Segmentation Technique for Monkeypox Disease Prediction**. In*: 2023*; 2023.
80. Magboo VPC, Magboo MSA: **Comparative Analysis of Optimization Algorithms Applied to Monkeypox Classification**. In*: 2023*; 2023.
81. Meena G, Mohbey KK, Kumar S: **Monkeypox recognition and prediction from visuals using deep transfer learning-based neural networks**. *Multimedia Tools and Applications* 2024.
82. Muduli D, Naidu AVSC, Durga KV, Rahul K, Kumar MJ, Sharma SK: **Enhancing Monkeypox Skin Lesion Detection: A Fusion Approach of VGG 16 and Xception Features with SVM Classifier**. In*: 2023*; 2023.
83. Munoz-Saavedra L, Escobar-Linero E, Civit-Masot J, Luna-Perejon F, Civit A, Dominguez-Morales M: **A Robust Ensemble of Convolutional Neural Networks for the Detection of Monkeypox Disease from Skin Images**. *Sensors (Basel, Switzerland)* 2023, **23**(16).
84. Nayak T, Chadaga K, Sampathila N, Mayrose H, Bairy GM, Prabhu S, Katta SS, Umakanth S: **Detection of Monkeypox from skin lesion images using deep learning networks and explainable artificial intelligence**. *APPLIED MATHEMATICS IN SCIENCE AND ENGINEERING* 2023, **31**(1).
85. Nayak T, Chadaga K, Sampathila N, Mayrose H, Gokulkrishnan N, Bairy GM, Prabhu S, S SK, Umakanth S: **Deep learning based detection of monkeypox virus using skin lesion images**. *Medicine in Novel Technology and Devices* 2023, **18**((Nayak, Sampathila, Mayrose, Gokulkrishnan, Bairy G) Department of Biomedical Engineering, Manipal Institute of Technology, Manipal Academy of Higher Education, Karnataka, Manipal 576104, India(Chadaga, Prabhu) Department of Computer Science and Engineeri):100243.

© 2025 Soe NN et al. *JAMA Network Open*.

86. Nazmee N, Ali MS, Mahmud S, Alam K, Chakrabarty A, Fahim-Ul-Islam M: **Enhancing Monkeypox Diagnosis: A Machine Learning Approach for Skin Lesion Classification**. In*: 2023*; 2023.
87. Ozaltin O, Yeniay O: **DETECTION OF MONKEYPOX DISEASE FROM SKIN LESION IMAGES USING MOBILENETV2 ARCHITECTURE**. *COMMUNICATIONS FACULTY OF SCIENCES UNIVERSITY OF ANKARA-SERIES A1 MATHEMATICS AND STATISTICS* 2023, **72**(2):482-499.
88. Oztel I, Oztel GY, Sahin VH: **Deep Learning-Based Skin Diseases Classification using Smartphones**. *ADVANCED INTELLIGENT SYSTEMS* 2023, **5**(12).
89. Pabbi V, Khullar V, Angurala M: **Efficient Skin Lesion based Classification System for Monkeypox Detection using VGG16 and Ensemble Learning**. In*: 2023*; 2023: 24-27.
90. Pal M, Mahal A, Mohapatra RK, Obaidullah AJ, Sahoo RN, Pattnaik G, Pattanaik S, Mishra S, Aljeldah M, Alissa M *et al*: **Deep and Transfer Learning Approaches for Automated Early Detection of Monkeypox (Mpox) Alongside Other Similar Skin Lesions and Their Classification**. *ACS OMEGA* 2023, **8**(35):31747-31757.
91. Pal S, Mishra AK: **Artificial Intelligence-Based Framework for Predicting Monkeypox Disease**. In*: 2023*; 2023.
92. Pasha MS, Poornima A, Athapuram D: **Skin Image Analysis for Monkeypox Diagnosis: Multi-Layer CNN Model Advancements**. *Journal of Cardiovascular Disease Research* 2023, **14**(2):2381-2393.
93. Pramanik A, Chowdhury F, Sultana S, Rahman MM, Bijoy MHI, Rahman MS: **Monkeypox Detection from Various Types of Poxes: A Deep Learning Approach**. In*: 2023*; 2023.
94. Pramanik R, Banerjee B, Efimenko G, Kaplun D, Sarkar R: **Monkeypox detection from skin lesion images using an amalgamation of CNN models aided with Beta function-based normalization scheme**. *PloS one* 2023, **18**(4):e0281815.
95. Prasher S, Nelson L, Gomathi S: **Pre-trained Deep learning model for Monkeypox Prediction using Dermoscopy Images in Healthcare**. In*: 2023*; 2023.
96. Raha AD, Gain M, Debnath R, Adhikary A, Qiao Y, Hassan MM, Bairagi AK, Islam SMS: **Attention to Monkeypox: An Interpretable Monkeypox Detection Technique Using Attention Mechanism**. *IEEE Access* 2024, **12**:51942-51965.
97. Rai S, Joshi RC, Dutta MK: **PoxDetector: A Deep Convolutional Neural Network for Skin Lesion Classification using Android Application**. In*: 2023*; 2023: 363-368.
98. Rao ML, Mahesh AV, Manikanth MNVJ, Sailaja J, Bhargavi KN: **MONKEYPOX DETECTION AND CLASSIFICATION USING MULTI-LAYER CONVOLUTIONAL NEURAL NETWORK FROM SKIN IMAGES**. *ARPN Journal of Engineering and Applied Sciences* 2023, **18**(21):2364-2379.
99. Ren GY: **Monkeypox Disease Detection with Pretrained Deep Learning Models**. *INFORMATION TECHNOLOGY AND CONTROL* 2023, **52**(2):288-296.
100. Sahin VH, Oztel I, Yolcu Oztel G: **Human Monkeypox Classification from Skin Lesion Images with Deep Pre-trained Network using Mobile Application**. *Journal of medical systems* 2022, **46**(11):79.
101. Sathwik AS, Naseeba B, Kiran JC, Lokesh K, Ch VSD, Challa NP: **Early Detection of Monkeypox Skin DiseaseUsing Patch Based DL Model and Transfer**

© 2025 Soe NN et al. *JAMA Network Open*.

Learning Techniques. *EAI Endorsed Transactions on Pervasive Health and Technology* 2023, **9**.
102. Shah A: **Monkeypox Skin Lesion Classification Using Transfer Learning Approach**. In*: 2022*; 2022.
103. Sharma A, Gupta S, Anand V: **Proposed Convolution Architecture for Monkeypox Detection using Dermoscopy Images**. In*: 2023*; 2023.
104. Sharma K, Kumar V, Mittal M: **MonkeyPox, Measles and ChickenPox Detection through Image-Processing using Residual Neural Network (ResNet)**. In*: 2023*; 2023.
105. Sharma N, Mohanty SN, Mahato S, Pattanaik CR: **A novel dataset and local interpretable model-agnostic explanations (LIME) for monkeypox prediction**. *INTELLIGENT DECISION TECHNOLOGIES-NETHERLANDS* 2023, **17**(4):1297-1308.
106. Singh U, Songare LS: **Analysis and Detection of Monkeypox using the GoogLeNet Model**. In*: 2022*; 2022: 1000-1008.
107. Soe NN, Yu Z, Latt PM, Lee D, Rahman R, Ge Z, Ong J, Fairley CK, Zhang L: **Distinguishing monkeypox from common skin lesions using artificial intelligence in a sexual health clinic: a feasibility study**. *Journal of Medical Internet Research* 2023, **20**(5):XVI-XVI.
108. Sorayaie Azar A, Naemi A, Babaei Rikan S, Bagherzadeh Mohasefi J, Pirnejad H, Wiil UK: **Monkeypox detection using deep neural networks**. *BMC infectious diseases* 2023, **23**(1):438.
109. Supanich W, Kulkarineetham S, Tamboonlertchai W, Suato T: **Classification of Mpox Images Using Transfer Learning on Lightweight Models**. In*: 2023*; 2023.
110. Surati S, Trivedi H, Shrimali B, Bhatt C, Travieso-González CM: **An Enhanced Diagnosis of Monkeypox Disease Using Deep Learning and a Novel Attention Model Senet on Diversified Dataset**. *MULTIMODAL TECHNOLOGIES AND INTERACTION* 2023, **7**(8).
111. Taruno PEN, Nugraha GS, Dwiyansaputra R, Bimantoro F: **Monkeypox Classification based on Skin Images using CNN: EfficientNet-B0**. In*: 2023*; 2023.
112. Thieme AH, Zheng Y, Machiraju G, Sadee C, Mittermaier M, Gertler M, Salinas JL, Srinivasan K, Gyawali P, Carrillo-Perez F *et al*: **A deep-learning algorithm to classify skin lesions from mpox virus infection**. *Nature medicine* 2023, **29**(3):738-747.
113. Thorat R, Gupta A: **Transfer learning-enabled skin disease classification: the case of monkeypox detection**. *MULTIMEDIA TOOLS AND APPLICATIONS* 2024.
114. Tiwari S, Maheshwari P: **MPox-DenseConvNet: A Transfer Learning Based Convolutional Neural Network for Monkeypox Detection and Assessment using Color Models**. In*: 2023*; 2023: 175-180.
115. Torky M, Bakheit A, Bakry M, Hassanien AE: **Deep learning Model for Recognizing Monkey Pox based on Dense net-121 Algorithm**. *medRxiv* 2022((Torky) Faculty of Artificial Intelligence, Egyptian Russian University, Egypt(Bakheit, Bakry) Higher Institute of Computer Science and Information Systems, Culture and Science City, 6th of October City 12573, Egypt(Hassanien) Faculty of Computers and Art).

© 2025 Soe NN et al. *JAMA Network Open*.

116. Ural AB: **A Computer-Aided Feasibility Implementation to Detect Monkeypox from Digital Skin Images with Using Deep Artificial Intelligence Methods**. *TRAITEMENT DU SIGNAL* 2023, **40**(1):383-388.
117. Uysal F: **Detection of Monkeypox Disease from Human Skin Images with a Hybrid Deep Learning Model**. *Diagnostics* 2023, **13**(10):1772.
118. Uzun Ozsahin D, Mustapha MT, Uzun B, Duwa B, Ozsahin I: **Computer-Aided Detection and Classification of Monkeypox and Chickenpox Lesion in Human Subjects Using Deep Learning Framework**. *Diagnostics* 2023, **13**(2):292.
119. Vajpayee H, Arora C, Voleti R, Chaudhary A, Raj G, Agrawal AP: **An effective classification of Skin Disease using Deep Learning Techniques**. In*: 2023*; 2023: 580-585.
120. Yadav S, Qidwai T: **Machine learning-based monkeypox virus image prognosis with feature selection and advanced statistical loss function**. *Medicine in Microecology* 2024, **19**((Yadav) Faculty of Biotechnology, Shri Ramswaroop Memorial University, Lucknow Deva Road, Uttar Pradesh 225003, India(Qidwai) Faculty of Biotechnology, Shri Ramswaroop Memorial University, Lucknow Deva Road, Uttar Pradesh 225003, India):100098.
121. Yasmin F, Hassan MM, Hasan M, Zaman S, Kaushal C, El-Shafai W, Soliman NF: **PoxNet22: A Fine-Tuned Model for the Classification of Monkeypox Disease Using Transfer Learning**. *IEEE Access* 2023, **11**:24053-24076.
122. Zi S: **Monkeypox Diagnosis with Convolutional Neural Networks Combined with Colour Space Models**. In*: 2022*; 2022: 1179-1184.
123. Danpakdee N, Songpan W: **Classification Model for Skin Lesion Image**. In., vol. 424; 2017: 553-561.
124. Bajwa UI, Alam S, Ul Haq N, Ratyal NI, Anwar MW: **Skin disease classification using neural network**. *Current Medical Imaging Reviews* 2020, **16**(6):711-719.
125. Hestiningsih I, Thohari ANA, Kamarudin ND: **Mobile Skin Disease Classification using MobileNetV2 and NASNetMobile**. *International Journal on Advanced Science, Engineering and Information Technology* 2023, **13**(4):1472-1479.
126. Liu Y, Jain A, Eng C, Way DH, Lee K, Bui P, Kanada K, de Oliveira Marinho G, Gallegos J, Gabriele S *et al*: **A deep learning system for differential diagnosis of skin diseases**. *Nature medicine* 2020, **26**(6):900-908.
127. Shen Y, Li H, Sun C, Ji H, Zhang D, Hu K, Tang Y, Chen Y, Wei Z, Lv J: **Optimizing skin disease diagnosis: harnessing online community data with contrastive learning and clustering techniques**. *npj Digital Medicine* 2024, **7**(1):28.
128. Chaurasia V, Pal S: **Skin diseases prediction: Binary classification machine learning and multi model ensemble techniques**. *Research Journal of Pharmacy and Technology* 2019, **12**(8):3829-3832.
129. Ieee, Rimi TA, Sultana N, Foysal MFA: **Derm-NN: Skin Diseases Detection Using Convolutional Neural Network**. In.; 2020: 1205-1209.
130. Andryani NAC, Juwono FH, Sianturi PJ: **ARTIFICIAL INTELLIGENCE APPROACH FOR AUTOMATIC MULTICLASS SKIN DISEASES IDENTIFICATION**. *JOURNAL OF ENGINEERING SCIENCE AND TECHNOLOGY* 2023, **18**:99-111.
131. Aziz MA, Pujiono P, Arief Soeleman M, Rosidin S: **Scabies Skin Classification Using SVM Method With GLCM Feature Extraction**. In: *International Seminar*

© 2025 Soe NN et al. *JAMA Network Open*.

on Application for Technology of Information and Communication: 2023; 2023: 221-226.
132. Oraño JFV, Padao FRF, Malangsa RD: **A Deep Convolutional Neural Network for Skin Rashes Classification**. In*: 2023*; 2023: 339-348.
133. Yotsu RR, Ding Z, Hamm J, Blanton RE: **Deep learning for AI-based diagnosis of skin-related neglected tropical diseases: A pilot study**. *PLoS neglected tropical diseases* 2023, **17**(8):e0011230.
134. Back S, Lee S, Shin S, Yu Y, Yuk T, Jong S, Ryu S, Lee K: **Robust Skin Disease Classification by Distilling Deep Neural Network Ensemble for the Mobile Diagnosis of Herpes Zoster**. *IEEE ACCESS* 2021, **9**:20156-20169.
135. Eze MC, Vafaei LE, Eze CT, Tursoy T, Ozsahin DU, Mustapha MT: **Development of a Novel Multi-Modal Contextual Fusion Model for Early Detection of Varicella Zoster Virus Skin Lesions in Human Subjects**. *PROCESSES* 2023, **11**(8).
136. Krammer S, Li Y, Jakob N, Boehm AS, Wolff H, Tang P, Lasser T, French LE, Hartmann D: **Deep learning-based classification of dermatological lesions given a limited amount of labelled data**. *Journal of the European Academy of Dermatology and Venereology* 2022, **36**(12):2516-2524.
137. Mejia Lara JV, Arias Velasquez RM: **Low-cost image analysis with convolutional neural network for herpes zoster**. *Biomedical Signal Processing and Control* 2022, **71**((Mejia Lara) Pontificia Universidad Catolica del Peru, Peru(Arias Velasquez) Universidad Tecnologica del Peru, Peru):103250.
138. Negi Y, Marimuthu P, Rauniyar NR, Patil US, Shaheen H: **Herpes Zoster Identification Using Optimized Deep Neural Network**. In*: 2024*; 2024: 361-370.
139. Soe NN, Yu Z, Latt PM, Lee D, Ong JJ, Ge Z, Fairley CK, Zhang L: **Evaluation of artificial intelligence-powered screening for sexually transmitted infections-related skin lesions using clinical images and metadata**. *BMC Med* 2024, **22**(1):296.
140. Daneshjou R, Barata C, Betz-Stablein B, Celebi ME, Codella N, Combalia M, Guitera P, Gutman D, Halpern A, Helba B *et al*: **Checklist for Evaluation of Image-Based Artificial Intelligence Reports in Dermatology: CLEAR Derm Consensus Guidelines From the International Skin Imaging Collaboration Artificial Intelligence Working Group**. *JAMA Dermatol* 2022, **158**(1):90-96.

© 2025 Soe NN et al. *JAMA Network Open*.
