# Supplementary material for: Use of AI in Identification of Sexually Transmitted Infections and Anogenital Dermatoses: A Systematic Review and Meta-Analysis
Source: JAMA Netw Open. 2025 Oct 3;8(10):e2533512. doi: 10.1001/jamanetworkopen.2025.33512 (PMC12495501; doi:10.1001/jamanetworkopen.2025.33512)
Supplement: Supplement 2. — Data Sharing Statement [file jamanetwopen-e2533512-s002.pdf]

Data Sharing Statement

Soe. Use of AI in Identification of Sexually Transmitted Infections and Anogenital Dermatoses. *JAMA Netw Open*. Published October 03, 2025. doi:10.1001/jamanetworkopen.2025.33512

Data
Data available: Yes
Data types: Data (not involving human participants)
How to access data: Data can be requested from the corresponding authors.
When available: With publication

Supporting Documents
Document types: Statistical/analytic code
How to access documents: Supporting documents can be requested from the corresponding authors.
When available: With publication

Additional Information
Who can access the data: researchers whose proposed use of the data
Types of analyses: for any purpose
Mechanisms of data availability: with investigator support
